# Adhesion to a common ECM mediates interdependence in tissue morphogenesis in *Drosophila*

**DOI:** 10.1038/s44319-026-00754-z

**Published:** 2026-04-01

**Authors:** Luis Eduardo Sánchez-Cisneros, Mariana Barrera-Velázquez, Dimitri Kromm, Philippe Bun, Horacio Merchant-Larios, Luis Daniel Ríos-Barrera

**Affiliations:** 1 Departamento de Biología Celular y Fisiología, Instituto de Investigaciones Biomédicas, Universidad Nacional Autónoma de México, Mexico City, Mexico; 2 Delft Center for Systems and Control, Faculty of Mechanical Engineering, Delft University of Technology, Delft, The Netherlands; 3 Université Paris Cité, Institute of Psychiatry and Neuroscience of Paris (IPNP), INSERM U1266, NeurImag Core Facility, Paris, France; 4 Present Address: Mechanobiology Institute, National University of Singapore, Singapore, Singapore; 5 Present Address: Cluster of Excellence “Physics of Life”, Technische Universität Dresden, Dresden, Germany

**Keywords:** Morphogenesis, Extracellular Matrix, Drosophila Embryogenesis, Dorsal Closure, Mechanotransduction, Cell Adhesion, Polarity & Cytoskeleton, Development, Signal Transduction

## Abstract

Organ functionality requires the precise coordination of diverse tissues during development. Halfway through *Drosophila* embryogenesis, two lateral epidermal sheets stretch to fuse at the dorsal midline; concomitant with this, the main tubes of the respiratory system also shift dorsally. Here, we demonstrate that these processes occur simultaneously and are coordinated by the adhesion of the epidermal sheets and a subset of cells of the tracheal trunks to a common extracellular matrix (ECM) that separates them. We also show that during dorsal closure, tracheal trunk cells extend protrusions towards the ECM underneath the epidermis. These protrusions are under tension, suggesting that they have a mechanical function. Additionally, perturbing adhesion between tracheal cells and the epidermis affects the development of both tissues. Altogether, our findings uncover a mechanism used for tissue coordination during development, one that is based on tissue adhesion towards a common ECM capable of transmitting mechanical forces across the embryo.

## Synopsis

During Drosophila development, tracheal dorsal trunk displacement and epidermal dorsal closure are coupled via adhesion to a common ECM. In consequence, perturbing the normal development of either tissue or the extracellular matrix between them, causes reciprocal malformations.

- Drosophila epidermal dorsal closure and tracheal dorsal trunk displacement are actively coordinated.
- Epidermal-tracheal trunk interactions are mediated by specialised cells from both tissues that adhere to a common ECM.
- Adhesion between tracheal and epidermal cells is required for proper epidermal dorsal closure and tracheal trunk development.
- Pulling forces from dorsal branches are dispensable for trunk displacement, but they are able to pull on the trunks when adhesion to the epidermis is perturbed.

## Abstract (web summary)

During Drosophila development, tracheal dorsal trunk displacement and epidermal dorsal closure are coupled via adhesion to a common ECM. In consequence, perturbing the normal development of either tissue or the extracellular matrix between them, causes reciprocal malformations.

## Graphical abstract

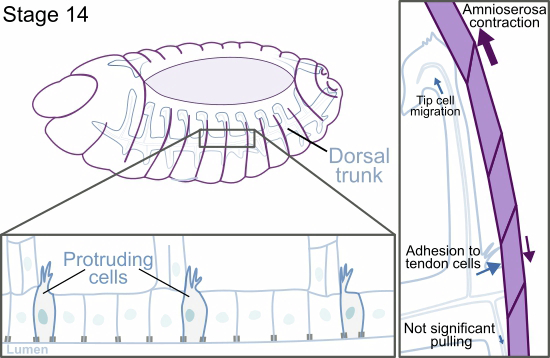

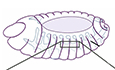

## Introduction

Cells and tissues modify their behaviour according to extracellular signals. Many works have characterised the intracellular mediators and effectors of such signalling pathways and how they influence individual cell behaviour, leading to tissue-wide rearrangements. However, fewer studies have examined how seemingly independent tissues influence each other’s behaviours. The development of the *Drosophila* tracheal system is a very useful model for looking at tissue interactions and how they influence cell and tissue remodelling, since tracheal tubes branch and migrate over neighbouring tissues of different biochemical and mechanical properties (Boube et al, 2001; Franch-Marro and Casanova, 2000; Ghabrial and Krasnow, 2006; Hayashi and Kondo, 2018; Urbano et al, 2011). The variety of environments that tracheal cells are exposed to suggests that the tracheal system has wide plasticity to adapt to specific conditions.

Tracheal development is orchestrated by Branchless (Bnl), a member of the fibroblast growth factor (FGF) family. Bnl is expressed in various tissues within the embryo, where it acts as a chemoattractant for tracheal tip cells. Initially, Bnl signals to tracheal pits on the sides of the embryo, inducing primary branching and fusion to form the main tubes of the tracheal system, the dorsal trunks. Dorsal trunks are two multicellular tubes that early in development localise to the sides of the embryo. Bnl also controls branch outgrowth from these trunks: dorsal branches grow towards the dorsal midline, whereas ventral branches grow towards the nervous system and the gut ((Samakovlis et al, 1996; Sutherland et al, 1996), reviewed in (Hayashi and Kondo, 2018)). It is in late tracheal development that trunks are displaced dorsally; however, the mechanisms behind trunk displacement remain unknown. Since trunk cells do not show hallmarks of Bnl signalling activation, and Bnl signalling becomes restricted to tracheal tip cells (Llimargas, 1999; Miao and Hayashi, 2016), it is likely that this repositioning is driven by trunk-extrinsic forces.

Trunk displacement coincides with branch outgrowth and epidermal dorsal closure, raising the question of whether forces from these processes contribute to trunk repositioning. During dorsal branch formation, tip cells migrate in response to Bnl, promoting stalk cell intercalation and branch elongation (Caussinus et al, 2008). Parallel to this, the epidermis undergoes dorsal closure, a process in which the two lateral epidermal sheets stretch and fuse at the midline. The epidermis is pulled by the amnioserosa, an epithelium attached to the dorsal epidermis that generates force through actomyosin contraction (Duque and Gorfinkiel, 2016; Kiehart et al, 2000; Pasakarnis et al, 2016).

We show here that epidermal dorsal closure and dorsal branch migration participate in proper tracheal trunk displacement. In addition, our experiments indicate that perturbing interactions between tracheal trunks and the epidermis also affects epidermal dorsal closure. Altogether, these results contribute to our understanding of how tissue development is integrated at the scale of the whole embryo, with multiple forces and adhesion between neighbouring tissues coordinating proper morphogenesis.

## Results

### Epidermis and tracheal trunks displace dorsally at the same time during embryogenesis

**Figure 1:**
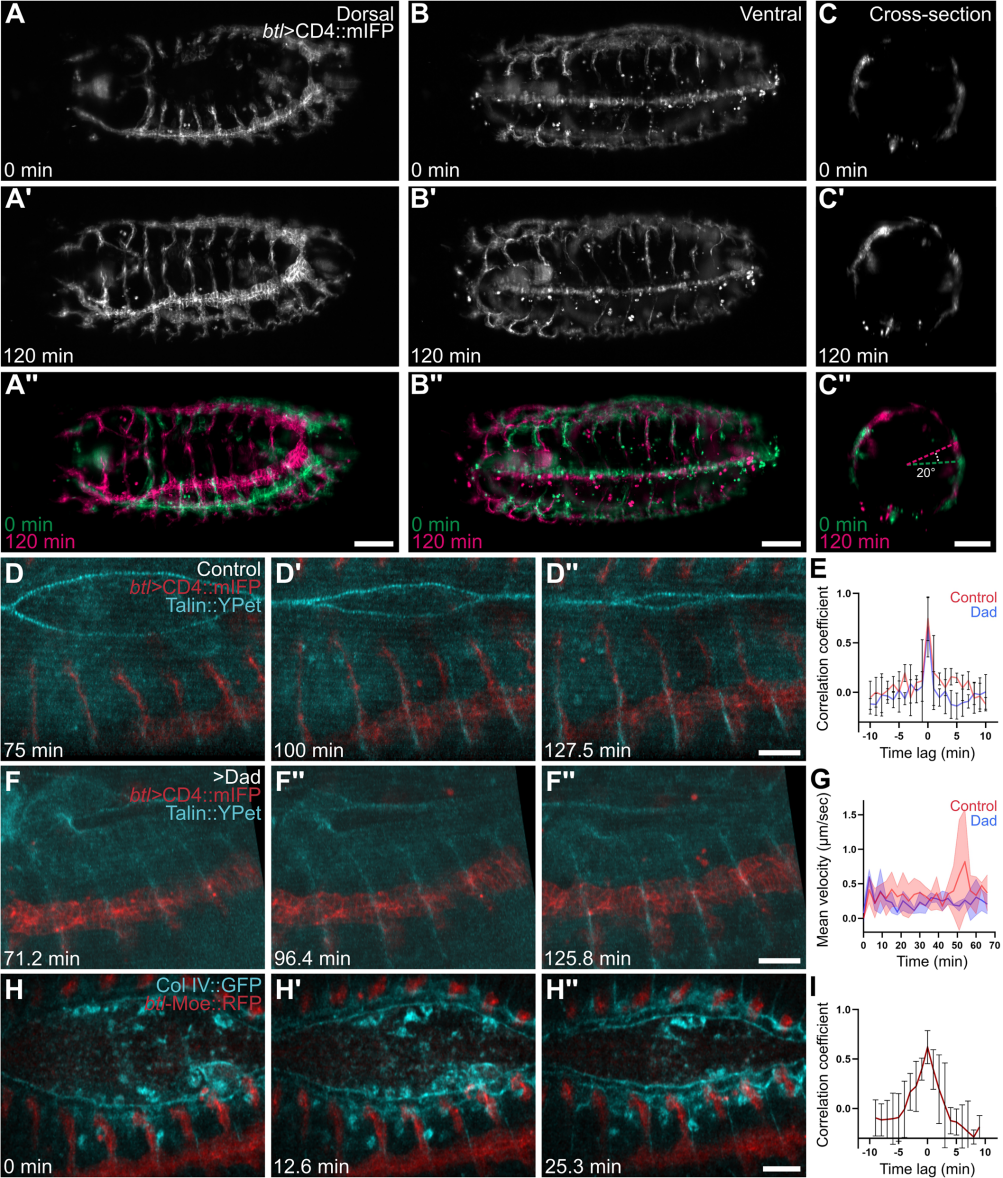
Coordination analyses of tracheal, epidermal and ECM movements during dorsal closure stages. (**A**–**C’**) Time-lapse imaging of tracheal development using MuVi-SPIM. The tracheal system is marked with *btl* > CD4::mIFP. (**A**–**A’**) Dorsal view; (**B**–**B’**) Ventral view; (**C**–**C’**) Transversal view. Cross-section was done between the 5th and 6th branch from anterior to posterior with respect to (**A**). (**A”**–**C”**) Overposition of the initial (green) and final (pink) imaging time point of the movie. (**D**–**D”**) Cartographical maximum intensity projection of confocal stacks of embryos expressing Talin::YPet (cyan) and CD4::mIFP under *btl-gal4* (red). (**E**) Cross-correlation analysis of the signal displacement of Talin::YPet and *btl* > CD4::mIFP in Control (*n* = 4) and Dad (*n* = 5) embryos. (**F**–**F”**) Cartographical maximum intensity projection of confocal stacks of embryos expressing Talin::YPet (cyan), and CD4::mIFP and Dad under *btl-gal4* (red). (**G**) Velocity of trunk displacement in embryos expressing *btl* > CD4::mIFP (*n* = 3) and embryos additionally expressing Dad (*n* = 3). (**H**–**H”**) Cartographical maximum intensity projection of confocal stacks of embryos expressing Col IV::GFP (cyan) and *btl*-Moe::RFP (red). (**I**) Cross-correlation analysis of the signal displacement of Col IV::GFP and *btl*-Moe::RFP throughout time, *n* = 5. (**A**–**C**) Scale bars: 50 µm. (**D**, **F**, **H**) Scale bars: 25 µm. Data are plotted as mean ± SD. Source data are available online for this figure.

Halfway through development, tracheal trunks displace from lateral to dorsal to acquire their final position, but the mechanisms behind this movement have not been described. Simultaneously with trunk displacement, dorsal branches elongate in response to Bnl (Fig. 1A–A”; Movie EV1). Only the tip cells respond to Bnl, and it was previously shown that the force generated by tip cell migration allows stalk cell intercalation and branch elongation (Caussinus et al, 2008). However, whether the force generated by tip cell migration is sufficient to drive dorsal trunk displacement has not been determined. In addition, at the same time as dorsal branches elongate, ganglionic and visceral branches extend ventrally and could also exert forces on the trunks (Fig. 1B–B”; Movie EV1). With branches growing out of the trunks in opposite directions (Fig. 1C–C”), other factors likely participate in their dorsal displacement, but so far these have remained unstudied.

**Figure EV1:**
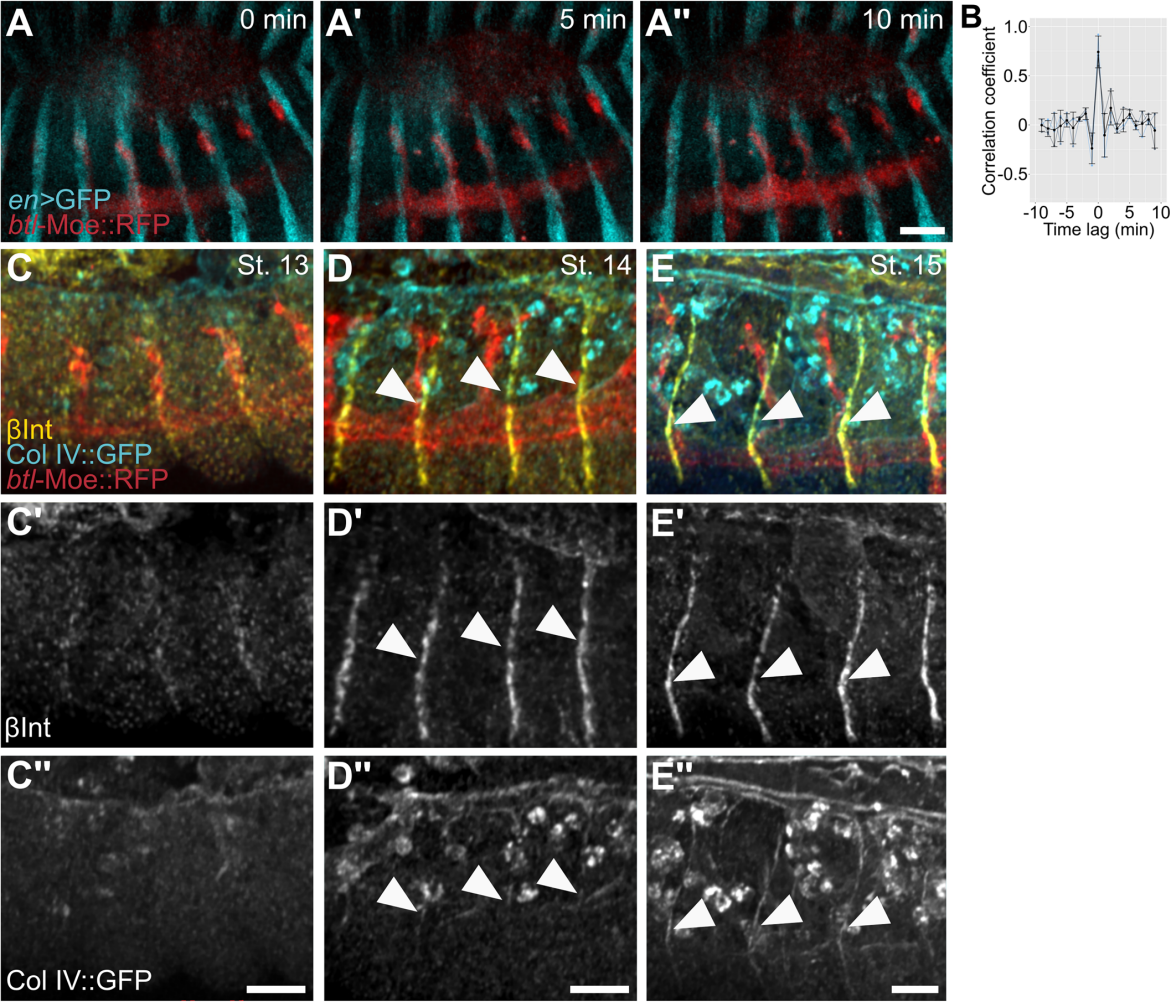
Analysis of epidermis-trachea coordination and distribution of Collagen IV::GFP and β-Integrin. (**A**–**A”**) Cartographical maximum intensity projection of confocal stacks showing stripes of epidermis using *en* > GFP (cyan) and the tracheal system using *btl*-Moe::RFP (red). (**B**) Cross-correlation analysis of *en* > GFP and *btl-*Moe::RFP signal displacement, *n* = 3 embryos. (**C**–**E**) Distribution of Collagen IV:: GFP (cyan) and β-Integrin (yellow) in stages 13–15 in relation to the tracheal system labelled with *btl-*MoeRFP (red). (**C**–**C”**) Stage 13; (**D**–**D”**) Stage 14; (**E**–**E”**) Stage 15. Arrowheads point to muscle attachment sites at their intersection with tracheal trunks. Data are plotted as mean ± SD. (**A**) Scale bar: 25 µm. (**C**–**E**) Scale bars: 20 µm. Source data are available online for this figure

Tracheal trunk displacement occurs at similar stages to epidermal dorsal closure, so we wondered if these two processes could be actively coordinated. To study this, we first carried out double-tissue labelling and live imaging, following the Moesin actin-binding domain fused to a red fluorescent protein expressed under the direct control of the tracheal-specific promoter of *btl* (*btl-*Moe::RFP). We used the Gal4*/UAS* system to label spaced epidermal stripes using GFP (*en* > GFP). In these recordings, we confirmed that trunk displacement coincided with epidermal dorsal closure (Fig. EV1A–A”). We quantified this observation by performing PIV analyses over consecutive time points for each of the tissues, followed by cross-correlation analysis of both tissue displacements. We found a very strong positive correlation (Fig. EV1B), suggesting that epidermal dorsal closure and trunk displacement are actively coordinated. We corroborated these measurements by following Talin endogenously tagged with YPet at the C-terminus (Talin::YPet) and an infrared fluorescent protein fused to CD4 under *btl-gal4* (*btl* > mCD4::mIFP), finding similar results (Fig. 1D–D”,E; Movie EV2).

Since dorsal branches elongate at the same time as dorsal closure and tip cells migrate right behind the leading edge of the epidermis, it was still possible that trunk displacement was coordinated with epidermal dorsal closure indirectly, as a consequence of branches pulling on the trunks as they migrate behind the epidermal leading edge. To test the role of dorsal branch migration in trunk displacement, we overexpressed the inhibitory SMAD, Daughters against dpp (Dad), in the tracheal system using *btl-gal4* (Fig. 1F–F”; Movie EV2). Dad overexpression prevents Decapentaplegic (Dpp) signalling activation, a condition where dorsal branches are not formed (Ribeiro et al, 2004). We found that in the absence of dorsal branches, the trunks still moved dorsally in coordination with the epidermis and at the same velocity as in controls (Fig. 1E,G; Movie EV2). This supports the conclusion that dorsal branches are dispensable for this movement and, in addition, it suggests that ventral branches do not pull on the trunks, as in the absence of dorsal branches, we still see the trunks moving dorsally.

The dorsal displacements of the trunks and the epidermis could be controlled by extracellular signals that act on both tissues. However, while signals from the epidermis participate in tracheal development, these act mainly in fate determination or tip cell migration (Affolter et al, 1994; Chihara and Hayashi, 2000; Glazer and Shilo, 2001; Kato et al, 2004; Llimargas, 2000). Similarly, the signals that regulate epidermal dorsal closure do not participate in tracheal development, namely, JNK signalling (Letizia et al, 2023). Therefore, we hypothesised that coordinated displacement could be mediated by adhesion, as the tracheal system lies beneath the epidermis. Tissue adhesion could be mediated by an intermediate extracellular matrix (ECM) layer. To explore this, we performed time-lapse microscopy of the tracheal system together with an ECM marker; an endogenous insertion of GFP into the *viking* locus, which codes for Collagen IV α2 subunit (from now on referred to as Col IV::GFP, Fig. 1H–H”). Col IV::GFP was enriched surrounding cardioblasts, hemocytes, and at muscle attachment sites, as reported previously (Borchiellini et al, 1996). Consistent with our findings with epidermal markers, the signal displacements of Col IV::GFP and the tracheal reporter were positively correlated (Fig. 1I).

### Tracheal trunk cells contact tendon cells of the epidermis during dorsal closure

Since we could not detect a particular enrichment of Col IV::GFP around the trunks using live imaging, we stained embryos for β-integrin and for GFP to detect Col IV::GFP. Before dorsal closure, we could not detect any enrichment of these components near the tracheal trunks (Fig. EV1C–C”). During the stages of dorsal closure, Col IV::GFP and β-integrin were visible at muscle attachment sites but not in the tracheal system (Fig. EV1D–D”). Only after this process was completed, we could detect a Col IV::GFP enrichment around the dorsal trunks, as has been reported already (Fig. EV1E–E”; (Klußmann-Fricke et al, 2022)).

**Figure 2:**
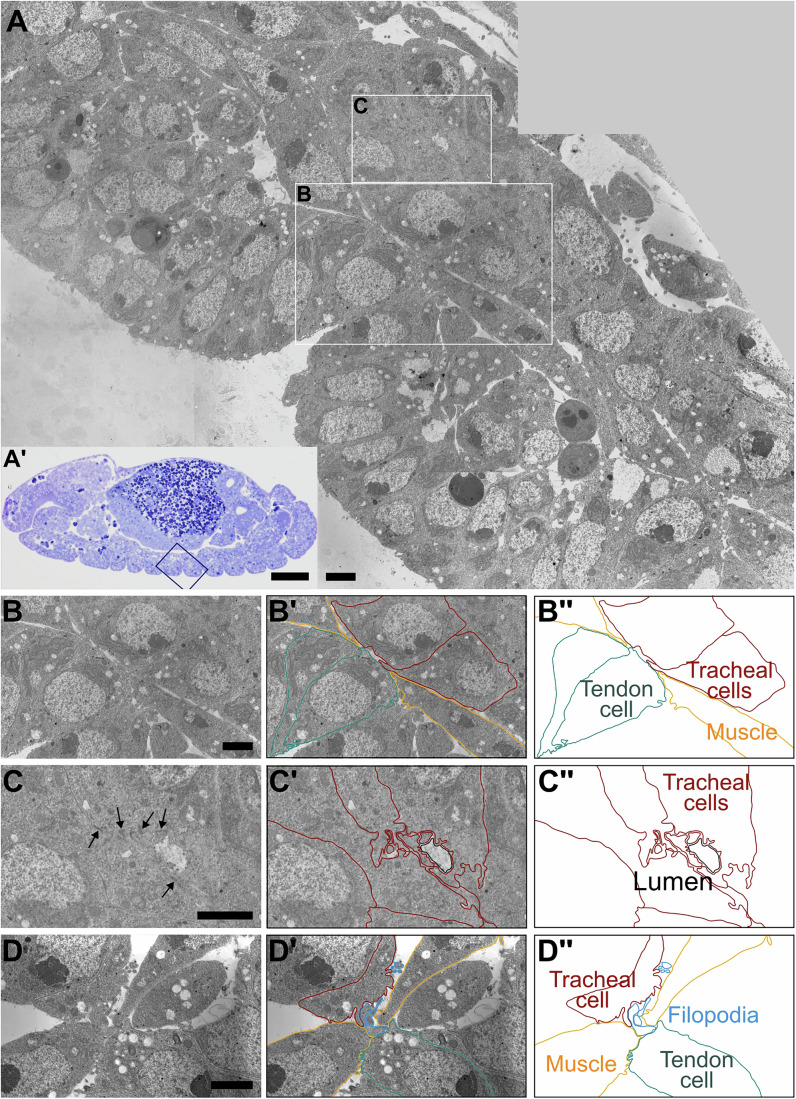
Contact points between the epidermis and the tracheal trunks. (**A**) Electron micrograph of a longitudinal section of the dorsal trunk, stage 14 embryo. The regions indicated by the white squares are magnified in (B and C). (**A’**) Semi-thin histological section used to identify the region of interest presented in (**A**), see “Methods”. The region indicated by the black square corresponds to the electron micrograph in (**A**). (**B**) Magnification of a contact point between tracheal trunks and a tendon cell. (**B’**, **B”**) Manual tracings of the elements present in (**B**). (**C**) Magnification of a region of the tracheal trunk where the lumen is visible, arrows point to electron-dense areas that correspond to adherens junctions. (**C’**, **C”**) Manual tracings of the elements present in (**C**). (**D**) Electron micrograph of tracheal cells in proximity to tendon cells and muscles. (**D’**, **D”**) Manual tracings of the elements present in (**D**). (**A**–**D**) Scale bars: 2 µm. (**A’**) Scale bar: 50 µm.

**Figure EV2:**
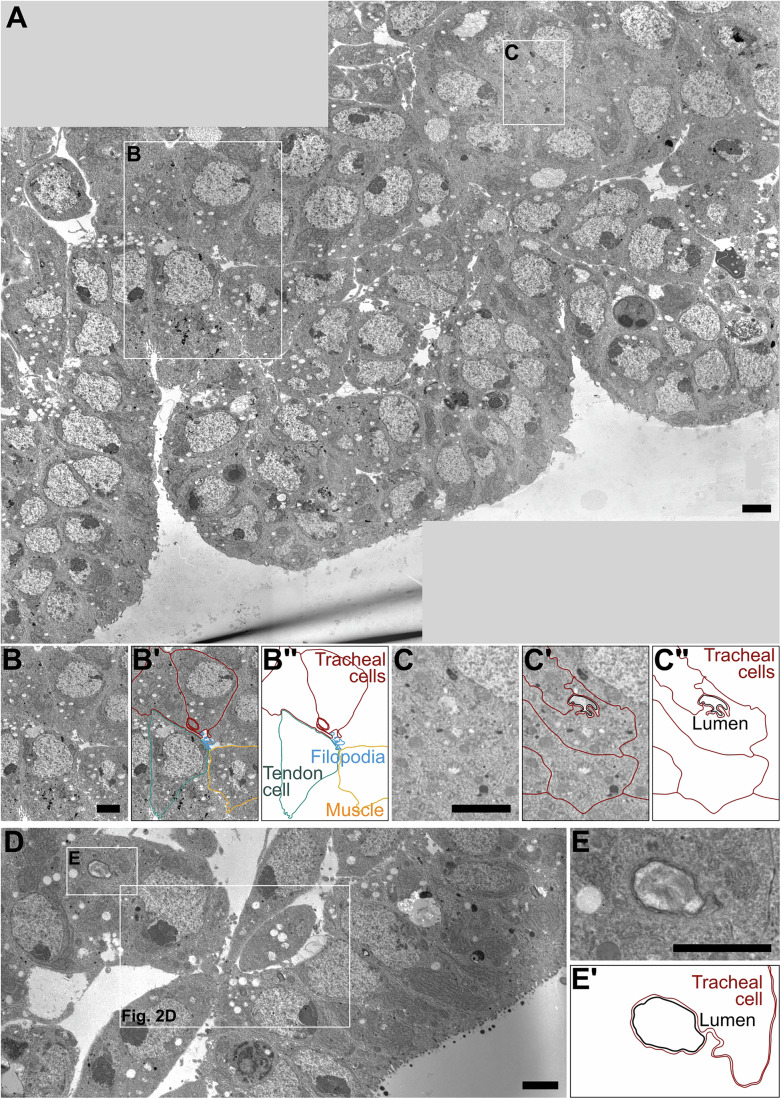
Contact points between the epidermis and the tracheal trunks (other examples). (**A**) Electron micrograph of a longitudinal section of the dorsal trunk, stage 14 embryo. The regions indicated by the white squares are magnified in (B and C). (**B**) Magnification of a contact point between tracheal trunks and a tendon cell. (**B’**, **B”**) Manual tracings of the elements present in (**B**). (**C**) Magnification of a region of the tracheal trunk where the lumen is visible. (**C’**, **C”**) Manual tracings of the elements present in (**C**). (**D**) Overview image of the micrograph magnified in Fig. 2D. (**E**) Magnified view of the tracheal lumen illustrated in (**D**). (**E’**) Manual tracings of the elements present in (**E**). Scale bars: 2 µm.

Muscles attach to epidermal tendon cells through a specialised meshwork of ECM (Fogerty et al, 1994; Prokop et al, 1998; Urbano et al, 2011); we reasoned that tracheal cells could also interact with the epidermis at these points. Classical anatomical descriptions of the embryo report that muscles separate the tracheal trunks from the epidermis (Hartenstein, 1993). Nevertheless, we decided to obtain higher-resolution images of these regions by using transmission electron microscopy. With this approach, we observed that, as reported, the dorsal trunks and the epidermis are separated by muscles and other mesenchymal cells in most parts of the embryo (Figs. 2A and EV2A). However, in the regions where tendon cells are found, trunks are no longer separated from the epidermis by other tissues but are instead found adjacent to the epidermis (Figs. 2B–B” and EV2B–B”). Tendon cells are easily recognisable in these images, as they localise at the segmental borders and associate with muscles. We identified tracheal cells based on their epithelial morphology and the presence of a lumen within the epithelium (Figs. 2C–C” and EV2C; one more example in EV2D,E). These experiments revealed a novel anatomical contact between the epidermis and tracheal trunks that had not been previously reported and whose relevance had not been studied until now.

### A subpopulation of dorsal trunk cells forms tensile protrusions towards the epidermis

**Figure 3:**
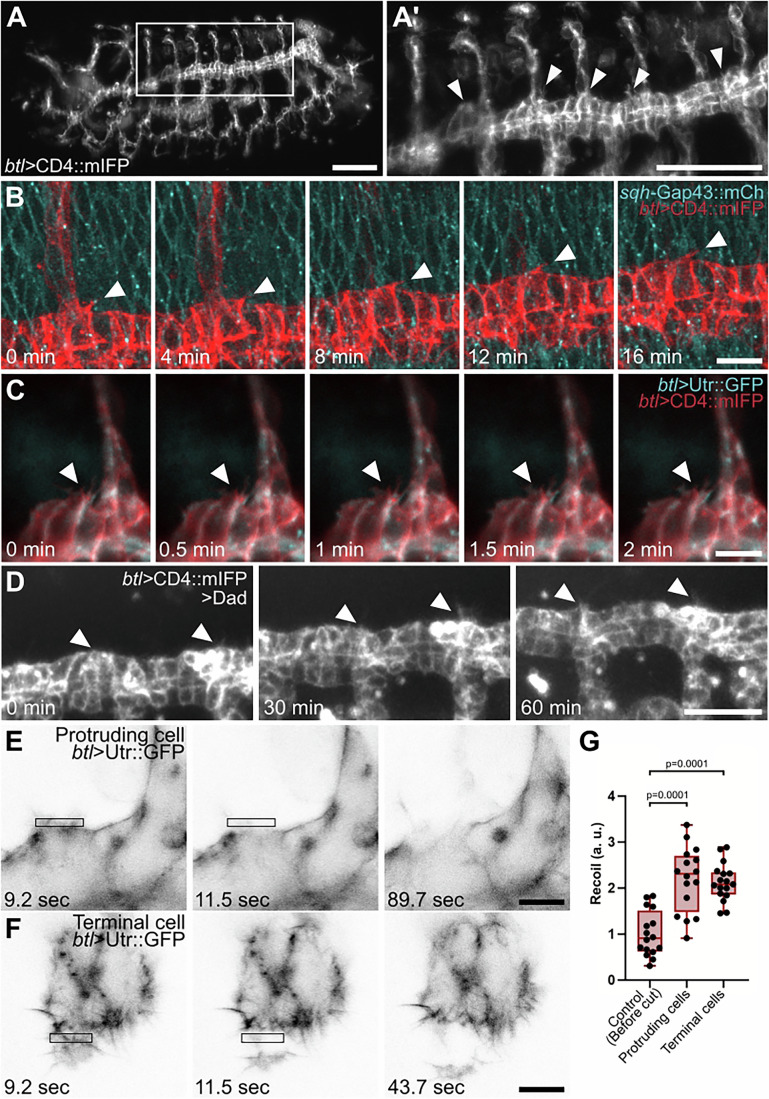
Live imaging of protruding cells and laser microdissection experiments. (**A**–**A’**) Time-lapse imaging of an embryo expressing CD4::mIFP under *btl-gal4* and imaged using MuVi-SPIM. (**B**) Time-lapse imaging of an embryo expressing CD4::mIFP under *btl-gal4* (red) and Gap43::mCherry under direct control of the *sqh* promoter (cyan) and imaged using confocal microscopy. (**C**) Time-lapse imaging of an embryo expressing CD4::mIFP (red) and Utr::GFP (cyan) under *btl-gal4*. (**D**) Time-lapse imaging of an embryo expressing CD4::mIFP and Dad. Arrowheads in (**A**–**D**) point to protruding cells of the dorsal trunks. (**E**) Laser cut in the protruding cells of the dorsal trunk expressing *btl*>Utr::GFP. (**F**) Laser cuts in the terminal cells of the dorsal branch expressing *btl*>Utr::GFP. Laser cuts were done in the regions marked with black squares in (**E**, **F**). (**G**) Quantification of the recoil using PIV. Control, *n* = 16 from 12 embryos; Protruding cells, *n* = 16 from 12 embryos; Terminal cells, *n* = 17 cells from 10 embryos. A box plot represents the median, the interquartile range (IQR) and min and max values. Significance was determined using ANOVA and Dunnett correction for multiple comparisons. (**A**) Scale bar: 50 µm. (**B**) Scale bar: 10 µm. (**C**) Scale bar: 5 µm. (**D**) Scale bar: 20 µm. (**E**, **F**) Scale bars: 5 µm. Source data are available online for this figure.

Electron micrographs also revealed a high filopodial abundance at the interface between tendon cells and tracheal trunks (Figs. 2D and EV2B). Muscles extend filopodia as they attach to tendon cells (Maartens and Brown, 2015), leading us to examine whether tracheal cells could also form filopodia to contact tendon cells. Time-lapse imaging using MuVi-SPIM suggested protrusive activity at tracheal trunks during the dorsal closure stages (Fig. 3A; Movie EV3). We performed high-resolution live imaging of dorsal trunk cells to better visualise this protrusive behaviour. For this, we used *btl* > CD4::mIFP to label the tracheal system and a ubiquitously expressed membrane marker, *sqh*-Gap43::mCherry, to label the epidermis. In time-lapse movies, we observed that trunk cells form protrusions in the direction of the epidermis (Fig. 3B; Movie EV4). These protrusions are very stable; they persist in high time-resolution acquisitions and throughout epidermal dorsal closure (Fig. 3C; Movie EV5). Notably, the cells that form protrusions are evenly distributed across the trunks, at the intersection with muscle attachment sites (Fig. 3A,B; Movie EV3). Also, they are still present in conditions of Dad overexpression, where dorsal branches are not formed (Fig. 3D; Movie EV6). We propose that this subset of tracheal cells mediates the interaction between the trunks and tendon cells and, because of their morphology, we refer to them as “protruding cells”.

If protruding cells adhere to the ECM of muscle attachment sites, we hypothesise that they should be under tension. We tested this by performing laser microdissection directly over these structures and measuring the resulting recoil speed using PIV analyses. We found that seconds after the laser cut was performed, there was a significant recoil in the direction of the trunk (Fig. 3E; Movie EV7), which was comparable to the recoil seen upon laser microdissection of tracheal tip cells (Fig. 3F,G; Movie EV7; (Ríos-Barrera and Leptin, 2022)). These results show that protruding cells are under tension, likely due to their role as mediators of tracheal–epidermal interactions.

### Integrin-ECM adhesion complexes participate in the interaction between dorsal trunks and the epidermis

**Figure 4:**
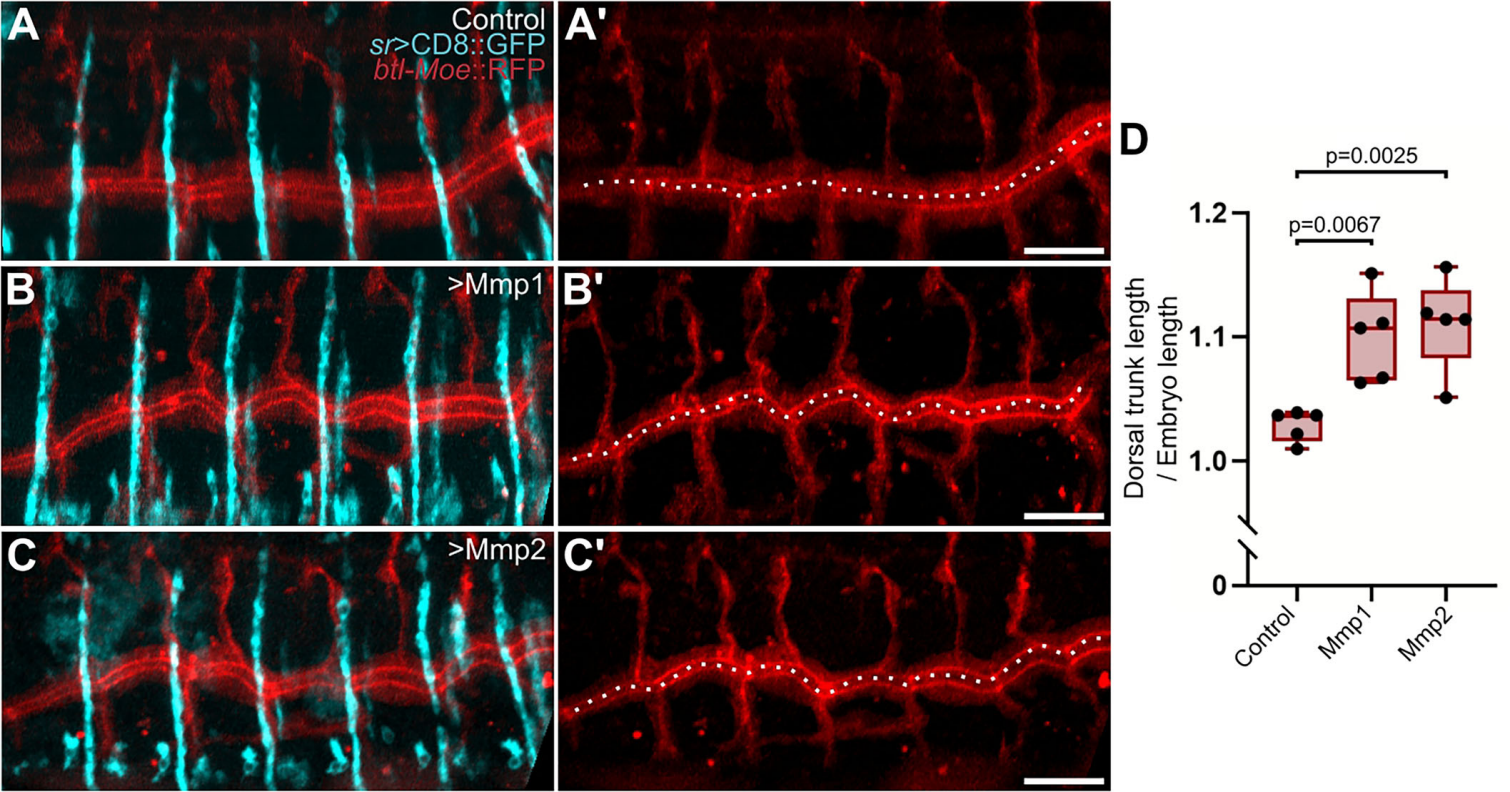
Effect of overexpressing MMPs in tendon cells on tracheal trunk morphology. (**A**–**C**) Live-imaging of embryos expressing CD8::GFP under the tendon cell driver *sr-gal4* (cyan) and *btl-*Moe::RFP (tracheal system, red). (**A**) Control; (**B**) embryo additionally expressing Mmp1; (**C**) embryo additionally expressing Mmp2. (**D**) Normalised dorsal trunk (DT) length. Box plot represents median, IQR and min and max values. Control, *n* = 5; Mmp1, *n* = 5; Mmp2, *n* = 5. Significance was determined using ANOVA and Dunnett correction for multiple comparisons. Scale bars: 30 µm. Source data are available online for this figure.

To test the relevance of the adhesion between protruding cells and muscle attachment sites, we combined *btl-*Moe::RFP to follow the tracheal system together with a tendon cell driver (*stripe; sr-gal4*) that allowed us to visualise these cells and carry out ECM perturbation experiments. We overexpressed matrix metalloproteinases (Mmp1 and Mmp2), enzymes that degrade ECM components (Jia et al, 2014; Llano et al, 2002), and observed their effect on tracheal development. In control embryos, we observed evenly distributed stripes of tendon cells across the anteroposterior axis of the embryo that traversed the tracheal trunks perpendicularly (Fig. 4A–A’). In embryos where we overexpressed Mmp1 or Mmp2 in tendon cells, the general organisation of tendon cells remained the same and tracheal trunks were still displaced dorsally; however, they developed tortuosities over time, a morphology that is not seen in control embryos of similar developmental stages (Fig. 4B–C). Consequently, experimental embryos showed significantly longer dorsal trunks compared with controls (Fig. 4D). As Mmp2 is a membrane-anchored protein (Llano et al, 2002), its protease activity is limited to the ECM adjacent to the tendon cells, reducing the possibility that Mmp2 expressed by the tendon cells could have a direct effect on tracheal trunks by diffusing through the extracellular space.

**Figure EV3:**
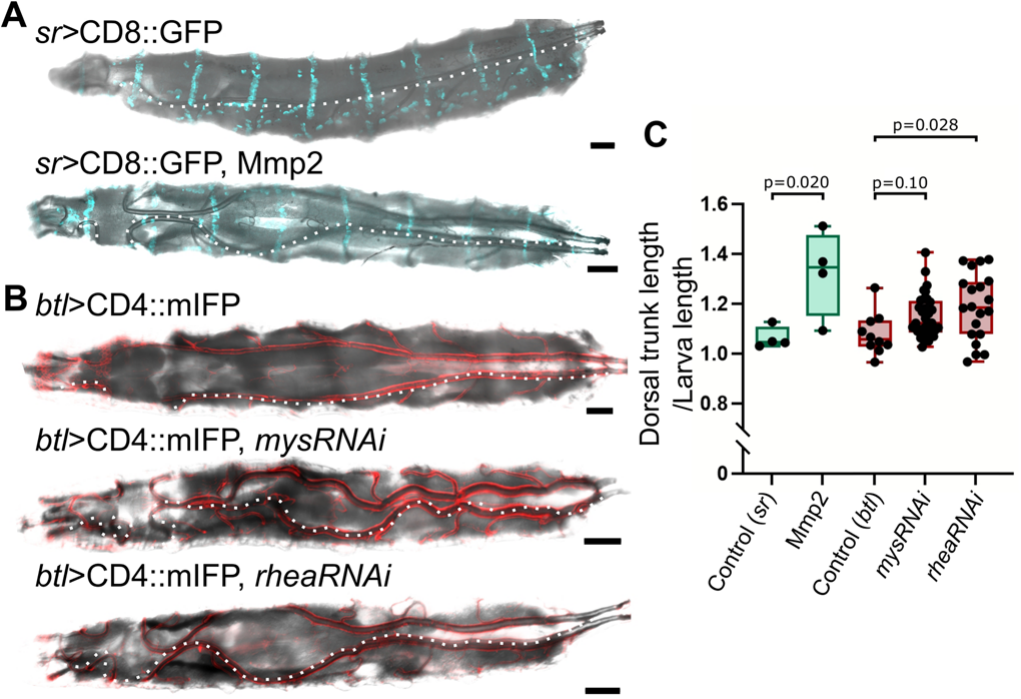
Role of adhesion complexes and ECM in larval dorsal trunk morphology. (**A**–**B**) Overlay of maximum-intensity projections of fluorescent reporters and minimum-intensity projections of reflected light images of heat-fixed third instar larvae. (**A**) Larvae expressing GFP under *sr-gal4*. Top panel, control; bottom panel, expression of Mmp2. (**B**) Larvae expressing CD4::mIFP under *btl-gal4*. Top panel, control; middle panel, *mys* (which codes for β-integrin) RNAi; bottom panel, *rhea* (which codes for Talin) RNAi. (**C**) Normalised dorsal trunk (DT) length. Box plot represents median, IQR and min and max values. Control (*sr*), *n* = 4 larvae; Mmp2, *n* = 4 larvae; Control (*btl*), *n* = 10 larvae; *mysRNAi*, *n* = 33 larvae; *rheaRNAi*, *n* = 20 larvae. Significance was determined using ANOVA and the Kruskal–Wallis test for multiple comparisons. Scale bars: 200 µm. Source data are available online for this figure

We wondered if the tracheal defects caused by Mmp2 expression in tendon cells would persist into later stages of development, so we looked at tracheal trunks in third instar larvae. We found a phenotype like the one we observed in embryos, with dorsal trunks acquiring a wavy appearance, resulting in significantly longer trunks compared with controls (Fig. EV3A). These results show that perturbing the ECM that lies below the tendon cells influences tracheal trunk morphology.

**Figure EV4:**
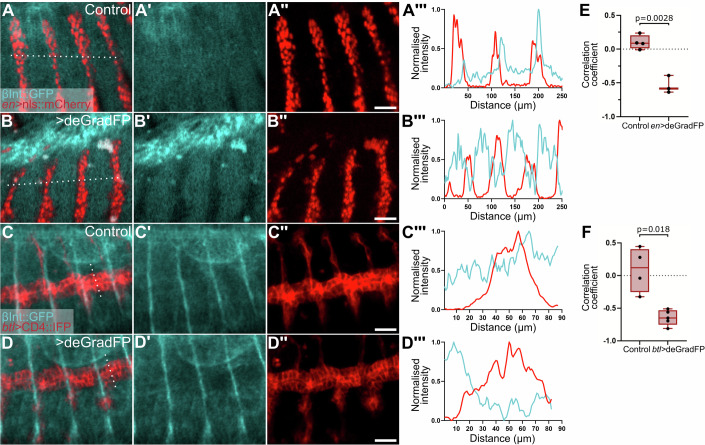
Effect of deGradFP expression on β-Integrin::GFP fluorescence intensity. (**A**–**D**) Maximum intensity projections of embryos expressing β-Integrin::GFP (cyan). (**A**–**B**) Embryos additionally expressing nls::mCherry (red) under *en-gal4*. (**C**–**D**) Maximum intensity projections of embryos expressing CD4::mIFP under *btl-gal4* (red) and β-Integrin::GFP (cyan). (**A**, **C**) Controls; (**B**, **D**) deGradFP expression. (**A”’**–**D”’**) Fluorescence intensity profiles across the dotted white lines drawn in (**A**–**D**). (**E**, **F**) Correlation analyses of fluorescence intensity profiles comparing signals from *en>*deGradFP vs controls (**E**; Control, *n* = 4; *en*>deGradFP, *n* = 3) and *btl>*deGradFP vs controls (**F**; Control, *n* = 4; *btl*>deGradFP, *n* = 5). Box plots represent median, IQR and min and max values. Significance was determined using *t* tests. Scale bars: 20 µm. Source data are available online for this figure

As a complementary approach, we also removed β-Integrin specifically in the tracheal system. Studies on integrins in the embryonic tracheal system have been hampered by an abundant maternal contribution that may obscure early phenotypes and/or by the early lethality of germline clones for mutations in components of the integrin complex (Brown, 1994; Wieschaus and Noell, 1986). Therefore, we used the deGradFP system, which targets GFP-labelled proteins for proteasomal degradation. This is achieved by expressing UAS-NsImb::Vhh4, a modified ubiquitin ligase (NsImb) fused to a nanobody against GFP (Vhh4) that can be expressed under any particular driver (Caussinus et al, 2011). For these experiments, we targeted an endogenous insertion of GFP in the gene *mys*, which codes for β-Integrin (Klapholz et al, 2015). β-Integrin::GFP also allowed us to visualise the epidermis and other tissues in time-lapse experiments. Since β-Integrin::GFP is a transmembrane protein, it was possible that it might be insensitive to proteasomal degradation using deGradFP. We tested the capacity of the system to degrade β-Integrin::GFP with *en-gal4* in stage 12–13 embryos and found that the β-Integrin::GFP signal significantly decreased in the regions where *en-gal4* was expressed (Fig. EV4A–B,E). Testing this using *btl-gal4* was technically more challenging, since by the time *btl-gal4* is active, the β-Integrin::GFP signal is more abundant in other tissues. Nevertheless, we also found a significant decrease in the GFP intensity (Fig. EV4C,D,F).

**Figure 5:**
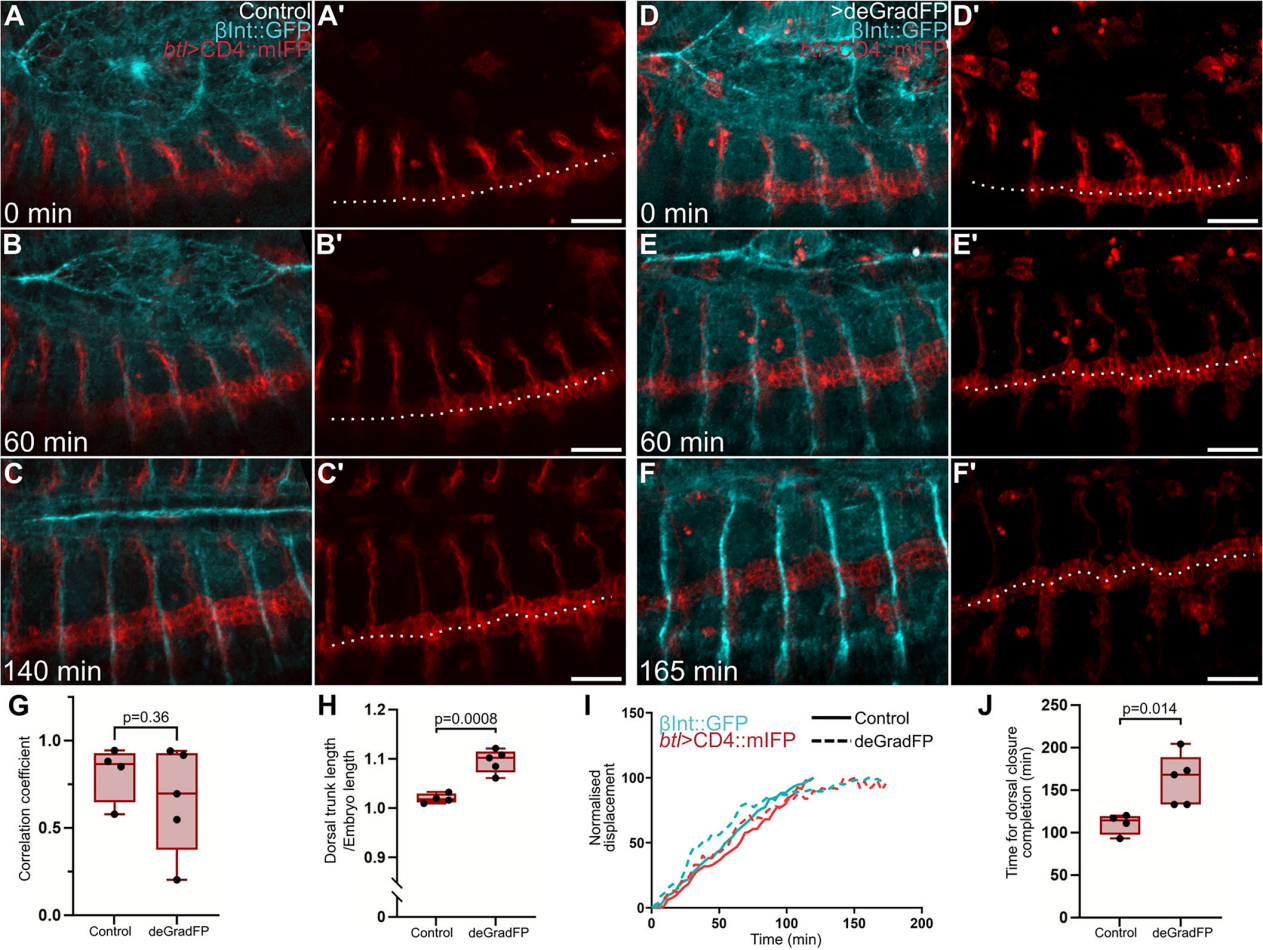
Effect of deGradFP expression on tracheal morphology of embryos expressing β-Integrin::GFP. (**A**–**F**) Maximum intensity projections and time-lapse imaging of embryos expressing β-integrin::GFP (cyan), and CD4::mIFP under *btl-gal4* (red). (**A**–**C**) Control; (**D**–**F**) Embryo additionally expressing deGradFP. Dotted lines in (**A’**–**F’**) highlight the tracheal trunk lumen. (**G**) Quantification of epidermal–dorsal trunk coordination using PIV and cross-correlation analyses. (**H**) Normalised dorsal trunk (DT) length. (**I**) Normalised displacement of epidermis (cyan) and dorsal trunk (red). (**J**) Time for dorsal closure completion. Control, *n* = 4; deGradFP, *n* = 5. Significance was assessed using *t* tests. Box plots in (**G**, **H**, **J**) represent median, IQR and min and max values. Scale bars: 30 µm. Source data are available online for this figure.

When performing time-lapse microscopy, in control embryos, we observed that tracheal trunks move to the dorsal side of the embryo in coordination with the epidermis, as determined by cross-correlation analyses (Fig. 5A–C,G; Movie EV8). When we degraded β-Integrin::GFP in the tracheal system, as in our MMP experiments, we observed coordinated tissue displacement (Fig. 5D–G; Movie EV8) and tracheal trunks also showed a wavy lumen phenotype, resulting in significantly longer trunks (Fig. 5H). We corroborated these results in third instar larvae using RNAi against *mys* and *rhea* (which code for β-Integrin and its intracellular mediator Talin, respectively). As with Mmp2 overexpression using *sr-gal4*, silencing these genes under *btl-gal4* resulted in wavy dorsal trunks (Fig. EV3B–C). These manipulations led to partial lethality (32% of expected animals hatched as adults, *n* = 61), suggesting partial silencing or backup mechanisms that suppress lethality. Nevertheless, these results show that cell–ECM interactions are important for proper trunk morphology during embryonic and larval development.

### Affecting dorsal closure results in aberrant tracheal morphogenesis and vice versa

In our deGradFP experiments, we noticed that in addition to the effects on tracheal trunks, epidermal dorsal closure also appeared to be affected upon depletion of β-Integrin::GFP from the tracheal system (Fig. 5F; Movie EV8). We quantified the time required for dorsal closure completion in these embryos, and we found a significant delay compared with controls (Fig. 5I–J). Albeit unexpected, these results suggested an interdependence between epidermal and tracheal development. We decided to test the opposite scenario, where we affected dorsal closure and observed the effect on tracheal development. To do this, we used mutant embryos where epidermal dorsal closure was affected. Mutations in genes that code for components of the JNK signalling pathway either lead to a complete failure of or to a delay in dorsal closure. We used *kay*^*1*^ (which codes for Fos) mutant embryos and focused on embryos with a delayed dorsal closure phenotype to see if this would have an effect on tracheal morphogenesis. We employed *btl* > CD4::mIFP to visualise the tracheal system, together with E-Cad fused to GFP to follow the progression of epidermal dorsal closure.

**Figure 6:**
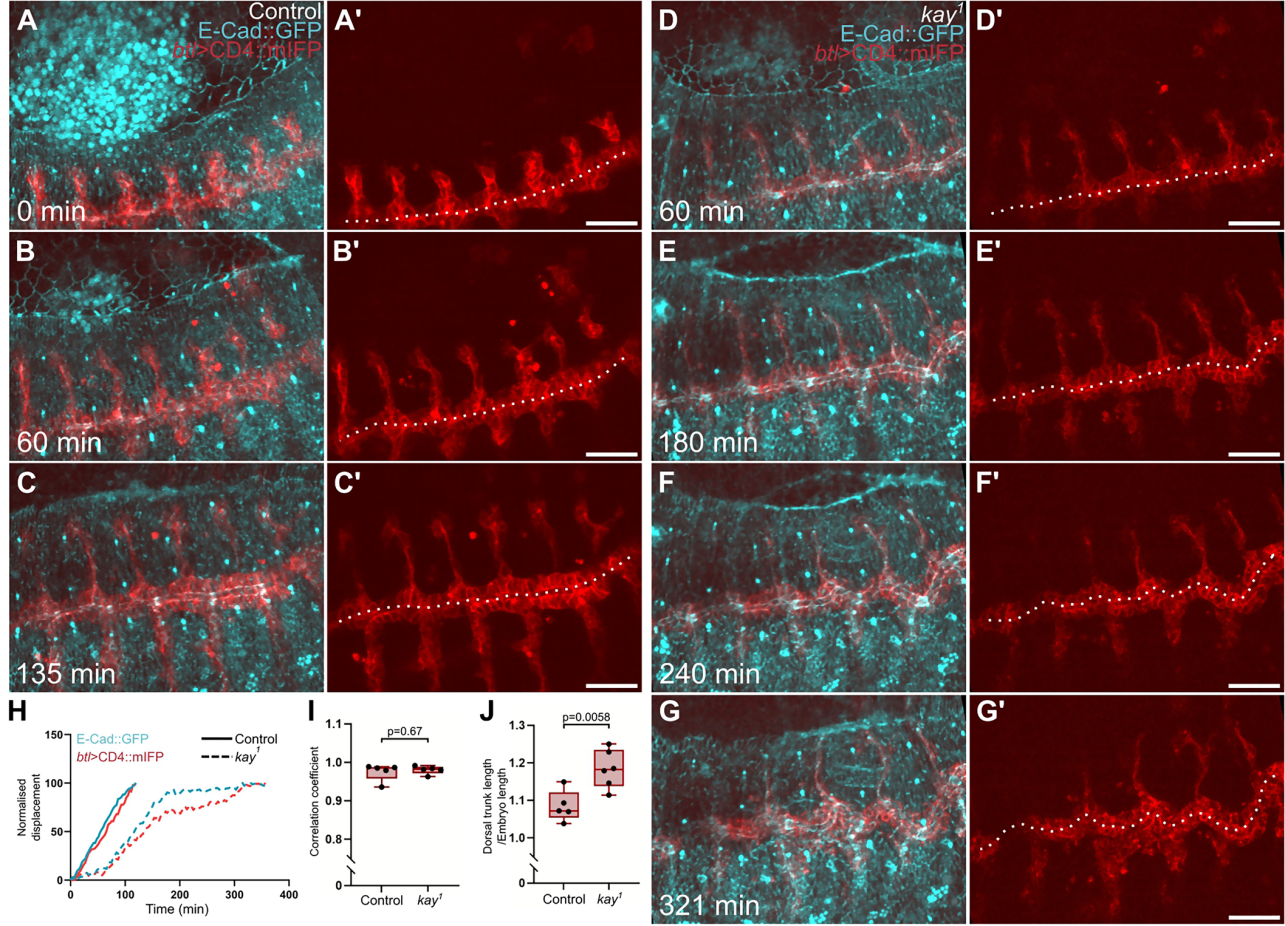
Tracheal trunk morphology in *kay*^*1*^ mutant embryos with a delayed dorsal closure phenotype. (**A**–**G**) Maximum intensity projections and time-lapse imaging of embryos expressing E-Cad::GFP (cyan), and CD4::mIFP under *btl-gal4* (red). (**A**–**C**) Control embryo; (**D**–**G**) *kay*^*1*^ mutant embryo. (**H**) Normalised displacement of the epidermis (cyan) and dorsal trunk (red). (**I**) Quantification of epidermis-dorsal trunk coordination. Control, *n* = 5; *kay*^*1*^, *n* = 5. (**J**) Normalised dorsal trunk (DT) length. Control, *n* = 5; *kay*^*1*^, *n* = 6. Significance was assessed using *t* tests. Box plots in (**I**–**J**) represent median, IQR and max and min values. Scale bars: 30 µm. Source data are available online for this figure.

As in control embryos, where we observed that dorsal closure progressed in parallel to trunk displacement (Fig. 6A–C,H,I; Movie EV9), in *kay*^*1*^ mutant embryos with a delayed closure phenotype, tracheal trunk displacement still occurred at the same rate as epidermal dorsal closure (Fig. 6D–I; Movie EV9). However, in *kay*^*1*^ mutant embryos, tracheal trunks developed malformations that were not observed in controls, which consisted of tortuosities that changed in shape over time and mimicked movements also observed in the epidermis. Again, in this mutant condition, tracheal trunks were longer than those in controls (Fig. 6J).

**Figure EV5:**
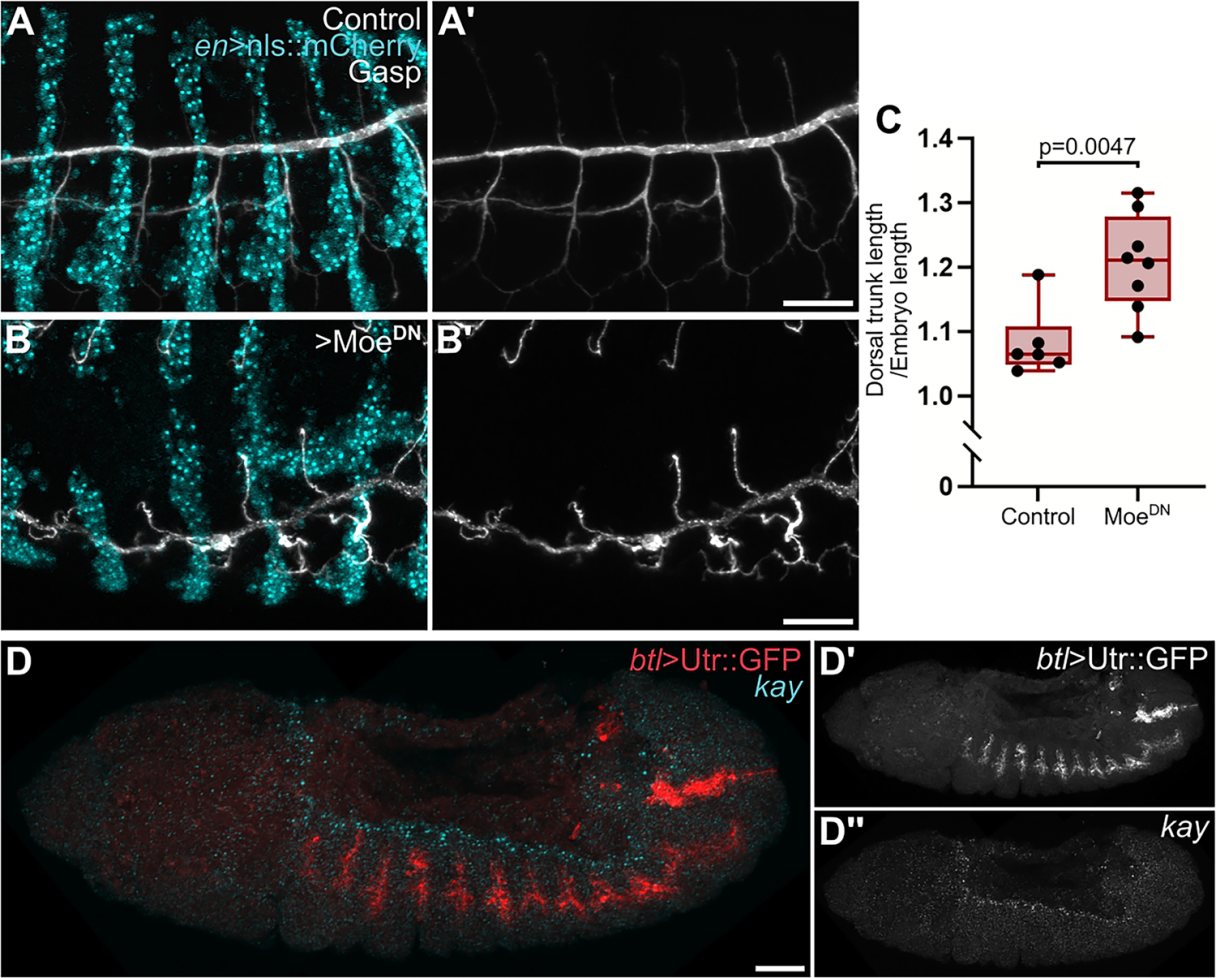
Role of the dorsal closure progression in tracheal trunk morphology. (**A**–**B**) Maximum intensity projections of embryos expressing nls::mCherry under *en-gal4* and immunostained for Gasp. (**A**) Control (**B**) Embryo with additional expression of Moe^DN^. (**C**) Quantification of normalised dorsal trunk length. Control, *n* = 6 embryos; Moe^DN^, *n* = 8 embryos). Significance was assessed using *t* test. Box plot represents mean, IQR and min and max value. (**D**–**D”**) HCR-FISH against *kay* (cyan) in embryos with expression of Utr::GFP under *btl-gal4* (red). Scale bars: 30 µm. Source data are available online for this figure

The defects we observed in *kay* mutant embryos could be explained by a requirement for Fos in the tracheal system; however, we performed HCR-FISH against *kay* in control embryos and, as reported with earlier methods (Riesgo-Escovar and Hafen, 1997; Souid and Yanicostas, 2003), we detected a strong signal at the epidermal leading edge and only a faint generalised signal in the rest of the embryo (Fig. EV5D–D”). To corroborate that the defects we saw on the tracheal system are due to a delayed closure, we overexpressed a dominant-negative form of Moesin in stripes of the epidermis using *en-gal4*. In these embryos, we stained for Gasp, a tracheal luminal marker, and again we found the wavy trunk phenotype (Fig. EV5A–C). These experiments show that the tracheal–epidermal association is present even in conditions of delayed dorsal closure. Furthermore, they support the idea that proper trunk development depends on timely epidermal dorsal closure and that delaying this process also delays trunk displacement.

### In the absence of epidermis attachment, removing dorsal branches rescues trunk morphology

In all our manipulations, we found that trunks acquire a wavy phenotype regardless of whether we affect tracheal–epidermal interactions or delay epidermal movements. In these two approaches, we propose that the wavy trunk phenotype may arise from different mechanisms. Upon completion of dorsal closure and as head involution takes place, the epidermis and the tracheal system elongate towards the anterior part of the embryo. We think that in the experiments where we delay dorsal closure, the trunks grow along the anteroposterior axis while the epidermis is still moving dorsally. Since in these embryos the trunks are still attached to the epidermis, the wavy phenotype could emerge because of the mismatch in the direction of growth between the epidermis and the trunks. In fact, in *kay* mutant embryos, we observe that after completion of dorsal closure, the wavy trunk phenotype slightly resolves once the epidermis moves anteriorly (Movie EV9).

**Figure 7:**
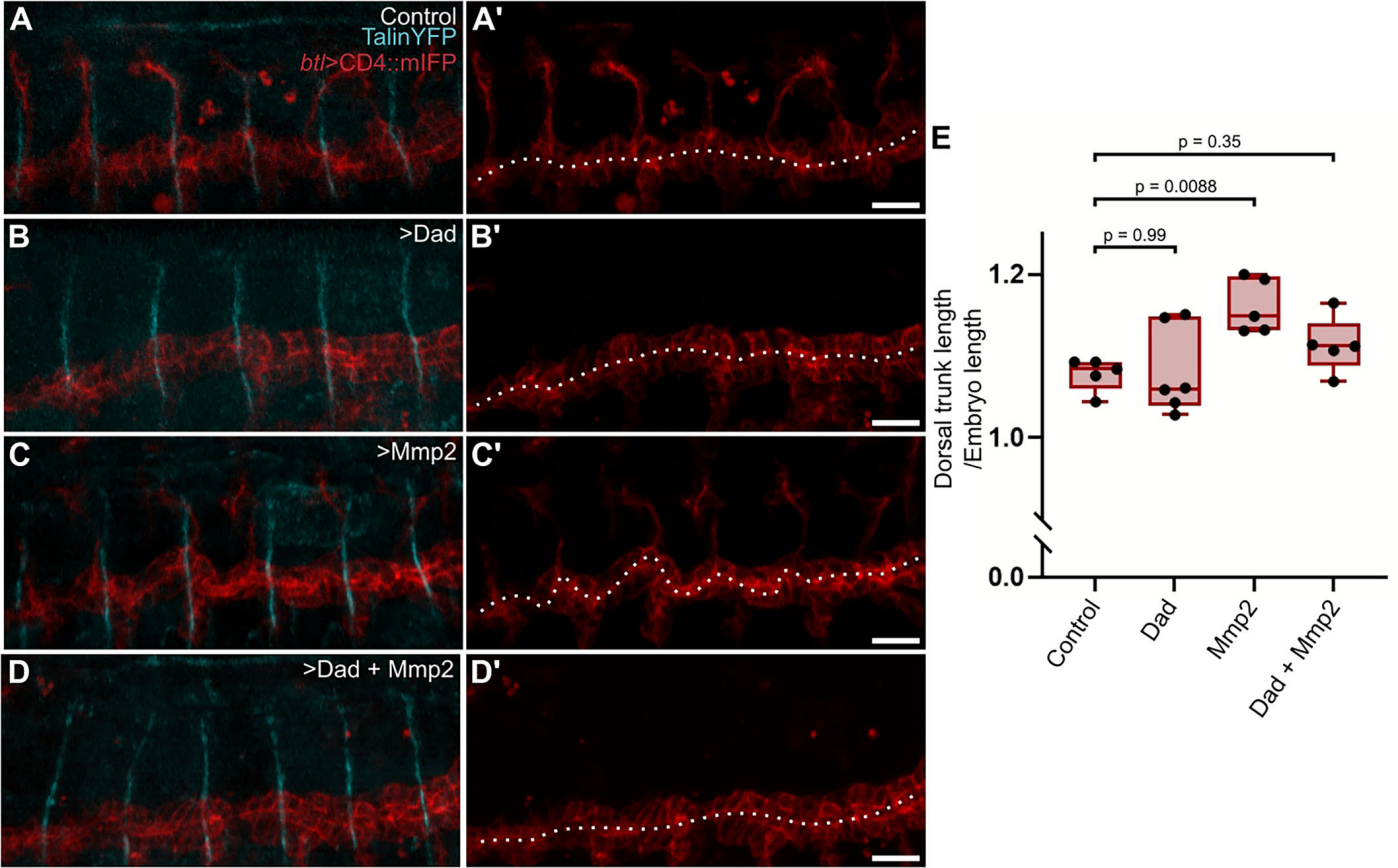
Role of the dorsal branches in the tracheal trunk malformations. (**A**–**D**) Maximum intensity projections of embryos expressing Talin::YPet (cyan), and CD4::IFP under *btl>gal4* (red). (**A**) Control. (**B**) Embryo additionally expressing Dad. (**C**) Embryo additionally expressing Mmp2. (**D**) Embryo additionally expressing Dad and Mmp2. (**E**) Normalised dorsal trunk length. Control, *n* = 5; Dad, *n* = 6; Mmp2, *n* = 5; Dad + Mmp2, *n* = 5. Box plot represents median, IQR and min and max value. Significance was determined using ANOVA and Dunnett correction for multiple comparisons. Scale bars: 20 µm. Source data are available online for this figure.

In embryos where we overexpress MMPs or knock down β-Integrin, we reasoned that in the absence of, or with weakened contact points with the epidermis, dorsal branches could exert forces on the trunks and pull them dorsally, resulting in the wavy phenotype. We tested this by simultaneously overexpressing Mmp2 and Dad in the tracheal system. Unlike controls or Dad-expressing embryos (Fig. 7A,B), Mmp2 overexpression induced a wavy trunk phenotype (Fig. 7C) similar to that observed upon Mmp2 overexpression in tendon cells; however, co-expression of Dad rescued this phenotype (Fig. 7D,E). This supports the conclusion that dorsal branches can pull on the trunks as they migrate dorsally and that weakening adhesion to the epidermis leads to an uneven repositioning of the trunks.

Altogether, we conclude that the adhesion of the dorsal trunks to the tendon cells of the epidermis through the ECM is important for trunk repositioning. In addition, this interaction plays a stabilising role in maintaining the straightness of the dorsal trunks during their movement to the dorsal side of the embryo. These interactions are important for tracheal morphogenesis, but they also influence epidermal dorsal closure.

## Discussion

**Figure 8:**
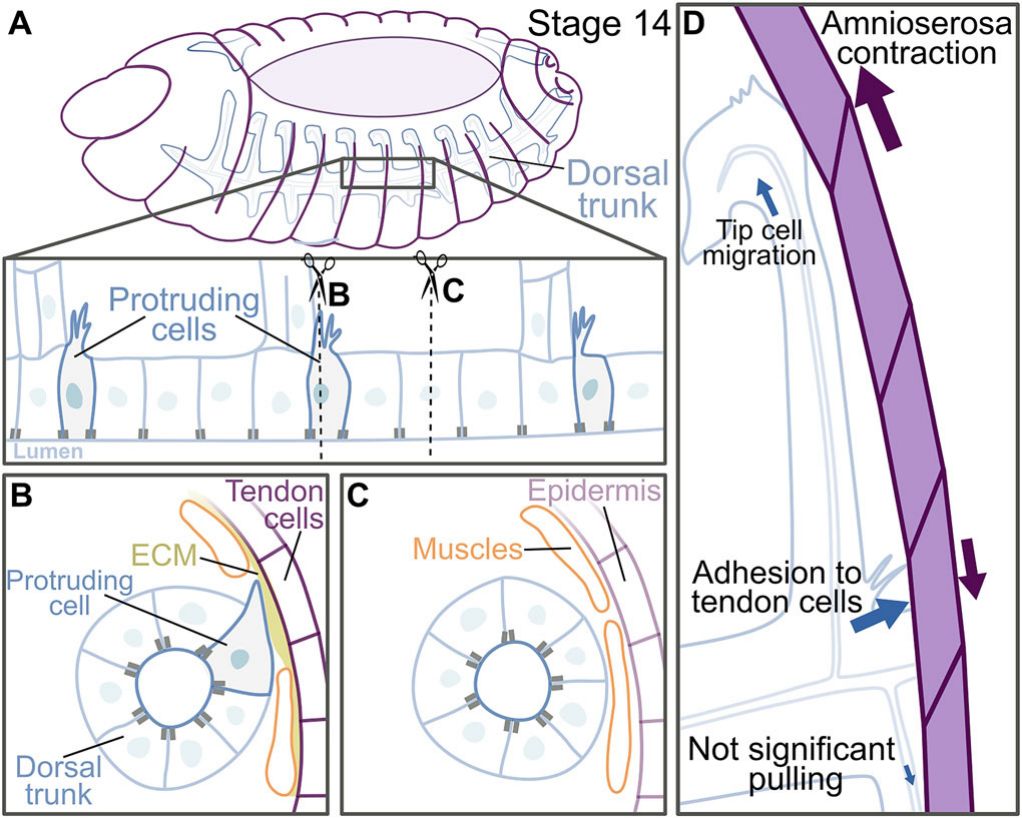
Model of trachea–epidermis interactions. (**A**) Overview of a stage 14 embryo at the onset of dorsal closure. The black square at the dorsal trunk is magnified below, showing the distribution of protruding cells within the dorsal trunk. The dotted lines indicate the planes for the cross-sections presented in (B and C). (**B**) Just below tendon cells, protruding cells extend filopodia towards the basement membrane of the tendon cells. (**C**) Regions of the trunks not near tendon cells are separated from the epidermis by muscles and other cells. (**D**) Summary of forces acting during dorsal trunk displacement. The interactions between the protruding and tendon cells allow trunk repositioning at the same time as they counteract the pulling forces produced by migrating tip cells. In addition, during this process, the dorsal trunk acts as a counterweight for the epidermis, balancing epidermal dorsal closure progression. Ventral branch extension does not influence dorsal trunk displacement.

Here, we demonstrated that epidermal closure and tracheal trunk displacement are coordinated events, with evidence suggesting that ECM integrity plays a key role in their morphogenesis. We identified a population of tracheal cells, termed ‘protruding cells’, which mediate the interaction with the epidermis (Fig. 8A) by adhering to the ECM underlying the epidermal tendon cells (Fig. 8B,C). In this model, the ECM acts simultaneously as a milieu for the diffusion of extracellular signals and as a scaffold that transmits forces, linking tissues as they undergo complex rearrangements. In the case of tracheal–epidermal interactions, a number of classical studies have shown that signals produced by the epidermis act within the epidermis itself but also regulate tracheal patterning ((Affolter et al, 1994; Chihara and Hayashi, 2000; Glazer and Shilo, 2001; Kato et al, 2004; Llimargas, 2000), reviewed in (Sánchez-Cisneros et al, 2025)). Our experiments show that, besides these well-characterised signals, mechanical interactions between the two tissues also fine-tune tracheal behaviour. Specifically, we show that pulling forces from dorsal tip cells are dispensable for trunk displacement under normal conditions, though they remain capable of pulling on the trunks when epidermal adhesion is lost. Instead, trunk displacement is facilitated by adhesion to the epidermis—an interaction that, in turn, influences epidermal dorsal closure (summarised in Fig. 8D).

### Emergence of a ‘wavy trunk’ phenotype upon disruption of tracheal–epidermal interactions

Our results demonstrate that perturbing tracheal–epidermal interactions via different mechanisms (either by weakening the ECM through Mmp overexpression, targeting tracheal β-integrin for degradation, or delaying epidermal dorsal closure) consistently results in similar phenotypes: trunks are still displaced dorsally but they acquire a wavy appearance. This suggests that tracheal trunk displacement is in fact dependent on epidermal development. Interactions between trunks and the epidermis likely allow trunk repositioning while preventing deformation by the pulling forces that result from dorsal branch elongation. Previous work focusing on dorsal branch cell development used laser microdissection to show that tip cell migration is responsible for branch elongation and intercalation; this makes it likely that the force generated by tip cells can act on the trunks at the base of dorsal branches (Caussinus et al, 2008).

The wavy trunk phenotype has previously been reported as a result of the loss of laminins (Klußmann-Fricke et al, 2022). Although the phenotype that manifests in laminin (*LanB1*) mutant embryos is stronger than that we report here, we interpret it as being at least partly due to the loss of the interaction with the epidermis. The stronger phenotype described in laminin mutant embryos could be explained by an additive effect of cell-autonomous defects in cell polarity, as previously suggested (Klußmann-Fricke et al, 2022), by the incomplete silencing of integrin-mediated adhesion in our experiments, or by the presence of other adhesion complexes that rely on laminin besides the integrin complex.

### Interactions between protruding cells and tendon cells

According to our model, tracheal–epidermal interactions occur at specific contact points distributed along the anteroposterior axis of the embryo, where tracheal trunks intersect with tendon cells of the epidermis. The protruding cells we describe here are frequently found directly in front of tracheal dorsal branches; we believe this has prevented their characterisation, as they are difficult to visualise with general tracheal reporters.

Tendon cells are well-known force transmitters, as they interact with the exoskeleton and muscle cells through apical and basal ECM layers, respectively, enabling locomotion as muscles contract (Chu and Hayashi, 2021; Martin-Bermudo, 2000; Weitkunat et al, 2014). Our analyses suggest that these points also mediate the adhesion of tracheal trunk cells–the protruding cells–to transmit forces relevant to tracheal remodelling. As mentioned earlier, in the face of pulling forces exerted by dorsal tip cell migration, adhesion to the tendons may counteract trunk deformation. Through this interaction, stalk cells can intercalate while trunks remain in place. (Caussinus et al, 2008; Ochoa-Espinosa et al, 2017). We do not observe defects in cell intercalation in the conditions we tested, but these could be compensated for by the looping of the trunks upon pulling interactions from tracheal branches.

Our results do not address whether protruding cells possess different gene expression profiles compared with their neighbours, or if they acquire a distinctive morphology due to their proximity to the specialised ECM found near tendon cells. Previous studies have shown that tendon cells secrete FGF, which in turn regulates tracheal development (Butí et al, 2014; Dorfman et al, 2002). However, it is unlikely that tendon-cell-derived FGF is responsible for the formation of these protrusions, as tracheal trunk cells do not activate landmarks of FGF signalling, such as SRF expression or ERK phosphorylation (Gervais and Casanova, 2011). Previous studies have induced tendon cell differentiation by overexpressing Stripe in other epidermal cells (Dorfman et al, 2002). Because of the resulting effect on FGF expression, it is difficult to assess the effect on trunk cell behaviour from these experiments.

### Tracheal–epidermal interactions and their role in epidermal dorsal closure

Besides the effects on the trunks, we found that targeting β-integrin in the tracheal system also resulted in a delay in epidermal dorsal closure. Previously, it was shown that dorsal tip cell migration is independent of epidermis displacement (Caussinus et al, 2008). Our results do not contradict these experiments; we observe adhesion only at the level of the tracheal trunks, and from our experiments, there are no effects on tip cell morphology or migration. It has been shown that the forces produced by the amnioserosa are the main components that allow epidermal dorsal closure; even when the epidermis forms a supracellular actomyosin ring at the dorsal-most row of epidermal cells, this only controls the seamless sealing of the epidermal sheets at the dorsal midline (Pasakarnis et al, 2016). It was suggested recently that the epidermis itself acts as a counter-force in response to the pulling forces of the amnioserosa (Lv et al, 2022). Our results support this view and suggest that the adhesion of the trunks to the epidermis may contribute to balancing the interplay of forces that participate in epidermal dorsal closure. As we removed a force that opposes epidermal stretching, we did not anticipate a delayed dorsal closure. However, the delay could be explained by disorganised epidermal movements. In accordance with this, mutants for genes that negatively regulate dorsal closure progression also affect the proper sealing of the epidermis (Jacinto et al, 2002; Martín-Blanco et al, 1998; Ríos-Barrera et al, 2015).

### The ECM as a coordinator of morphogenesis

Mechanical interactions between adjacent tissues and how they shape morphogenesis have been described in a wide range of processes. These range from the above-mentioned epidermal-amnioserosa interactions in *Drosophila* development (Fernández et al, 2007; Frank and Rushlow, 1996; Hayes and Solon, 2017; Kiehart et al, 2017) to epidermal-blood vessel interactions in the rhinarium of dogs and other species (Dagenais et al, 2024). In many of these processes, the ECM, its mechanical properties, and its remodelling play important roles in force transmission and/or coordinating tissue behaviour [(Goodwin et al, 2016; Goodwin et al, 2017; Inoue and Hayashi, 2007; Münster et al, 2019), reviewed in (Barrera-Velázquez and Ríos-Barrera, 2021)]. We suggest that ECM-mediated tissue adhesion may have a role in general embryo development, integrating morphogenetic processes of seemingly unrelated tissues.

## Methods

**Table:** Reagents and tools table

Reagent/resource	Reference or source	Identifier or catalogue number
**Experimental models**		
*btl-gal4, UAS-*mCD4::mIFP	Mathew et al, 2020	NA
*btl-gal4, UAS-*PH::mCherry	Mathew et al, 2020	NA
*btl-gal4, UAS-*Utrophin::GFP	Ríos-Barrera and Leptin, 2022	NA
*Collagen-IV::GFP*	Ríos-Barrera et al, 2017	NA
*en-gal4, UAS*-GFP	Tobias Troost, Heinrich Heine University Düsseldorf	NA
*sqh-*Gap43::mCherry	Thomas Lecuit lab, IBDM, France (Rauzi et al, 2010);	NA
*kay* ^*1*^	Juan Riesgo-Escovar, UNAM, Mexico (Riesgo-Escovar and Hafen, 1997)	NA
Mys::GFP	Nick Brown, MRC, UK (Klapholz et al, 2015)	NA
Talin::YPet	Frank Schnorrer, IBDM, France (Spletter et al, 2018)	NA
*UAS-*CD8::GFP	Fanis Missirlis, CINVESTAV, Mexico	NA
*sr-gal4*	Bloomington Drosophila Stock Center (BDSC)	#26663
*UAS-mys* TRiP	BDSC	#27735
*en-gal4, UAS-nls::mCherry*	BDSC	#38420
*UAS-NsImb-vhhGFP4*	BDSC	#38421
*UAS-Mmp1.f2*	BDSC	#58702
*UAS-Mmp2*	BDSC	#58705, #58706
E-Cadherin::GFP	BDSC	#60584
*UAS-Moe.myc.T559A* (*Moe*^*DN*^)	BDSC	#52234
*btl-moeRFP*	BDSC	#64233
*UAS-Dad*	BDSC	#98452
TM6B,dfdYFP	BDSC	#8704
CyO,sChFP; TM3,sChFP	BDSC	#92597
*UAS-Talin* RNAi	Vienna Drosophila Stock Center	#40399
**Antibodies**		
Anti-GFP coupled to FITC, 1:500	Abcam	#ab6662; RRID:AB_305635
Anti-βIntegrin, 1:20	Developmental Studies Hybridoma Bank (DSHB)	#CF.6G11; RRID:AB_528310
Anti-Gasp, 1:20	DSHB	#2A12; RRID:AB_528492
Goat anti-mouse IgG coupled to Alexa647, 1:300	Thermofisher	#A21235; RRID:AB_2535804
Goat anti-mouse IgM coupled to Alexa647, 1:300	Thermofisher	#A21238; RRID:AB_2535807

### Fly lines

Fly stocks were kept under standard culture conditions. Experiments were carried out at 25 °C or 29 °C where stated. *btl-gal4, UAS-*mCD4::mIFP*; btl-gal4, UAS-*PH::mCherry*; btl-gal4, UAS-*Utrophin::GFP, and *Collagen-IV::GFP* have been described previously and were provided by Maria Leptin’s lab at EMBL, Heidelberg (Ríos-Barrera and Leptin, 2022; Ríos-Barrera et al, 2017). *en-gal4, UAS*-GFP was provided by Tobias Troost, Heinrich Heine University Düsseldorf, Germany; *sqh-*Gap43::mCherry is from the Thomas Lecuit lab, IBDM, France (Rauzi et al, 2010); and *kay*^*1*^ was obtained from Juan Riesgo-Escovar, UNAM, Mexico (Riesgo-Escovar and Hafen, 1997). Insertion of GFP at the *mys* locus (β-Integrin::GFP) was a kind gift from Nick Brown, MRC, UK (Klapholz et al, 2015), and the insertion of the YFP derivative YPet at the *rhea* locus (Talin::YPet) was provided by Frank Schnorrer, IBDM, France (Spletter et al, 2018). *UAS-*CD8::GFP was obtained from Fanis Missirlis, CINVESTAV, Mexico. The following lines were obtained from the Bloomington Drosophila Stock Center: *sr-gal4* (#26663); *UAS-mys* TRiP (#27735); *en-gal4, UAS-nls::mCherry* (#38420); *UAS-NsImb-vhhGFP4* (#38421); *UAS-Mmp1.f2* (#58702); *UAS-Mmp2* (#58705 and #58706); insertion of GFP at the *shg* locus (E-Cadherin::GFP, #60584); *UAS-Moe.myc.T559A* (*Moe*^*DN*^*;* #52234) *btl-moeRFP* (#64233), and *UAS-Dad* (#98452). To select genotypes of interest, we used balancers with fluorescent reporters from BDSC: TM6B,dfdYFP (#8704), CyO,sChFP and TM3,sChFP (both in #92597) *UAS-Talin* RNAi was obtained from the Vienna Drosophila Resource Center (#40399).

### Immunostainings

Immunostaining was carried out using standard methods. Embryos were dechorionated using 50% commercial bleach for 30 s, then washed with water and transferred to heptane. Afterward, embryos were fixed in 37% formaldehyde for 8 min. For devitellinization, formaldehyde was replaced with methanol, and vials were vigorously shaken for 1 min. We rehydrated the embryos using PBS containing Triton X-100 0.3% and washed them three times. We then blocked using the washing solution and 1% BSA, followed by overnight incubation with the corresponding antibody at 4 °C. The next day, we did three 10 min washes and incubated the embryos with secondary antibodies for 1 h. These were then washed three times for 10 min each, and finally, mounted using Vectashield with DAPI. The antibodies used were: anti-GFP coupled to FITC (Abcam #ab6662; RRID:AB_305635, 1:500); anti-βIntegrin (Developmental Studies Hybridoma Bank [DSHB] #CF.6G11; RRID:AB_528310, 1:20); and anti-Gasp (DSHB #2A12; RRID:AB_528492, 1:20). Secondary antibodies included goat anti-mouse IgG coupled to Alexa647 (Thermo Fisher #A21235; RRID:AB_2535804, 1:300) and goat anti-mouse IgM coupled to Alexa647 (Thermo Fisher #A21238; RRID:AB_2535807, 1:300). Samples were analysed in a Nikon A1R+ confocal microscope using a resonant scanner and a 60×/1.2 NA water objective, and processed using Fiji version 1.54p (Schindelin et al, 2012).

### Live microscopy

Embryos were dechorionated as above, washed with water, and transferred to an agar plate to manually select embryos at the desired stages. For confocal live imaging, embryos were glued to glass-bottom dishes using heptane glue and covered with halocarbon oil. Samples were analysed using a Nikon A1R+ confocal microscope using a resonant scanner and a 60×/1.2 NA water objective, and processed using Fiji (Schindelin et al, 2012). The z-step size was 0.213 μm.

For light-sheet imaging, dechorionated embryos were mounted in low-melting temperature agarose within a glass capillary. The gel cylinder containing the embryo was then pushed above the glass rim to provide optical access. The position of the embryo was then fixed, and the glass capillary was placed inside the PBS-filled imaging chamber of a homebuilt MuVi-SPIM. 3D volumes were acquired as described previously (Caroti et al, 2018; de Medeiros et al, 2015), using two 20×/1.0 NA water immersion detection objectives (Olympus XLUMPLFLN20XW) for fluorescence detection and two 10×/0.3 NA water immersion objectives (Nikon CFI Plan Fluor 10X W) for excitation. Images were processed in Fiji (Schindelin et al, 2012). The time resolution was 2 min per frame, and the z-step was 1.5 μm.

### Laser microdissection

Embryos were processed for live microscopy as above. Laser microdissection experiments were carried out as detailed previously (Sánchez-Cisneros et al, 2023), and as documented in Bio-protocol (10.21769/BioProtoc.4806). We used a Zeiss LSM 780 microscope using a 63x/1.4NA oil objective. Ablation was performed using a femtosecond-pulsed two-photon laser at 950 nm, 75% (1540 mW) power. Microdissection was performed using a single cycle on a single 1 μm thick z-stack.

### Larval fixation

Third-instar larvae were heat-fixed by transferring them to halocarbon oil on a coverslip placed on a heating plate at 65 °C for 15 s. Afterward, the larvae were oriented with the dorsal side downwards. Larvae were analysed using a Nikon A1R+ confocal microscope using a resonant scanner in mosaic mode and a 10x/0.25 NA air objective, and processed using Fiji.

### Electron microscopy

Embryos were processed according to the method of Tepass & Hartenstein (Tepass and Hartenstein, 1994). After dechorionation, the embryos were fixed in a solution of 2% glutaraldehyde and heptane at a ratio of 2:8 for 17 min with agitation. After fixation, we removed the vitelline membranes manually using insulin needles. Embryos were then fixed in 2.5% glutaraldehyde in cacodylate buffer (50 mM, pH 7.2) and post-fixed in a 2% osmium tetroxide solution. Following fixation, the embryos were gradually dehydrated through an ethanol series (70, 80, 90, 95, 100%) and finally acetone. The dehydrated embryos were incubated in acetone/Epon solution overnight and then embedded in Epon resin for 2 days. Afterward, 1-μm semi-thin sections were stained with methylene blue and visualised under a conventional light microscope to identify the regions of interest. Once these were identified, we did ultrathin (50–100 nm) sections and analysed them using transmission electron microscopy. This was carried out using a JEOL JEM 1200 EXII electron microscope. Feature tracing was performed using Inkscape (version 1.3.2).

### Fluorescent in situ hybridisation

We used the Hybridisation Chain Reaction system (HCR RNA-FISH, Molecular Instruments) in an RNAse-free environment. Embryos were fixed in a mixture of heptane and 3.2% paraformaldehyde (PFA) in PBS for 30 min, and then devitellinised by removing PFA, adding methanol, and shaking vigorously for 1 min. Subsequently, embryos were washed with ethanol and incubated overnight in 100% ethanol at 4 °C. The next day, we washed the samples with 0.5% PBS Triton X-100 for 30 min and incubated them in pre-warmed hybridisation buffer at 37 °C for 30 min. We incubated the embryos overnight at 37 °C in a *kay* probe solution (1:250, designed to target all *kay* isoforms and for use with B3 amplifier). Next, we washed the samples four times with pre-warmed probe solution (15 min per wash), followed by two 5-min washes with 5× SSCT at room temperature. Afterwards, we incubated the embryos in amplification buffer for 30 min at room temperature. We snap-cooled hairpins h1 and h2 separately (B3 amplifier labelled with Alexa594) by heating them at 95 °C for 90 s and cooling them to room temperature in the dark before mixing them with amplification buffer. This mixture was then added to the embryos and incubated overnight at room temperature. Finally, the next day, we performed four washes with 5x SSCT at room temperature (5, 5, 30, and 30 min) and mounted the samples using Vectashield with DAPI. Images were acquired in a Nikon A1R+ confocal and a 60×/1.2 NA water objective.

### Image processing, analysis and statistics

We registered all time-lapse micrographs in Fiji using the ‘Correct 3D drift’ function. Cartographical projections of the embryo surface were generated using a custom-made Fiji macro that extracts the curvature of the embryo and reslices over 16 z-planes throughout the time-lapse. To measure tissue coordination, we performed particle image velocimetry (PIV) using Fiji (Tseng et al, 2012), with an interrogation window size of 128×128 px (6.25% of the image) with an overlap of 64 px, and a correlation threshold of 0.60. Cross-correlation analyses were carried-out using a custom-made R script, using the average of magnitudes in the *y* axis generated by the PIV plugin.

Embryonic and larval dorsal trunk length measurements were performed manually in 3D using Imaris (Bitplane) under a trial license; these were normalised to the minimum distance between the initial and final measurement points. Embryonic dorsal trunk measurements were always taken immediately after epidermal dorsal closure was completed. Rates of dorsal closure progression were measured using the Manual Tracking plugin in Fiji, following the signal corresponding to the leading edge of the epidermis and the dorsal edge of the tracheal trunk. In both cases, we followed the same segment corresponding to the centre of the amnioserosa (Abdominal segments 3–4). We plotted displacement in the *y* axis, normalising the data with the initial and final time points set to 0 and 100 (arbitrary units), respectively. To measure signal intensity in the deGradFP experiments, we generated SUM projections and traced a line crossing control and experimental regions (marked by the expression of *btl* > CD4::IFP or *en>*nls::mCherry) within each embryo. We plotted the fluorescence intensity along this line in both channels and calculated the correlation coefficient for the signal from the fluorescent reporter with respect to the signal of the target protein, β-integrin::GFP.

No blinding was performed in any of our experiments. All our experiments represent pooled data from at least three independent crosses. We did not use statistical methods to determine sample size. All data were analysed and plotted using R Studio or Prism. For statistical analyses, the normal distribution of the data was tested in Prism (version 10.5.0).

## Supplementary information

Peer Review File

Movie EV1

Movie EV2

Movie EV3

Movie EV4

Movie EV5

Movie EV6

Movie EV7

Movie EV8

Movie EV9

Source data Fig. 1

Source data Fig. 3

Source data Fig. 4

Source data Fig. 5

Source data Fig. 6

Source data Fig. 7

Figure EV1 Source Data

Figure EV3 Source Data

Figure EV4 Source Data

Figure EV5 Source Data

Expanded View Figures

## Data Availability

The imaging data from this publication have been deposited to BioImage Archive and assigned the identifier S-BIAD2751. The source data of this paper are collected in the following database record: biostudies:S-SCDT-10_1038-S44319-026-00754-z.
