# Supplementary material for: Adhesion to a common ECM mediates interdependence in tissue morphogenesis in Drosophila
Source: EMBO Rep. 2026 Apr 1;27(11):2893–914. doi: 10.1038/s44319-026-00754-z (PMC13260368; doi:10.1038/s44319-026-00754-z)
Supplement: Supplementary file 2 — Movie EV1 [file 44319_2026_754_MOESM2_ESM.zip › Movie EV1/Movie EV1.docx]

**Movie EV1. Time-lapse imaging of tracheal trunk development using MuVi-SPIM.** Maximum intensity projection of an embryo expressing CD4::mIFP under *btl-gal4.*
