# Supplementary material for: Adhesion to a common ECM mediates interdependence in tissue morphogenesis in Drosophila
Source: EMBO Rep. 2026 Apr 1;27(11):2893–914. doi: 10.1038/s44319-026-00754-z (PMC13260368; doi:10.1038/s44319-026-00754-z)
Supplement: Supplementary file 3 — Movie EV2 [file 44319_2026_754_MOESM3_ESM.zip › Movie EV2/Movie EV2.docx]

**Movie EV2. Time-lapse imaging of embryos expressing Talin::YPet and *btl*>CD4::mIFP.** Maximum intensity of embryos with or without dorsal branches. Control embryo (first) and embryo expressing Dad under *btl-gal4* (last). *btl*>CD4::mIFP is shown in red and Talin::YPet in cyan.
