# Supplementary material for: Adhesion to a common ECM mediates interdependence in tissue morphogenesis in Drosophila
Source: EMBO Rep. 2026 Apr 1;27(11):2893–914. doi: 10.1038/s44319-026-00754-z (PMC13260368; doi:10.1038/s44319-026-00754-z)
Supplement: Supplementary file 4 — Movie EV3 [file 44319_2026_754_MOESM4_ESM.zip › Movie EV3/Movie EV3.docx]

**Movie EV3. Time-lapse imaging of tracheal development using MuVi-SPIM.** Maximum intensity projection of an embryo expressing CD4::mIFP under *btl-gal4*. Asterisks mark protruding cells of the tracheal dorsal trunk.
