# Supplementary material for: Adhesion to a common ECM mediates interdependence in tissue morphogenesis in Drosophila
Source: EMBO Rep. 2026 Apr 1;27(11):2893–914. doi: 10.1038/s44319-026-00754-z (PMC13260368; doi:10.1038/s44319-026-00754-z)
Supplement: Supplementary file 5 — Movie EV4 [file 44319_2026_754_MOESM5_ESM.zip › Movie EV4/Movie EV4.docx]

**Movie EV4. Time-lapse imaging of embryos expressing *sqh*-Gap43::mCherry and *btl*>CD4::mIFP.** The red dot indicates the protrusions that tracheal cells form towards the epidermis. *btl*>CD4::mIFP is shown in red and *sqh-*Gap43::mCherry in cyan.
