# Supplementary material for: Adhesion to a common ECM mediates interdependence in tissue morphogenesis in Drosophila
Source: EMBO Rep. 2026 Apr 1;27(11):2893–914. doi: 10.1038/s44319-026-00754-z (PMC13260368; doi:10.1038/s44319-026-00754-z)
Supplement: Supplementary file 6 — Movie EV5 [file 44319_2026_754_MOESM6_ESM.zip › Movie EV5/Movie EV5.docx]

**Movie EV5. Time-lapse imaging of embryos expressing *btl*>Utr::GFP and *btl*>CD4::mIFP.** High temporal-resolution imaging of protruding cells visualised using *btl*>Utr::GFP (actin, cyan) and *btl*>CD4::mIFP (membrane, red).
