# Supplementary material for: Adhesion to a common ECM mediates interdependence in tissue morphogenesis in Drosophila
Source: EMBO Rep. 2026 Apr 1;27(11):2893–914. doi: 10.1038/s44319-026-00754-z (PMC13260368; doi:10.1038/s44319-026-00754-z)
Supplement: Supplementary file 7 — Movie EV6 [file 44319_2026_754_MOESM7_ESM.zip › Movie EV6/Movie EV6.docx]

**Movie EV6. Time-lapse imaging of embryos expressing btl>CD4::mIFP, Dad.** Maximum intensity projection of embryos expressing Dad under *btl-gal4.*
