# Supplementary material for: Adhesion to a common ECM mediates interdependence in tissue morphogenesis in Drosophila
Source: EMBO Rep. 2026 Apr 1;27(11):2893–914. doi: 10.1038/s44319-026-00754-z (PMC13260368; doi:10.1038/s44319-026-00754-z)
Supplement: Supplementary file 8 — Movie EV7 [file 44319_2026_754_MOESM8_ESM.zip › Movie EV7/Movie EV7.docx]

**Movie EV7. Laser microdissection experiments.** Maximum intensity projection of embryos expressing Utr::GFP under *btl-gal4.* Laser microdissection was performed in protruding cells and tip cells, respectively.
