# Supplementary material for: Adhesion to a common ECM mediates interdependence in tissue morphogenesis in Drosophila
Source: EMBO Rep. 2026 Apr 1;27(11):2893–914. doi: 10.1038/s44319-026-00754-z (PMC13260368; doi:10.1038/s44319-026-00754-z)
Supplement: Supplementary file 9 — Movie EV8 [file 44319_2026_754_MOESM9_ESM.zip › Movie EV8/Movie EV8.docx]

**Movie EV8. Time-lapse imaging of embryos expressing β-Integrin::GFP and *btl*>CD4::mIFP.** Maximum intensity projection of a control embryo (left) and an embryo expressing deGradFP under *btl>gal4* (right). *btl*>CD4::mIFP is shown in red and β-Integrin::GFP in cyan**.**
