# Supplementary material for: Adhesion to a common ECM mediates interdependence in tissue morphogenesis in Drosophila
Source: EMBO Rep. 2026 Apr 1;27(11):2893–914. doi: 10.1038/s44319-026-00754-z (PMC13260368; doi:10.1038/s44319-026-00754-z)
Supplement: Supplementary file 10 — Movie EV9 [file 44319_2026_754_MOESM10_ESM.zip › Movie EV9/Movie EV9.docx]

**Movie EV9. Time-lapse imaging of embryos expressing DE-Cadherin::GFP and *btl*>CD4::mIFP.** Maximum intensity projection of a control embryo (left) and a *kay^1^* mutant embryo. *btl*>CD4::mIFP is shown in red and DE-Cadherin::GFP in cyan.
