# Supplementary material for: Adhesion to a common ECM mediates interdependence in tissue morphogenesis in Drosophila
Source: EMBO Rep. 2026 Apr 1;27(11):2893–914. doi: 10.1038/s44319-026-00754-z (PMC13260368; doi:10.1038/s44319-026-00754-z)
Supplement: Supplementary file 21 — Expanded View Figures [file 44319_2026_754_MOESM21_ESM.pdf]

Expanded View Figures

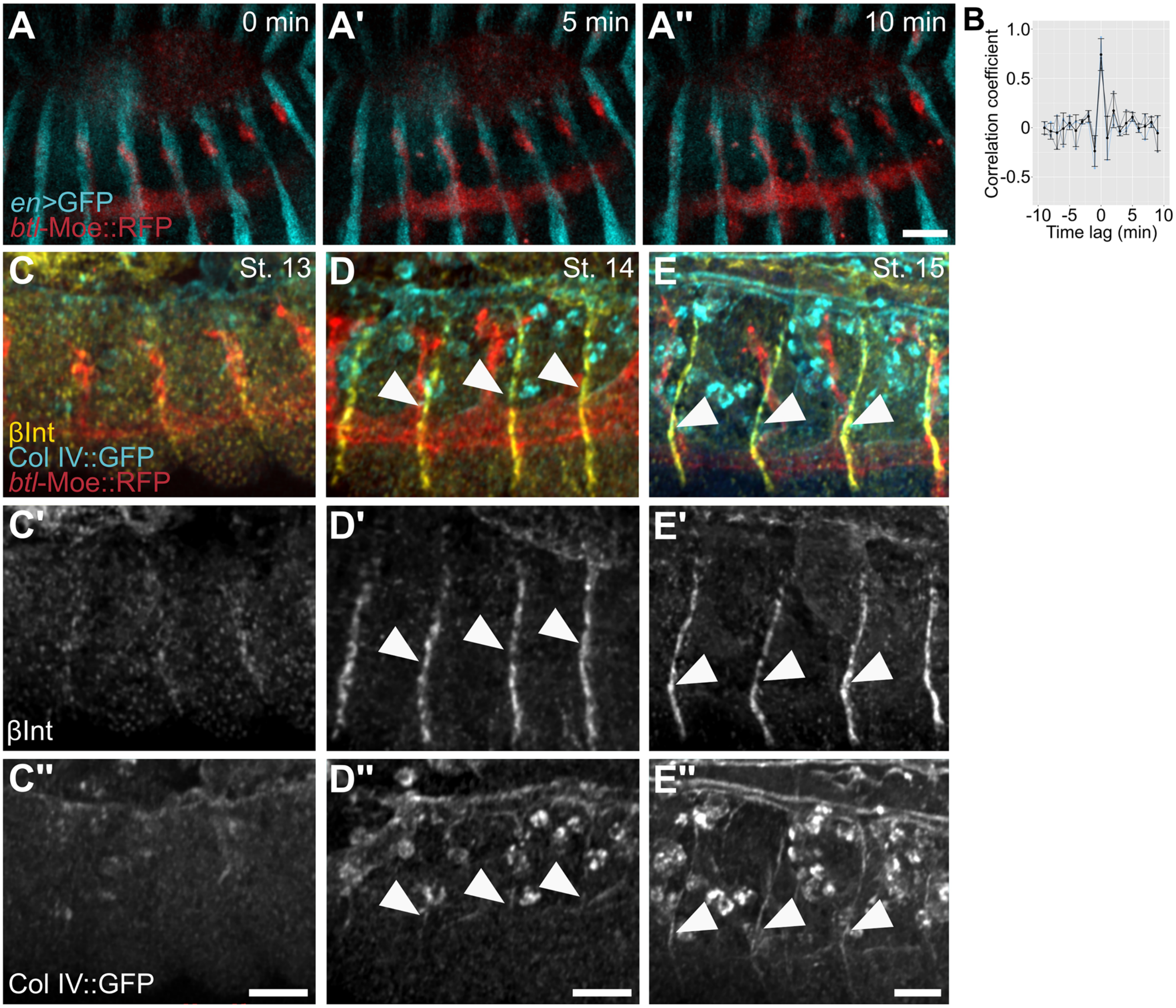

Figure EV1. Analysis of epidermis-trachea coordination and distribution of Collagen IV::GFP and β-Integrin.

(**A–A″**) Cartographical maximum intensity projection of confocal stacks showing stripes of epidermis using *en* > GFP (cyan) and the tracheal system using *btl*-Moe::RFP (red). (**B**) Cross-correlation analysis of *en* > GFP and *btl*-Moe::RFP signal displacement, $n = 3$ embryos. (**C–E**) Distribution of Collagen IV:: GFP (cyan) and β-Integrin (yellow) in stages 13–15 in relation to the tracheal system labelled with *btl*-MoeRFP (red). (**C–C″**) Stage 13; (**D–D″**) Stage 14; (**E–E″**) Stage 15. Arrowheads point to muscle attachment sites at their intersection with tracheal trunks. Data are plotted as mean ± SD. (**A**) Scale bar: 25 µm. (**C–E**) Scale bars: 20 µm. Source data are available online for this figure

© The Author(s)

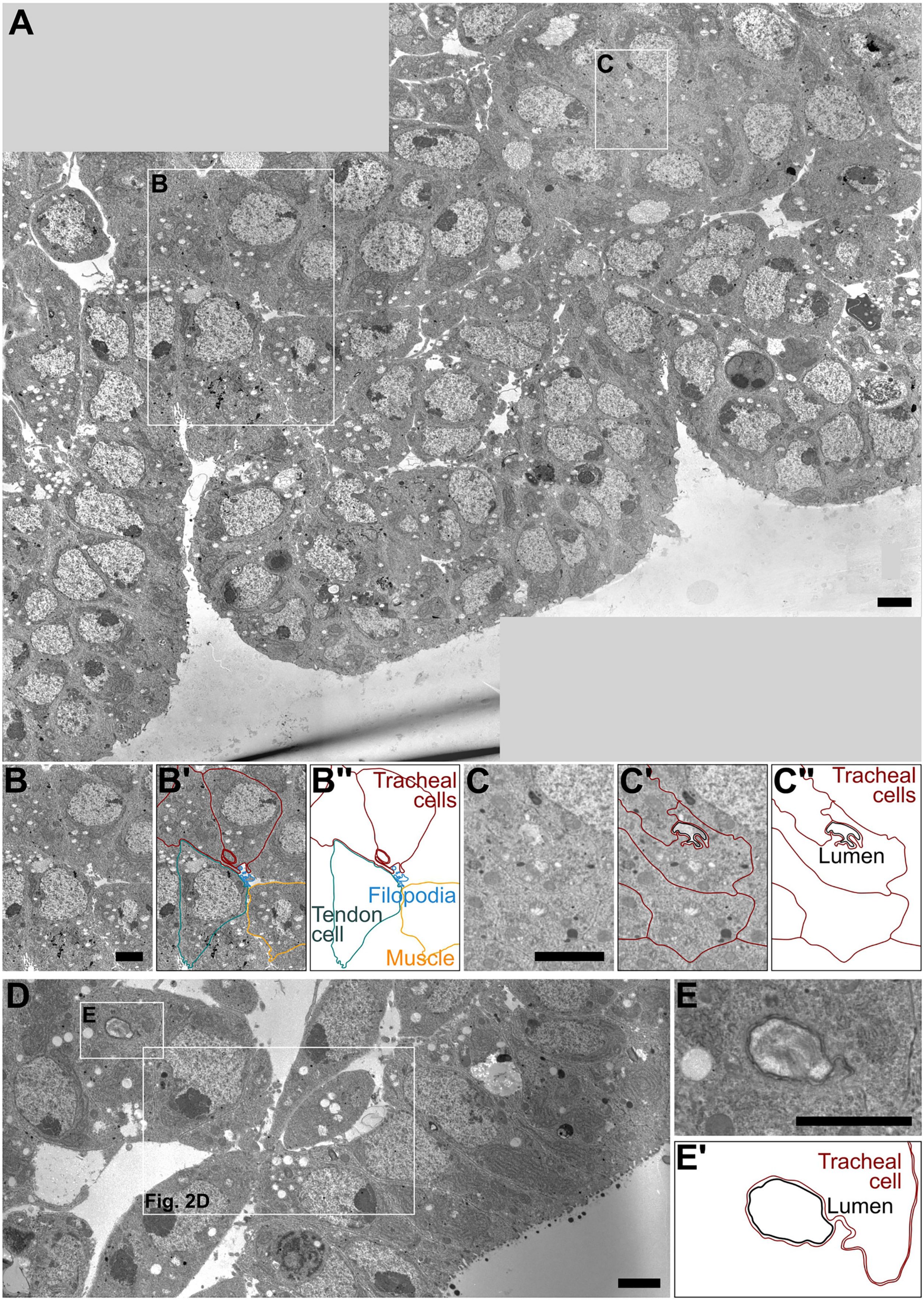
A
B
C
B
B'
B''
Tracheal cells
Filopodia
Tendon cell
Muscle
C
C'
C''
Tracheal cells
Lumen
D
E
Fig. 2D
E
E'
Tracheal cell
Lumen

Figure EV2. Contact points between the epidermis and the tracheal trunks (other examples).

(**A**) Electron micrograph of a longitudinal section of the dorsal trunk, stage 14 embryo. The regions indicated by the white squares are magnified in (B and C). (**B**) Magnification of a contact point between tracheal trunks and a tendon cell. (**B′**, **B″**) Manual tracings of the elements present in (**B**). (**C**) Magnification of a region of the tracheal trunk where the lumen is visible. (**C′**, **C″**) Manual tracings of the elements present in (**C**). (**D**) Overview image of the micrograph magnified in Fig. 2D. (**E**) Magnified view of the tracheal lumen illustrated in (**D**). (**E′**) Manual tracings of the elements present in (**E**). Scale bars: 2 µm.

© The Author(s)

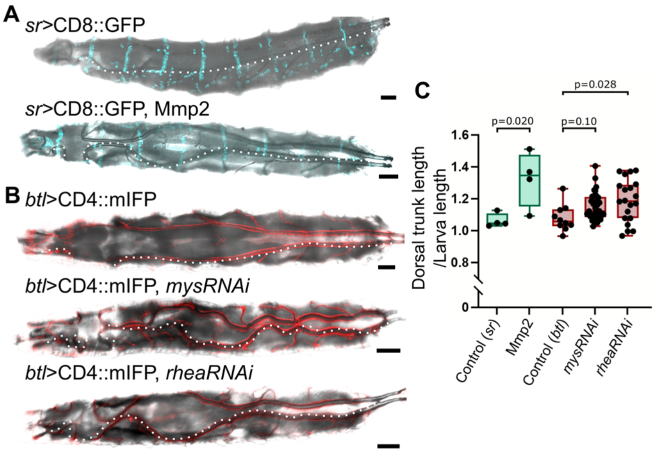

Figure EV3. Role of adhesion complexes and ECM in larval dorsal trunk morphology.

(**A–B**) Overlay of maximum-intensity projections of fluorescent reporters and minimum-intensity projections of reflected light images of heat-fixed third instar larvae. (**A**) Larvae expressing GFP under *sr-gal4*. Top panel, control; bottom panel, expression of Mmp2. (**B**) Larvae expressing CD4::mIFP under *btl-gal4*. Top panel, control; middle panel, *mys* (which codes for β-integrin) RNAi; bottom panel, *rhea* (which codes for Talin) RNAi. (**C**) Normalised dorsal trunk (DT) length. Box plot represents median, IQR and min and max values. Control (*sr*), $n = 4$ larvae; Mmp2, $n = 4$ larvae; Control (*btl*), $n = 10$ larvae; *mysRNAi*, $n = 33$ larvae; *rheaRNAi*, $n = 20$ larvae. Significance was determined using ANOVA and the Kruskal–Wallis test for multiple comparisons. Scale bars: 200 µm. Source data are available online for this figure

 © The Author(s)

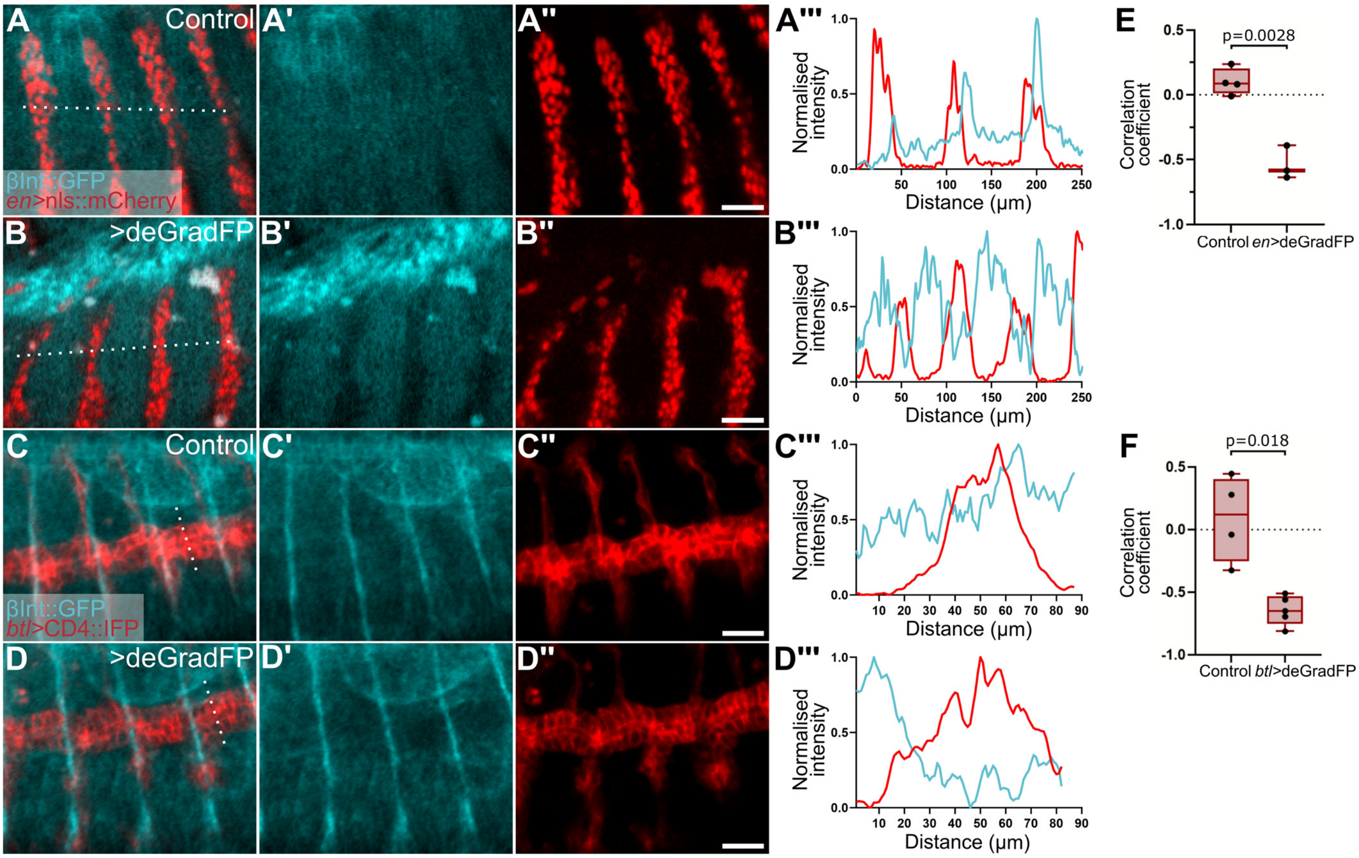

Figure EV4. Effect of deGradFP expression on β-Integrin::GFP fluorescence intensity.

(**A–D**) Maximum intensity projections of embryos expressing β-Integrin::GFP (cyan). (**A–B**) Embryos additionally expressing nls::mCherry (red) under *en-gal4*. (**C–D**) Maximum intensity projections of embryos expressing CD4::mIFP under *btl-gal4* (red) and β-Integrin::GFP (cyan). (**A**, **C**) Controls; (**B**, **D**) deGradFP expression. (**A‴–D‴**) Fluorescence intensity profiles across the dotted white lines drawn in (**A–D**). (**E**, **F**) Correlation analyses of fluorescence intensity profiles comparing signals from *en*>deGradFP vs controls (**E**; Control, $n = 4$; *en*>deGradFP, $n = 3$) and *btl*>deGradFP vs controls (**F**; Control, $n = 4$; *btl*>deGradFP, $n = 5$). Box plots represent median, IQR and min and max values. Significance was determined using *t* tests. Scale bars: 20 µm. Source data are available online for this figure

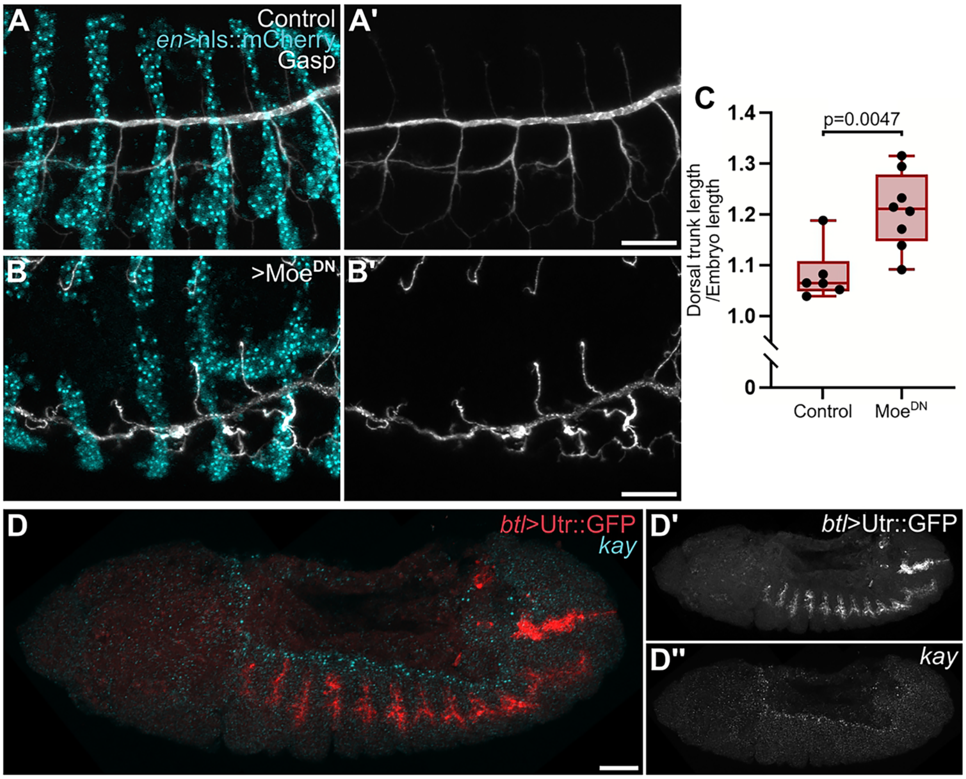

Figure EV5. Role of the dorsal closure progression in tracheal trunk morphology.

(**A–B**) Maximum intensity projections of embryos expressing nls::mCherry under *en-gal4* and immunostained for Gasp. (**A**) Control (**B**) Embryo with additional expression of Moe^{DN}. (**C**) Quantification of normalised dorsal trunk length. Control, $n = 6$ embryos; Moe^{DN}, $n = 8$ embryos). Significance was assessed using *t* test. Box plot represents mean, IQR and min and max value. (**D–D″**) HCR-FISH against *kay* (cyan) in embryos with expression of Utr::GFP under *btl-gal4* (red). Scale bars: 30 µm. Source data are available online for this figure

 © The Author(s)
