# Supplementary material for: Adhesion to a common ECM mediates interdependence in tissue morphogenesis in Drosophila
Source: EMBO Rep. 2026 Apr 1;27(11):2893–914. doi: 10.1038/s44319-026-00754-z (PMC13260368; doi:10.1038/s44319-026-00754-z)
Supplement: Supplementary file 1 — Peer Review File [file 44319_2026_754_MOESM1_ESM.pdf]

EMBO reports

Adhesion to a common ECM mediates interdependence in tissue morphogenesis in Drosophila

Luis Sánchez-Cisneros, Mariana Barrera-Velázquez, Dimitri Kromm, Philippe Bun, Horacio Merchant-Larios, and Luis Daniel Rios-Barrera

Corresponding author(s): Luis Daniel Rios-Barrera (daniel.rios@iibiomedicas.unam.mx)

Review Timeline:

Transfer Date:	8th Jul 25
Editorial Decision:	22nd Jul 25
Revision Received:	10th Dec 25
Editorial Decision:	5th Feb 26
Revision Received:	25th Feb 26
Accepted:	12th Mar 26

Editor: Achim Breiling

Transaction Report: This manuscript was transferred to EMBO reports following peer review at Review Commons.

(Note: With the exception of the correction of typographical or spelling errors that could be a source of ambiguity, letters and reports are not edited. Depending on transfer agreements, referee reports obtained elsewhere may or may not be included in this compilation. Referee reports are anonymous unless the Referee chooses to sign their reports.)

Revision Plan

Manuscript number: #RC-2025-02961
Corresponding author(s): Luis Daniel Ríos-Barrera

[The "revision plan" should delineate the revisions that authors intend to carry out in response to the points raised by the referees. It also provides the authors with the opportunity to explain their view of the paper and of the referee reports.

The document is important for the editors of affiliate journals when they make a first decision on the transferred manuscript. It will also be useful to readers of the reprint and help them to obtain a balanced view of the paper.

1. General Statements [optional]

We would like to thank the reviewers for their comments, we see great value in the suggestions they made to strengthen our work. We are glad to see that they are in general positive about the manuscript. In the following, we include a point-by-point response to their comments, which are in general consistent with each other.

2. Description of the planned revisions

Insert here a point-by-point reply that explains what revisions, additional experimentations and analyses are planned to address the points raised by the referees.

Reviewer #1 (Evidence, reproducibility and clarity (Required)):

In this manuscript, Sanchez-Cisneros and colleagues, examine how tracheal cell adhesion to the ECM underneath the epidermis helps shape the tracheal system. They show that if cell-ECM adhesion is perturbed the development of the tracheal system and the epidermis is disrupted. They also detect protrusions extending from the dorsal trunk cells towards the ECM. The work is novel, the figures are clear, and the questions are well addressed. However, I find that some of the claims are not completely supported by the data presented. I have some suggestions that will, I believe, clarify certain points.

Major comments

At the beginning of the results section as in the introduction the authors claim that "It is generally assumed that trunk displacement occurs due to tip cells pulling on the trunks so that they follow their path dorsally." This sentence is not referenced, and I do not know where it has been shown or proposed to be like this. In addition, the comparison with the ventral branches is also not referenced and the movie does not really show this. Forces generated by tracheal branch migration have been shown to drive intercalation (Caussinus E, Colombelli J, Affolter M. Tip-cell migration controls stalk-cell intercalation during Drosophila tracheal tube elongation. Curr Biol. 2008;18(22):1727-1734. doi:10.1016/j.cub.2008.10.062), but not dorsal trunk (DT) displacement.

We agree that dorsal trunk displacement has not been discussed in previous works, just the fact that tip-cell migration influences stalk cell intercalation. We will rephrase this sentence, stating that dorsal trunk displacement has not been studied.

However, to rule out the possibility that DT displacement and the phenotype observed in XXX is due to dorsal branch pulling forces, the authors should analyze what happens in the absence of dorsal branches (in condition of Dpp signalling inhibition as in punt mutants or Dad overexpression conditions).

This is a great idea, and we thank the reviewer for suggesting this. We tried to achieve a similar goal by expressing a Dominant Negative FGFR (Breathless-DN) in the tracheal system, since its expression under *btl-gal4* affects tip cell migration. However, the phenotype arises too late to have an effect in dorsal branch migration during the stages we were interested in analyzing. The alternative proposed by the reviewer should be more efficient, as blocking Dpp signalling prevents the formation of dorsal branches completely. We have just received flies carrying the UAS-Dad construct. We will express Dad under *btl-gal4* and see how this affects dorsal trunk displacement.

I am concerned about the TEM observations. The authors claim they can identify tracheal cells by their lumen (Fig. 2 C'). However, at stage 15, the tracheal lumen should be clearly identifiable, and the interluminal DT space should be wider relative to the size of the cells. In this case, there is nothing telling us that we are not looking at a dorsal branch or lateral trunk cell. Furthermore, at embryonic stage 15, the tracheal lumen is filled with a chitin filament, which is not visible in these micrographs. Also, there is quite a lot of tissue detachment and empty spaces between cells, which might be a sign of problems in sample fixing. Better images and more accurate identification of dorsal trunk cells is necessary to support the claim that "These experiments revealed a novel anatomical contact between the epidermis and tracheal trunks".

The protocol that we use for TEM involves performing 1-μm sections that allow us to stage embryos and to identify the anatomical regions using light microscopy and then switch to ultra-thin sections for electron microscopy once we have found the right position within the sample. This approach also allows us to determine the integrity of the sample. We attach here a micrograph of the last section we analyzed before we decided to do the EM analysis. The asterisk (*) points to a region where the multicellular lumen of the trunk is visible. Due to its proximity to the posterior spiracles, we are confident this is the dorsal trunk and not the lateral trunk. We realize now, after comparing this image with an atlas of development (Campos-Ortega and Hartenstein, 2013), that the stage we chose to illustrate the interaction is a stage 14 embryo instead of the stage 15 we indicated in the manuscript. We will change the stage but given that dorsal closure has already started by stage 14, this does not affect our analysis. Still, we apologize for the mis-staging of the embryo.

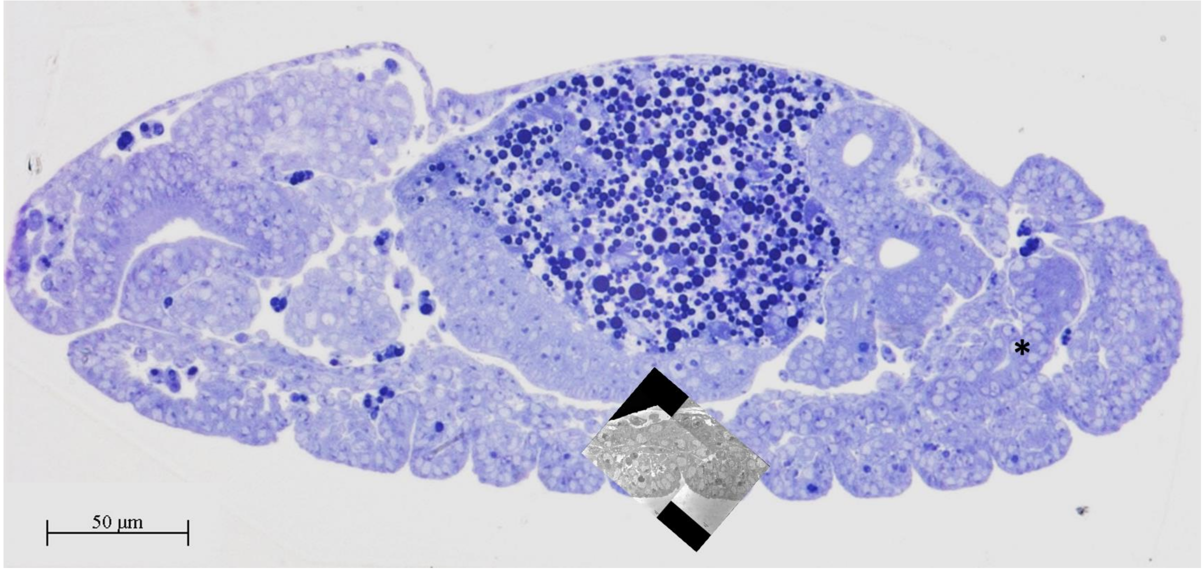

In the light-microscopy image, we have overlaid the EM section to the corresponding region of interest. We agree that the lumen should be thicker compared to the length of the cells, if the section would be cutting the trunk through its largest

diameter. However, the protrusions we see do not emerge from the middle part of the trunk where the lumen is found but are seen towards the dorsal side of the trunk, where the lumen will no longer be visible in a longitudinal section as the ones we present. In the embryo shown in Figure 2A-C, our interpretation is that the section was done through a very shallow section of the lumen (represented below). We interpret this from the fact that we see abundant electron-dense areas which we think are adherens junctions from multiple cells. These junctions are visible in Figure 2C but are currently not labelled. We will add arrows to increase their visibility:

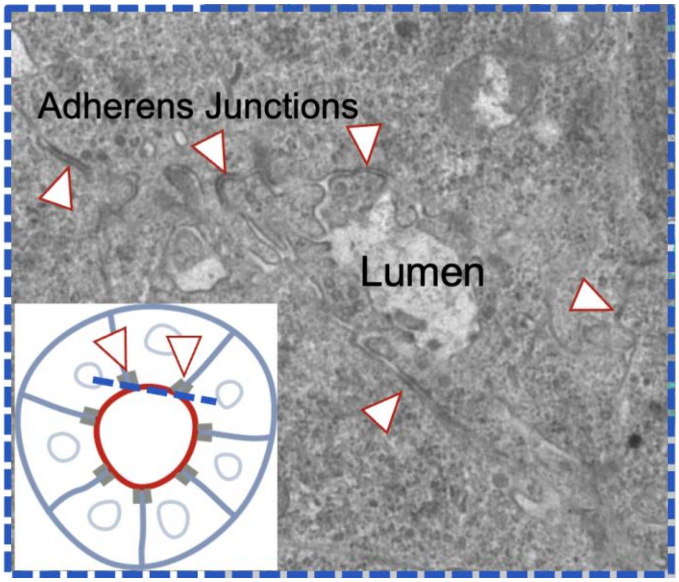

Given that protruding cells lie at the base of dorsal branches, it would be expected that in some sections we would find the protrusions close to the dorsal branches. This is in fact what we show in the micrograph shown in Figure 2D, with a lower magnification overview image shown in Figure S2D. In this case, we see a cell in close proximity to the tendon cells on one side (Figure 2D), which is connected to a dorsal branch on the opposite side (shown in Figure S2D). This dorsal branch is clearly autocellular and chitin deposition is visible as expected for the developmental stage. Again, in Figure S2E we see an electron-dense patch near the lumen that corresponds to the adherens junctions that seal the lumen. We see that all this needs to be better explained in the manuscript, so we will elaborate on the descriptions, and incorporate the light microscopy micrograph to the supplemental figures. This should also aid with the anatomical descriptions requested by Reviewer #3. Nevertheless, we think these observations confirm that what we are describing are the contact points between the dorsal trunk and tendon cells.

Timelapse imaging of the protrusions in DT cells is done with frames every 4 minutes (Video S3). This is not enough to properly show cellular protrusions and the images do not really show interaction with the epidermis. Video S4 has a better time resolution but it is very short and only shows the cut moment. Video S4, shows the cut, but the reported (and quantified recoil) is not clear. Nevertheless, the results are noteworthy and should be further analysed.

We will acquire high temporal resolution time-lapse images using E-Cadherin::GFP and *btl-gal4*, *UAS*-PH::mCherry to show the behaviour of the protrusions on a short time scale.

Provided these embryos survive, would it be possible to check if embryos after laser cutting will develop wavy DTs?

We think it would be interesting to carry out this experiment, but the laser cut experiments were done under a collaborative visit and we would not be able to repeat it in a short-term period.

What happens to the larvae under the genetic conditions presented in Fig.S3? Do they reach pupal stages? Do these animals reach adult stages?

We have seen escapers out of these crosses, but we have not quantified the lethality of the experiment. We will analyse this and include it in the manuscript.

The kayak phenotypes are very interesting and perhaps the authors could explore them more. As in inhibition of adhesion to the ECM, kay mutants display wavy dorsal trunks. Do they have defective adhesion? Fos being a transcription factor, this is a possibility. The authors should at least discuss the kay phenotypes more extensively and present a suitable hypothesis for the phenotype.

We agree that the *kayak* experiments might bring more consequences than just preventing dorsal closure. We will complement this approach by blocking dorsal closure by other independent means. We will use *pannier-gal4* (a lateral epidermis driver), *engrailed-gal4* (a driver for epidermal posterior compartment), and *332-gal4* (an amnioserosa driver) to express dominant-negative Moesin. In our experience, this also delays dorsal closure and it should result in a similar tracheal phenotype as the one we see in *kayak* embryos.

Minor comments

Page 2 Line 9/10 The sentence "tracheal tubes branch and migrate over neighbouring tissues of different biochemical and mechanical properties to ventilate them." should be rewritten. Tracheal cells do not migrate over other tissues to ventilate them.

We meant to say that tracheal cells migrate over other tissues at the same time as they branch and interconnect to allow gas exchange in their surroundings after tracheal morphogenesis is completed. Ventilation is used here as a synonym for gas exchange or breathing. We will rephrase this if the reviewer considers it confusing.

Page 2 Line 24/25 The sentence "It has been generally assumed that trunks reach the dorsal side of the embryo because of the pulling forces of dorsal branch migration." needs to be backed up by a reference.

As explained above, we will rephrase this sentence.

Page 7 Line 32/23 In this sentence, the references are not related to dorsal closure "Similarly, the signals that regulate epidermal dorsal closure do not participate in tracheal development, or vice versa (Letizia et al., 2023; Reichman-Fried et al., 1994)."

Our goal in this sentence was to explain that while JNK is required for proper epidermal dorsal closure, loss of JNK signaling in the trachea does not affect tracheal development (as shown by Letizia et al., 2023). At the same time, Reichman-Fried et al., 1994 described the phenotypes of loss of *breathless (btl)*. We will remove this last reference as the work does not study the epidermis. We will rephrase the sentence as: "Similarly, the signals that regulate epidermal dorsal closure do not participate in tracheal development; namely, JNK signaling (Letizia et al., 2023)."

Page 12 Line 1 "Muscles attach to epidermal tendon cells through a dense meshwork of ECM" this sentence must be referenced.

We will add the corresponding references for this statement: (Fogerty et al., 1994; Prokop et al., 1998; Urbano et al., 2009). We will change "dense" for "specialized".

Fig. S1- Single channel images (A'-C' and A'-C') should be presented in grayscale.

Fig. S4- Single channel images (A'-D' and A'-D') should be presented in grayscale.

We will add the grayscale, single-channel images for these figures.

Reviewer #1 (Significance (Required)):

The findings shown in this manuscript shed light on the interactions and cooperation between two organs, the tracheal system and the epidermis. These interactions are mediated by cell-ECM contacts which are important for the correct morphogenesis of both systems. The strengths of the work lie on its novelty and live analysis of these interactions. However, its weaknesses are related to some claims not completely backed by the data, some technical issues regarding imaging and some over-interpreted conclusions.

This basic research work will be of interest to a broad cell and developmental biology community as they provide a functional advance on the importance of cell-ECM interactions for the morphogenesis of a tubular organ. It is of specific interest to the specialized field of tubulogenesis and tracheal morphogenesis.

Reviewer #2 (Evidence, reproducibility and clarity (Required)):

Summary: In this paper, the authors explore the relationships between two Drosophila tissues - the epidermis and tracheal dorsal trunk (DT) - that get dorsally displaced during mid-late embryogenesis. The show a nice temporal correlation between the movements of the epithelia during dorsal closure and DT displacement. They also show a correlation between the movement of an endogenously tagged version of collagen and the DT, suggesting that the ECM may contribute to this coordinated movement. Through high magnification TEM, they show that tracheal cells make direct contact with the subset of epithelial cells, known as tendon cells, that also serve as muscle attachment sites. In between these contact sites, tracheae are separated from the epithelia by the muscles. Furthermore, the TEMs and confocal imaging of tracheal cells expressing a membrane marker at these contact sites show that the tracheal cells are extending filopodia toward the tendon cells. The authors then explore how a variety of perturbations to the ECM produced by the tendon and DT cells affect DT and epithelial movement. They find that expressing membrane-associated matrix metalloproteases (MMP1 or MMP2) in tendon cells as well as perturbations in integrin or integrin signaling components leads to delays in dorsal displacement as well as defective lengthening of the tracheal DT tubes. They find that defects in the association between the tracheal and epidermal ECM attachments affect dorsal displacement of the epidermis, disrupting dorsal closure.

Major comments: I like the goals of this paper testing the idea that the ECM plays important roles in the coordination of tissue placement, and I think they have good evidence of that from this study. However, I disagree with the conclusions of the authors that disrupting contact between DT and the tendon cells has no effect on DT dorsal displacement. DT tracheal positioning is clearly delayed; the fact that it takes a lot longer indicates that the ECM does affect the process. It's just that there are likely backup systems in place - clearly not as good since the tracheal tubes end up being the wrong length.

We agree with this view; in our deGradFP experiments we see a delayed DT displacement. We focused our analyses on the coordination with epidermal remodelling, which remained unaltered, but we in fact see a delayed progression in dorsal displacement of both tissues (Figure 5I-J). We will emphasize this in the corresponding section of the Results.

It also seems important that the parts of the DT where the dorsal branches (DB) emanate are moving dorsally ahead of the intervening portions of the trachea. This suggests to me that the DB normally does contribute to DT dorsal displacement and that this activity may be what helps the DT eventually get into its final position. The authors should test whether the portions of the DT that contact the DB are under tension. If the DB migration is providing some dorsal pulling force on the DT, this may also contribute to the observed increases in DT length observed with the perturbations of the ECM between the tendon cells and the trachea - if tube lengthening is a consequence of the pulling forces that would be created by parts of the trachea

moving dorsally ahead of the other parts. Here again, it would be good to test if the DT itself is under additional tension when the ECM is disrupted.

We thank the reviewer for the suggested experiments. We agree with the fact that the dorsal branches should pull on the dorsal trunk and that this interaction should generate tension. Unfortunately, we are unable to test this with the experiments proposed by the reviewer, but we propose an alternative strategy to overcome this. We understand that the reviewer suggests we do laser cut experiments in dorsal branches to see if there is a recoil in the opposite direction of dorsal branch migration. We carried out our laser cut experiments using a 2-photon laser through a visit to the EMBL imaging facility, using funds from a collaborative grant. Funding a second visit would require us to apply for extra funding, which would delay the preparation of the experiments. We are aware of UV-laser setups within our university, however, UV-laser cuts would also affect the epidermis above the dorsal branches, which we think might contribute to recoil we would expect to see.

Instead of doing laser cuts, we have designed an experiment based on the suggestion of reviewer #1 of blocking Dpp signaling (with UAS-Dad), which would prevent the formation of dorsal branches. We expect that in this experimental setup, the trunk will bend ventrally in response to the pulling forces of the ventral branches. We will also co-express UAS-Dad (to prevent dorsal branch formation) and UAS-Mmp2 (to 'detach' the dorsal trunk from the epidermis), and we would expect to at least partially rescue the wavy trunk phenotype.

Minor comments: The authors need to do a much better job in the intro and in the discussion of citing the work of the people who made many of the original findings that are relevant to this study. Many citations are missing (especially in the introduction) or the authors cite their own review (which most people will not have read) for almost everything (especially in the discussion). This fails to give credit to decades of work by many other groups and makes it necessary for someone who would want to see the original work to first consult the review before they can find the appropriate reference. I know it saves space (and effort) but I think citing the original work is important.

The reviewer is right; we apologize for falling into this practice. We will reference the original works wherever it is needed.

Figure 7 is not a model. It is a cartoon depicting what they see with confocal and TEM images.

We will change the figure; we will include our interpretations of the phenotypes we observed under different experimental manipulations.

Reviewer #2 (Significance (Required)):

Overall, this study is one of the first to focus on how the ECM affects coordination of tissue placement. The coordination of tracheal movement with that of the epidermis is very nicely documented here and the observation that the trachea make direct contact with the tendon cells/muscle attachment sites is quite convincing. It is less clear from the data how exactly the cells of the trachea and the ECM are affected by the different perturbations of the ECM. It seems like this could be better done with immunostaining of ECM proteins (collagen-GFP?), cell type markers, and super resolution confocal imaging with combinations of these markers. What happens right at the contact site between the tendon cell and the trachea with the perturbation? I think that at the level of analysis presented here, this study would be most appropriate for a specialized audience working in the ECM or fly embryo development field.

Reviewer #3 (Evidence, reproducibility and clarity (Required)):

Summary

The manuscript by Sanchez-Cisneros et al provides a detailed description of the cellular interactions between cells of the Drosophila embryonic trachea and nearby tendon and epidermal cells. The researchers use a combination of genetic experiments, light sheet style live imaging and transmission electron microscopy. The live imaging is particularly clear and

detailed, and reveals protruding cells. The results overall suggest that interactions mediated through the ECM contribute to development of trachea and dorsal closure of epidermis. One new aspect is the existence of dorsal trunk filipodia that are under tension and may impact tracheal morphogenesis through required integrin/ECM interactions.

Major comments:
- Are the key conclusions convincing?
Generally, the key conclusions are well supported by the data, and the movies are very impressive. Interactions between the cell types are clearly shown, as is the correlations in their development. However, some of the images are challenging to decipher for a non-expert in Drosophila trachea, especially the EM images, and some of the data is indirect or a bit weak.

We thank the reviewer for their observations. As mentioned above in response to Reviewer #1, we will add an overview image of the embryo we processed for TEM that is presented in Figure 2.

The data related to failure of dorsal closure affecting trachea relies on one homozygous allele of one gene (kayak), and so this is somewhat weak evidence. Even though kay is not detected in trachea, there could be secondary effects of the mutation or another lesion on the mutant chromosome. The segments look a bit uneven in the mutant examples.

The reviewer is right; as we proposed before, we will complement the *kayak* experiments with independent approaches that will delay dorsal closure.

- Should the authors qualify some of their claims as preliminary or speculative, or remove them altogether?
Some of the experiments have low n values, especially in imaging experiments, so these may be more preliminary, but they are in concordance with other data.

The problem we face in our live-imaging experiments is related to the probability of finding the experimental embryos. In most of our experiments we combine double-tissue labelling plus the expression of genetic tools. This generally corresponds to a very small proportion of the progeny. We will aim to have at least 4 embryos per condition.

- Would additional experiments be essential to support the claims of the paper? Request additional experiments only where necessary for the paper as it is, and do not ask authors to open new lines of experimentation.
Higher n-values would substantiate the claims.
To strengthen the argument that dorsal closure affects trachea morphogenesis mechanically, the authors might consider using of a combination of kay mutant alleles or other mutant genes in this pathway to provide stronger evidence. Or they could try a rescue experiment in epidermis and trachea separately for the kay mutants.

We think our experiments delaying dorsal closure using the Gal4/UAS system and a variety of drivers should address the point of the possible indirect effects of *kay* in tracheal development.

- Are the suggested experiments realistic in terms of time and resources? It would help if you could add an estimated cost and time investment for substantial experiments.
Imaging data can take awhile to obtain, but the genetic experiments could be done in a couple of months, and the authors should be able to obtain any needed lines within a few weeks.

The reviewer is correct, we will be able to plan our crosses for the proposed experiments within a couple of months.

- Are the data and the methods presented in such a way that they can be reproduced?
Generally, yes. For the deGrad experiments, it is not clear how the fluorescent intensity was normalized - was this against a reference marker?

Briefly, we used signals from within the embryo as internal controls. In the case of *en-gal4*, we normalized the signal to the sections of the embryo where *en* is not expressed and therefore, beta-integrin levels should not be affected. In the case of *btl-gal4*, we normalized against the signal surrounding the trunks which should also not be affected by the deGradFP system. We will elaborate on these analyses in the methods section.

- Are the experiments adequately replicated and statistical analysis adequate?
There are several experiments with low n values, so this could fall below statistical significance. For example, data shown in Fig 1G: n=3; Fig 4D n=4, n=3; Fig 6J n=4

As mentioned above, we will increase our sample sizes.

Minor comments:
- Specific experimental issues that are easily addressable.
To make the TEM images more easily interpreted, it would be helpful to provide a fluorescent image of all the relevant cell types (especially trachea, epidermis, muscle, and tendon cells, plus segmental boundaries) labelled accordingly, so that reader can correlate them more easily with the TEM images. They might also include a schematic of an embryo to show where the TEM field of view is.

We believe this should be addressed by adding the light microscopy section of the embryo with the TEM image overlaid as illustrated above.

It is hard to be confident that the EM images reflect the cells they claim and that the filopodia are in fact that, at least for people not used to looking at these types of images.

As we explained in the response to Reviewer #1, we will elaborate on the descriptions of our TEM data. We think that adding the reference micrograph will aid with the interpretations of the TEM images.

- Are prior studies referenced appropriately?
yes
- Are the text and figures clear and accurate?
yes

- Do you have suggestions that would help the authors improve the presentation of their data and conclusions?
The writing could be revised to be a bit clearer. Since the results of the experiments do not support the initial hypothesis, I found it a bit confusing as I read along. It may help to introduce an alterative hypothesis earlier to make the paper more logical and easy to follow.
To be more specific, On page 3, the authors say they "show that dorsal trunk displacement is mechanically coupled to the remodelling of the epidermis" and also in the results comment that "With two opposing forces pulling the trunks other factors likely participate in their dorsal displacement, but so far these have remained unstudied." But that doesn't end up being what they find. The results from figure 5 and related interpretation on page 17 says "cell-ECM interactions are important for proper trunk morphology, but not for its displacement." So this was confusing to read and I would encourage the authors to frame the issues a bit differently in terms of tube morphogenesis.

We see how this might be confusing. We will rewrite the introduction so that the work is easier to follow. To achieve this, we will state from the beginning the mechanisms we anticipate that regulate trunk displacement: 1) adhesion to the epidermis, 2) pulling forces from the dorsal branches and 3) a combination of both.

Some minor presentation issues:
What orientation is the cross-sectional view in figure 1C and movie 1?

We will add a dotted box that indicates the region that we turned 90° to show the cross-section.

On page 12, the authors say the "Electron micrographs also suggested high filopodial activity" but activity suggests dynamics that are not clear from EM. This could be re-phrased.

As the reviewer indicates, we cannot conclude dynamics from a static image. We will replace "suggested high filopodial activity" with "revealed filopodial abundance".
Reviewer #3 (Significance (Required)):

- Describe the nature and significance of the advance (e.g. conceptual, technical, clinical) for the field.
The results of the paper are significant in that they characterize a mechanical interaction between two tissue types in development, which are linked by the extracellular matrix that sits between them.
It is not clear to me that this describes a "novel mechanism for tissue coordination" as stated in the abstract, but it does characterize this type of interaction in a detailed cellular way.

- Place the work in the context of the existing literature (provide references, where appropriate).
For specialists, the work identifies a novel protruding cell type in the fly embryonic trachea, and provides beautiful and detailed imaging data on tracheal development. The "wavy" trachea phenotype is also uncommon and very interesting, so this result could be linked to the few papers that also describe this phenotype and be built up.

- State what audience might be interested in and influenced by the reported findings.
As it stands, this is most interesting for a specialized audience because it requires some understanding of the development of this system in particular. As it characterizes this to a new level of detail, it could be influential to those in the field. Some addition clarification of the results and re-framing could make the manuscript more clear and interesting for non-specialists.

- Define your field of expertise with a few keywords to help the authors contextualize your point of view. Indicate if there are any parts of the paper that you do not have sufficient expertise to evaluate.
I work with Drosophila and have studied embryonic and adult cell types, although not trachea specifically. I am familiar with all the genetic techniques and imaging techniques used here.

3. Description of the revisions that have already been incorporated in the transferred manuscript

Please insert a point-by-point reply describing the revisions that were <u>already carried out and included</u> in the transferred manuscript. If no revisions have been carried out yet, please leave this section empty.

4. Description of analyses that authors prefer not to carry out

Please include a point-by-point response explaining why some of the requested data or additional analyses <u>might not be necessary or cannot be provided within the scope of a revision</u>. This can be due to time or resource limitations or in case of disagreement about the necessity of such additional data given the scope of the study. Please leave empty if not applicable.

We will incorporate most of the suggestions of the reviewers, which we find very constructive for our manuscript. The two exceptions were already mentioned above but are summarized below:

- As mentioned above, we will not be able to carry out additional laser cut experiments, but with the genetic experiments we intend to do, we think we can partly address the same issues that the laser cut experiments would answer.
- Regarding electron microscopy, we think that incorporating the wide-field images and our detailed descriptions of the data will improve the clarity of our interpretations.

Dear Dr. Rios-Barrera,

Thank you for transferring your manuscript to from Review Commons to EMBO reports. I now went through the manuscript, the referee reports from Review Commons (attached again below) and your revision plan.

Judging from your revision plan, it seems that you will be able to adequately address the concerns of the referees during a major revision. I thus invite you to revise your manuscript accordingly with the understanding that all referee concerns must be addressed in the revised manuscript and/or in a final detailed point-by-point response, as indicated in your revision plan.

Revised manuscripts should be submitted within three months of a request for revision. Please contact me to discuss the revision (also by video chat) if you have further questions or comments regarding the revision, or should you need additional time.

Acceptance of your manuscript will depend on a positive outcome of another round of review at EMBO reports, using the same referees.

When submitting your revised manuscript, please also carefully review the instructions that follow below.

PLEASE NOTE THAT upon resubmission revised manuscripts are subjected to an initial quality control prior to exposition to re-review. Upon failure in the initial quality control, the manuscripts are sent back to the authors, which may lead to delays. Frequent reasons for such a failure are the lack of the data availability section (please see below) and the presence of statistics based on n=2 (the authors are then asked to present scatter plots or provide more data points).

When submitting your revised manuscript, we will require:

1) a .docx formatted version of the final manuscript text (including legends for main figures, EV figures and tables), but without the figures included. Please make sure that changes are highlighted to be clearly visible. Figure legends should be compiled at the end of the manuscript text.

2) individual production quality figure files as .eps, .tif, .jpg (one file per figure), of main figures and EV figures. Please upload these as separate, individual files upon re-submission. Please make sure that all figure panels are called out separately and sequentially in the manuscript text

The Expanded View format, which will be displayed in the main HTML of the paper in a collapsible format, has replaced the Supplementary information. You can submit up to 5 images as Expanded View. Please follow the nomenclature Figure EV1, Figure EV2 etc. The figure legend for these should be included in the main manuscript document file in a section called Expanded View Figure Legends after the main Figure Legends section. Additional Supplementary material should be supplied as a single pdf file labeled Appendix. The Appendix should have page numbers and needs to include a table of content on the first page (with page numbers) and legends for all content. Please follow the nomenclature Appendix Figure Sx, Appendix Table Sx etc. throughout the text, and also label the figures and tables according to this nomenclature.

For more details please refer to our guide to authors:
http://www.embopress.org/page/journal/14693178/authorguide#manuscriptpreparation

See also our guide for figure preparation:
http://wol-prod-cdn.literatumonline.com/pb-assets/embo-site/EMBOPress_Figure_Guidelines_061115-1561436025777.pdf

Moreover, please consult our guidelines for figure legend preparation:
https://www.embopress.org/page/journal/14693178/authorguide#figureformat

3) a .docx formatted letter INCLUDING the reviewers' reports and your detailed point-by-point responses to their comments. As part of the EMBO Press transparent editorial process, the point-by-point response is part of the Review Process File (RPF), which will be published alongside your paper.

4) a complete author checklist, which you can download from our author guidelines (https://www.embopress.org/page/journal/14693178/authorguide). Please insert page numbers in the checklist to indicate where the requested information can be found in the manuscript. The completed author checklist will also be part of the RPF.

Please also follow our guidelines for the use of living organisms, and the respective reporting guidelines:
http://www.embopress.org/page/journal/14693178/authorguide#livingorganisms

5) that primary datasets produced in this study (e.g. RNA-seq, ChIP-seq and array data) are deposited in an appropriate public

database. This is now mandatory (like the COI statement). If no primary datasets have been deposited in any database, please state this in this section (e.g. 'No primary datasets have been generated and deposited').

See also: http://embor.embopress.org/authorguide#datadeposition

Please remember to provide a reviewer password if the datasets are not yet public.

The accession numbers and database should be listed in a formal "Data Availability " section (placed after Materials & Methods) that follows the model below. Please note that the Data Availability Section is restricted to new primary data that are part of this study.

Data availability

The datasets produced in this study are available in the following databases:

- RNA-Seq data: Gene Expression Omnibus GSE46843 (https://www.ncbi.nlm.nih.gov/geo/query/acc.cgi?acc=GSE46843)
- [data type]: [name of the resource] [accession number/identifier/doi] ([URL or identifiers.org/DATABASE:ACCESSION])

*** Note - All links should resolve to a page where the data can be accessed. ***

Moreover, I have these editorial requests:

6) We now request the publication of original source data with the aim of making primary data more accessible and transparent to the reader. You will receive a separate email with instructions for providing source data with your revised manuscript, including information how to upload and organize the files.

7) Our journal encourages inclusion of *data citations in the reference list* to directly cite datasets that were re-used and obtained from public databases. Data citations in the article text are distinct from normal bibliographical citations and should directly link to the database records from which the data can be accessed. In the main text, data citations are formatted as follows: "Data ref: Smith et al, 2001" or "Data ref: NCBI Sequence Read Archive PRJNA342805, 2017". In the Reference list, data citations must be labeled with "[DATASET]". A data reference must provide the database name, accession number/identifiers and a resolvable link to the landing page from which the data can be accessed at the end of the reference. Further instructions are available at: http://www.embopress.org/page/journal/14693178/authorguide#referencesformat

8) Regarding data quantification and statistics, please make sure that the number "n" for how many independent experiments were performed, their nature (biological versus technical replicates), the bars and error bars (e.g. SEM, SD) and the test used to calculate p-values is indicated in the respective figure legends (also for EV and Appendix figures). Please also check that all the p-values are explained in the legend, and that these fit to those shown in the figure. Please provide statistical testing where applicable. Please avoid the phrase 'independent experiment', but clearly state if these were biological or technical replicates. Please also indicate (e.g. with n.s.) if testing was performed, but the differences are not significant. In case n=2, please show the data as separate datapoints without error bars and statistics. See also: http://www.embopress.org/page/journal/14693178/authorguide#statisticalanalysis

If n<5, please show single datapoints for diagrams.

9) Please add scale bars of similar style and thickness to microscopic images, using clearly visible black or white bars (depending on the background). Please place these in the lower right corner of the images themselves. Please do not write on or near the bars in the image but define the size in the respective figure legend.

10) Please also note our reference format:
http://www.embopress.org/page/journal/14693178/authorguide#referencesformat

11) We updated our journal's competing interests policy in January 2022 and request authors to consider both actual and perceived competing interests. Please review the policy https://www.embopress.org/competing-interests and update your competing interests if necessary. Please name this section 'Disclosure and Competing Interests Statement' and put it after the Acknowledgements section.

12) We now use CRediT to specify the contributions of each author in the journal submission system. CRediT replaces the author contribution section. Please use the free text box to provide more detailed descriptions and do NOT provide your final manuscript text file with an author contributions section. See also our guide to authors:
https://www.embopress.org/page/journal/14693178/authorguide#authorshipguidelines

13) All Materials and Methods need to be described in the main text using our 'Structured Methods' format, which is required for all research articles. According to this format, the Methods section should include a Reagents and Tools Table (listing key

reagents, experimental models, software, and relevant equipment and including their sources and relevant identifiers), uploaded as separate file, and a Methods section in which we encourage the authors to describe their methods using a step-by-step protocol format with bullet points, to facilitate the adoption of the methodologies across labs. More information on how to adhere to this format as well as downloadable templates (.doc) for the Reagents and Tools Table can be found in our author guidelines (section 'Structured Methods'):

https://www.embopress.org/page/journal/14693178/authorguide#manuscriptpreparation

14) Please add up to 5 keywords to the manuscript and order the sections like this, using these names:
Title page - Abstract (not more than 175 words) - Keywords - Introduction - Results - Discussion - Methods - Data availability section - Acknowledgements (please include here also the funding information) - Disclosure and Competing Interests Statement - References - Figure legends - Expanded View Figure legends

15) Please make sure that all the funding information is also entered into the online submission system and that it is complete and similar to the one in the acknowledgement section of the manuscript text file.

We would also welcome the submission of cover suggestions, or motifs to be used by our Graphics Illustrator in designing a cover.

As part of the EMBO publication's Transparent Editorial Process, EMBO reports publishes online a Review Process File (RPF) to accompany accepted manuscripts. This File will be published in conjunction with your paper and will include the referee reports, your point-by-point response and all pertinent correspondence relating to the manuscript.

You are able to opt out of this by letting the editorial office know (emboreports@embo.org). If you do opt out, the Review Process File link will point to the following statement: "No Review Process File is available with this article, as the authors have chosen not to make the review process public in this case."

I look forward to seeing a revised version of your manuscript when it is ready. Please let me know if you have questions or comments regarding the revision.

Kind regards,

Achim Breiling

Achim Breiling
Senior Editor
EMBO Reports

Referee #1:

In this manuscript, Sanchez-Cisneros and colleagues, examine how tracheal cell adhesion to the ECM underneath the epidermis helps shape the tracheal system. They show that if cell-ECM adhesion is perturbed the development of the tracheal system and the epidermis is disrupted. They also detect protrusions extending from the dorsal trunk cells towards the ECM.

The work is novel, the figures are clear, and the questions are well addressed. However, I find that some of the claims are not completely supported by the data presented. I have some suggestions that will, I believe, clarify certain points.

Major comments

At the beginning of the results section as in the introduction the authors claim that "It is generally assumed that trunk displacement occurs due to tip cells pulling on the trunks so that they follow their path dorsally." This sentence is not referenced, and I do not know where it has been shown or proposed to be like this. In addition, the comparison with the ventral branches is also not referenced and the movie does not really show this. Forces generated by tracheal branch migration have been shown to drive intercalation (Caussinus E, Colombelli J, Affolter M. Tip-cell migration controls stalk-cell intercalation during Drosophila tracheal tube elongation. Curr Biol. 2008;18(22):1727-1734. doi:10.1016/j.cub.2008.10.062), but not dorsal trunk (DT) displacement.
However, to rule out the possibility that DT displacement and the phenotype observed in XXX is due to dorsal branch pulling forces, the authors should analyze what happens in the absence of dorsal branches (in condition of Dpp signalling inhibition as in punt mutants or Dad overexpression conditions).

I am concerned about the TEM observations. The authors claim they can identify tracheal cells by their lumen (Fig. 2 C'). However, at stage 15, the tracheal lumen should be clearly identifiable, and the interluminal DT space should be wider relative to the size of the cells. In this case, there is nothing telling us that we are not looking at a dorsal branch or lateral trunk cell. Furthermore, at embryonic stage 15, the tracheal lumen is filled with a chitin filament, which is not visible in these micrographs. Also, there is quite a lot of tissue detachment and empty spaces between cells, which might be a sign of problems in sample fixing. Better images and more accurate identification of dorsal trunk cells is necessary to support the claim that "These experiments revealed a novel anatomical contact between the epidermis and tracheal trunks".

Timelapse imaging of the protrusions in DT cells is done with frames every 4 minutes (Video S3). This is not enough to properly show cellular protrusions and the images do not really show interaction with the epidermis. Video S4 has a better time resolution but it is very short and only shows the cut moment. Video S4, shows the cut, but the reported (and quantified recoil) is not clear. Nevertheless, the results are noteworthy and should be further analysed. Provided these embryos survive, would it be possible to check if embryos after laser cutting will develop wavy DTs?

What happens to the larvae under the genetic conditions presented in Fig.S3? Do they reach pupal stages? Do these animals reach adult stages?

The kayak phenotypes are very interesting and perhaps the authors could explore them more. As in inhibition of adhesion to the ECM, kay mutants display wavy dorsal trunks. Do they have defective adhesion? Fos being a transcription factor, this is a possibility. The authors should at least discuss the kay phenotypes more extensively and present a suitable hypothesis for the phenotype.

Minor comments

Page 2 Line 9/10 The sentence "tracheal tubes branch and migrate over neighbouring tissues of different biochemical and mechanical properties to ventilate them." should be rewritten. Tracheal cells do not migrate over other tissues to ventilate them.

Page 2 Line 24/25 The sentence "It has been generally assumed that trunks reach the dorsal side of the embryo because of the pulling forces of dorsal branch migration." needs to be backed up by a reference.

Page 7 Line 32/23 In this sentence, the references are not related to dorsal closure "Similarly, the signals that regulate epidermal dorsal closure do not participate in tracheal development, or vice versa (Letizia et al., 2023; Reichman-Fried et al., 1994)."

Page 12 Line 1 "Muscles attach to epidermal tendon cells through a dense meshwork of ECM" this sentence must be referenced.

Fig. S1- Single channel images (A'-C' and A'-C') should be presented in grayscale.

Fig. S4- Single channel images (A'-D' and A'-D') should be presented in grayscale.

Significance

The findings shown in this manuscript shed light on the interactions and cooperation between two organs, the tracheal system and the epidermis. These interactions are mediated by cell-ECM contacts which are important for the correct morphogenesis of both systems. The strengths of the work lie on its novelty and live analysis of these interactions. However, its weaknesses are related to some claims not completely backed by the data, some technical issues regarding imaging and some over-interpreted conclusions.

This basic research work will be of interest to a broad cell and developmental biology community as they provide a functional advance on the importance of cell-ECM interactions for the morphogenesis of a tubular organ. It is of specific interest to the specialized field of tubulogenesis and tracheal morphogenesis.

Referee #2:

Summary: In this paper, the authors explore the relationships between two Drosophila tissues - the epidermis and tracheal dorsal trunk (DT) - that get dorsally displaced during mid-late embryogenesis. The show a nice temporal correlation between the movements of the epithelia during dorsal closure and DT displacement. They also show a correlation between the movement of an endogenously tagged version of collagen and the DT, suggesting that the ECM may contribute to this coordinated movement. Through high magnification TEM, they show that tracheal cells make direct contact with the subset of epithelial cells, known as tendon cells, that also serve as muscle attachment sites. In between these contact sites, tracheae are separated from the epithelia by the muscles. Furthermore, the TEMs and confocal imaging of tracheal cells expressing a membrane marker at these contact sites show that the tracheal cells are extending filopodia toward the tendon cells. The authors then explore how a

variety of perturbations to the ECM produced by the tendon and DT cells affect DT and epithelial movement. They find that expressing membrane-associated matrix metalloproteases (MMP1 or MMP2) in tendon cells as well as perturbations in integrin or integrin signaling components leads to delays in dorsal displacement as well as defective lengthening of the tracheal DT tubes. They find that defects in the association between the tracheal and epidermal ECM attachments affect dorsal displacement of the epidermis, disrupting dorsal closure.

Major comments: I like the goals of this paper testing the idea that the ECM plays important roles in the coordination of tissue placement, and I think they have good evidence of that from this study. However, I disagree with the conclusions of the authors that disrupting contact between DT and the tendon cells has no effect on DT dorsal displacement. DT tracheal positioning is clearly delayed; the fact that it takes a lot longer indicates that the ECM does affect the process. It's just that there are likely backup systems in place - clearly not as good since the tracheal tubes end up being the wrong length. It also seems important that the parts of the DT where the dorsal branches (DB) emanate are moving dorsally ahead of the intervening portions of the trachea. This suggests to me that the DB normally does contribute to DT dorsal displacement and that this activity may be what helps the DT eventually get into its final position. The authors should test whether the portions of the DT that contact the DB are under tension. If the DB migration is providing some dorsal pulling force on the DT, this may also contribute to the observed increases in DT length observed with the perturbations of the ECM between the tendon cells and the trachea - if tube lengthening is a consequence of the pulling forces that would be created by parts of the trachea moving dorsally ahead of the other parts. Here again, it would be good to test if the DT itself is under additional tension when the ECM is disrupted.

Minor comments: The authors need to do a much better job in the intro and in the discussion of citing the work of the people who made many of the original findings that are relevant to this study. Many citations are missing (especially in the introduction) or the authors cite their own review (which most people will not have read) for almost everything (especially in the discussion). This fails to give credit to decades of work by many other groups and makes it necessary for someone who would want to see the original work to first consult the review before they can find the appropriate reference. I know it saves space (and effort) but I think citing the original work is important.

Figure 7 is not a model. It is a cartoon depicting what they see with confocal and TEM images.

Significance

Overall, this study is one of the first to focus on how the ECM affects coordination of tissue placement. The coordination of tracheal movement with that of the epidermis is very nicely documented here and the observation that the trachea make direct contact with the tendon cells/muscle attachment sites is quite convincing. It is less clear from the data how exactly the cells of the trachea and the ECM are affected by the different perturbations of the ECM. It seems like this could be better done with immunostaining of ECM proteins (collagen-GFP?), cell type markers, and super resolution confocal imaging with combinations of these markers. What happens right at the contact site between the tendon cell and the trachea with the perturbation? I think that at the level of analysis presented here, this study would be most appropriate for a specialized audience working in the ECM or fly embryo development field.

Referee #3:

The manuscript by Sanchez-Cisneros et al provides a detailed description of the cellular interactions between cells of the Drosophila embryonic trachea and nearby tendon and epidermal cells. The researchers use a combination of genetic experiments, light sheet style live imaging and transmission electron microscopy. The live imaging is particularly clear and detailed, and reveals protruding cells. The results overall suggest that interactions mediated through the ECM contribute to development of trachea and dorsal closure of epidermis. One new aspect is the existence of dorsal trunk filipodia that are under tension and may impact tracheal morphogenesis through required integrin/ECM interactions.

Major comments:

- Are the key conclusions convincing?

Generally, the key conclusions are well supported by the data, and the movies are very impressive. Interactions between the cell types are clearly shown, as is the correlations in their development. However, some of the images are challenging to decipher for a non-expert in Drosophila trachea, especially the EM images, and some of the data is indirect or a bit weak.

The data related to failure of dorsal closure affecting trachea relies on one homozygous allele of one gene (kayak), and so this is somewhat weak evidence. Even though kay is not detected in trachea, there could be secondary effects of the mutation or another lesion on the mutant chromosome. The segments look a bit uneven in the mutant examples.
- Should the authors qualify some of their claims as preliminary or speculative, or remove them altogether?

Some of the experiments have low n values, especially in imaging experiments, so these may be more preliminary, but they are

in concordance with other data.
- Would additional experiments be essential to support the claims of the paper? Request additional experiments only where necessary for the paper as it is, and do not ask authors to open new lines of experimentation.

Higher n-values would substantiate the claims.
To strengthen the argument that dorsal closure affects trachea morphogenesis mechanically, the authors might consider using of a combination of kay mutant alleles or other mutant genes in this pathway to provide stronger evidence. Or they could try a rescue experiment in epidermis and trachea separately for the kay mutants.
- Are the suggested experiments realistic in terms of time and resources? It would help if you could add an estimated cost and time investment for substantial experiments.

Imaging data can take a while to obtain, but the genetic experiments could be done in a couple of months, and the authors should be able to obtain any needed lines within a few weeks.
- Are the data and the methods presented in such a way that they can be reproduced?

Generally, yes. For the deGrad experiments, it is not clear how the fluorescent intensity was normalized - was this against a reference marker?
- Are the experiments adequately replicated and statistical analysis adequate?

There are several experiments with low n values, so this could fall below statistical significance. For example, data shown in Fig 1G: n=3; Fig 4D n=4, n=3; Fig 6J n=4

Minor comments:

- Specific experimental issues that are easily addressable.

To make the TEM images more easily interpreted, it would be helpful to provide a fluorescent image of all the relevant cell types (especially trachea, epidermis, muscle, and tendon cells, plus segmental boundaries) labelled accordingly, so that reader can correlate them more easily with the TEM images. They might also include a schematic of an embryo to show where the TEM field of view is.

It is hard to be confident that the EM images reflect the cells they claim and that the filopodia are in fact that, at least for people not used to looking at these types of images.
- Are prior studies referenced appropriately?

yes
- Are the text and figures clear and accurate?

yes
- Do you have suggestions that would help the authors improve the presentation of their data and conclusions?

The writing could be revised to be a bit clearer. Since the results of the experiments do not support the initial hypothesis, I found it a bit confusing as I read along. It may help to introduce an alternative hypothesis earlier to make the paper more logical and easy to follow.
To be more specific, On page 3, the authors say they "show that dorsal trunk displacement is mechanically coupled to the remodelling of the epidermis" and also in the results comment that "With two opposing forces pulling the trunks other factors likely participate in their dorsal displacement, but so far these have remained unstudied." But that doesn't end up being what they find. The results from figure 5 and related interpretation on page 17 says "cell-ECM interactions are important for proper trunk morphology, but not for its displacement." So this was confusing to read and I would encourage the authors to frame the issues a bit differently in terms of tube morphogenesis.

Some minor presentation issues:

What orientation is the cross-sectional view in figure 1C and movie 1?
On page 12, the authors say the "Electron micrographs also suggested high filopodial activity" but activity suggests dynamics that are not clear from EM. This could be re-phrased.

Significance

- Describe the nature and significance of the advance (e.g. conceptual, technical, clinical) for the field.

The results of the paper are significant in that they characterize a mechanical interaction between two tissue types in development, which are linked by the extracellular matrix that sits between them.

It is not clear to me that this describes a "novel mechanism for tissue coordination" as stated in the abstract, but it does characterize this type of interaction in a detailed cellular way.
- Place the work in the context of the existing literature (provide references, where appropriate).

For specialists, the work identifies a novel protruding cell type in the fly embryonic trachea, and provides beautiful and detailed imaging data on tracheal development. The "wavy" trachea phenotype is also uncommon and very interesting, so this result could be linked to the few papers that also describe this phenotype and be built up.
- State what audience might be interested in and influenced by the reported findings.

As it stands, this is most interesting for a specialized audience because it requires some understanding of the development of this system in particular. As it characterizes this to a new level of detail, it could be influential to those in the field. Some addition clarification of the results and re-framing could make the manuscript more clear and interesting for non-specialists.

- Define your field of expertise with a few keywords to help the authors contextualize your point of view. Indicate if there are any parts of the paper that you do not have sufficient expertise to evaluate.

I work with Drosophila and have studied embryonic and adult cell types, although not trachea specifically. I am familiar with all the genetic techniques and imaging techniques used here.

Full Revision

Manuscript number: #RC-2025-02961
Corresponding author(s): Luis Daniel Ríos-Barrera

Comments on the revision plan in blue and bold

1. General Statements

We would like to thank the reviewers for their comments, we see great value in the suggestions they made to strengthen our work. We are glad to see that they are in general positive about the manuscript. In the following, we include a point-by-point response to their comments, which are in general consistent with each other.

2. Description of the planned revisions

Insert here a point-by-point reply that explains what revisions, additional experimentations and analyses are planned to address the points raised by the referees.

Reviewer #1 (Evidence, reproducibility and clarity (Required)):

In this manuscript, Sanchez-Cisneros and colleagues, examine how tracheal cell adhesion to the ECM underneath the epidermis helps shape the tracheal system. They show that if cell-ECM adhesion is perturbed the development of the tracheal system and the epidermis is disrupted. They also detect protrusions extending from the dorsal trunk cells towards the ECM. The work is novel, the figures are clear, and the questions are well addressed. However, I find that some of the claims are not completely supported by the data presented. I have some suggestions that will, I believe, clarify certain points.

Major comments

At the beginning of the results section as in the introduction the authors claim that "It is generally assumed that trunk displacement occurs due to tip cells pulling on the trunks so that they follow their path dorsally." This sentence is not referenced, and I do not know where it has been shown or proposed to be like this. In addition, the comparison with the ventral branches is also not referenced and the movie does not really show this. Forces generated by tracheal branch migration have been shown to drive intercalation (Caussinus E, Colombelli J, Affolter M. Tip-cell migration controls stalk-cell intercalation during Drosophila tracheal tube elongation. Curr Biol. 2008;18(22):1727-1734. doi:10.1016/j.cub.2008.10.062), but not dorsal trunk (DT) displacement.

We agree that dorsal trunk displacement has not been discussed in previous works, just the fact that tip-cell migration influences stalk cell intercalation. We will rephrase this sentence, stating that dorsal trunk displacement has not been studied.
Completed

However, to rule out the possibility that DT displacement and the phenotype observed in XXX is due to dorsal branch pulling forces, the authors should analyze what happens in the absence of dorsal branches (in condition of Dpp signalling inhibition as in punt mutants or Dad overexpression conditions).

This is a great idea, and we thank the reviewer for suggesting this. We tried to achieve a similar goal by expressing a Dominant Negative FGFR (Breathless-DN) in the tracheal system, since its expression under *btl-gal4* affects tip cell migration. However, the phenotype arises too late to have an effect in dorsal branch migration during the stages we were interested in analyzing.

The alternative proposed by the reviewer should be more efficient, as blocking Dpp signalling prevents the formation of dorsal branches completely. We have just received flies carrying the UAS-Dad construct. We will express Dad under *btl-gal4* and see how this affects dorsal trunk displacement.

Completed

I am concerned about the TEM observations. The authors claim they can identify tracheal cells by their lumen (Fig. 2 C'). However, at stage 15, the tracheal lumen should be clearly identifiable, and the interluminal DT space should be wider relative to the size of the cells. In this case, there is nothing telling us that we are not looking at a dorsal branch or lateral trunk cell. Furthermore, at embryonic stage 15, the tracheal lumen is filled with a chitin filament, which is not visible in these micrographs. Also, there is quite a lot of tissue detachment and empty spaces between cells, which might be a sign of problems in sample fixing. Better images and more accurate identification of dorsal trunk cells is necessary to support the claim that "These experiments revealed a novel anatomical contact between the epidermis and tracheal trunks".

The protocol that we use for TEM involves performing 1-µm sections that allow us to stage embryos and to identify the anatomical regions using light microscopy and then switch to ultra-thin sections for electron microscopy once we have found the right position within the sample. This approach also allows us to determine the integrity of the sample. We attach here a micrograph of the last section we analyzed before we decided to do the EM analysis. The asterisk (*) points to a region where the multicellular lumen of the trunk is visible. Due to its proximity to the posterior spiracles, we are confident this is the dorsal trunk and not the lateral trunk. We realize now, after comparing this image with an atlas of development (Campos-Ortega and Hartenstein, 2013), that the stage we chose to illustrate the interaction is a stage 14 embryo instead of the stage 15 we indicated in the manuscript. We will change the stage but given that dorsal closure has already started by stage 14, this does not affect our analysis. Still, we apologize for the mis-staging of the embryo.

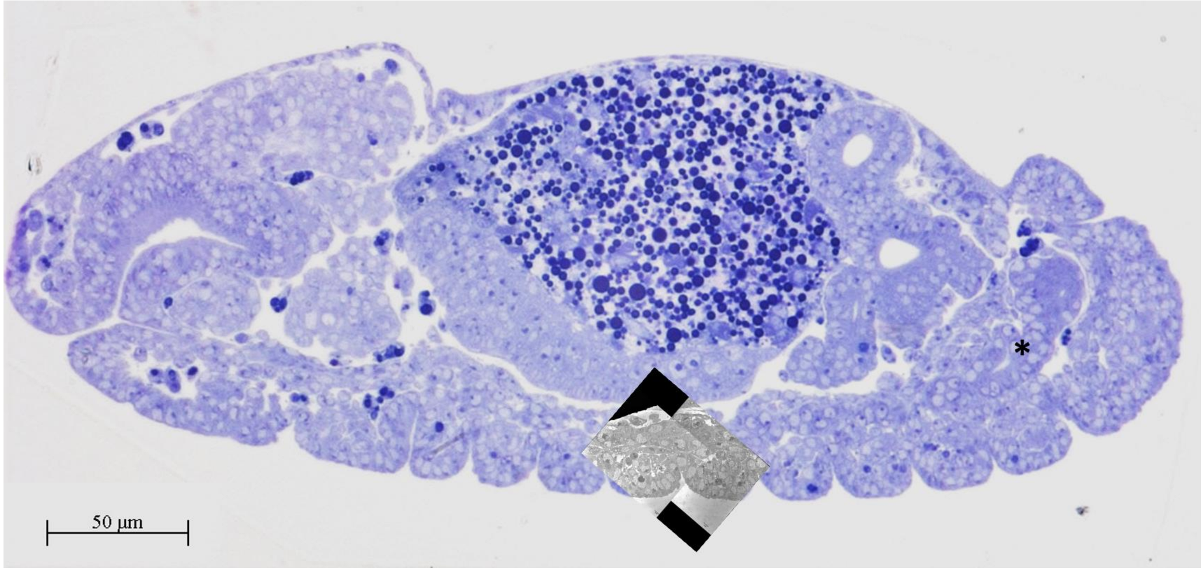

In the light-microscopy image, we have overlaid the EM section to the corresponding region of interest. We agree that the lumen should be thicker compared to the length of the cells, if the section would be cutting the trunk through its largest diameter. However, the protrusions we see do not emerge from the middle part of the trunk where the lumen is found but are seen towards the dorsal side of the trunk, where the lumen will no longer be visible in a longitudinal section as the ones we present. In the embryo shown in Figure 2A-C, our interpretation is that the section was done through a very shallow section of the lumen (represented below). We interpret this from the fact that we see abundant electron-dense areas which we think are adherens junctions from multiple cells. These junctions are visible in Figure 2C but are currently not labelled. We will add arrows to increase their visibility:

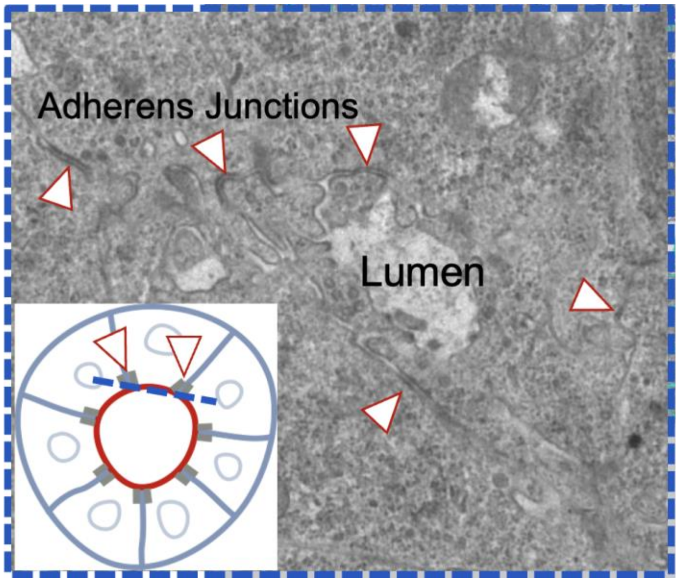

Given that protruding cells lie at the base of dorsal branches, it would be expected that in some sections we would find the protrusions close to the dorsal branches. This is in fact what we show in the micrograph shown in Figure 2D, with a lower magnification overview image shown in Figure S2D. In this case, we see a cell in close proximity to the tendon cells on one side (Figure 2D), which is connected to a dorsal branch on the opposite side (shown in Figure S2D). This dorsal branch is clearly autocellular and chitin deposition is visible as expected for the developmental stage. Again, in Figure S2E we see an electron-dense patch near the lumen that corresponds to the adherens junctions that seal the lumen. We see that all this needs to be better explained in the manuscript, so we will elaborate on the descriptions, and incorporate the light microscopy micrograph to the supplemental figures. This should also aid with the anatomical descriptions requested by Reviewer #3. Nevertheless, we think these observations confirm that what we are describing are the contact points between the dorsal trunk and tendon cells.

Completed. We added the bright field reference image and arrows to describe with more detail certain images (Figure 2A'; 2C).

Timelapse imaging of the protrusions in DT cells is done with frames every 4 minutes (Video S3). This is not enough to properly show cellular protrusions and the images do not really show interaction with the epidermis. Video S4 has a better time resolution but it is very short and only shows the cut moment. Video S4, shows the cut, but the reported (and quantified recoil) is not clear. Nevertheless, the results are noteworthy and should be further analysed.

We will acquire high temporal resolution time-lapse images using E-Cadherin::GFP and *btl-gal4, UAS*-PH::mCherry to show the behaviour of the protrusions on a short time scale.

Completed. We realized it was more useful to use markers for actin (Utr::GFP) and for membrane (CD4::mIFP) to show how dynamic the protrusions were. Acquisition was done every 30 seconds.

Provided these embryos survive, would it be possible to check if embryos after laser cutting will develop wavy DTs?

We think it would be interesting to carry out this experiment, but the laser cut experiments were done under a collaborative visit and we would not be able to repeat it in a short-term period.

What happens to the larvae under the genetic conditions presented in Fig.S3? Do they reach pupal stages? Do these animals

reach adult stages?

We have seen escapers out of these crosses, but we have not quantified the lethality of the experiment. We will analyse this and include it in the manuscript.

Completed. We quantified the viability to adults in the experiments where we knocked down βIntegrin using the deGradFP system.

The kayak phenotypes are very interesting and perhaps the authors could explore them more. As in inhibition of adhesion to the ECM, kay mutants display wavy dorsal trunks. Do they have defective adhesion? Fos being a transcription factor, this is a possibility. The authors should at least discuss the kay phenotypes more extensively and present a suitable hypothesis for the phenotype.

We agree that the *kayak* experiments might bring more consequences than just preventing dorsal closure. We will complement this approach by blocking dorsal closure by other independent means. We will use *pannier-gal4* (a lateral epidermis driver), *engrailed-gal4* (a driver for epidermal posterior compartment), and *332-gal4* (an amnioserosa driver) to express dominant-negative Moesin. In our experience, this also delays dorsal closure and it should result in a similar tracheal phenotype as the one we see in *kayak* embryos.

Completed. We carried out the experiments using *en-gal4, UAS-mCherry.* We used 332-gal4 and pnr-gal4 to express Moesin-DN and observed similar results, but since these had any fluorescent reporters associated to the driver, we decided not to include them in the work. We also complemented these experiments with HCR-FISH detection of *kay* transcripts to corroborate that *kay* is not expressed in the tracheal system during the stages we are studying.

Minor comments

Page 2 Line 9/10 The sentence "tracheal tubes branch and migrate over neighbouring tissues of different biochemical and mechanical properties to ventilate them." should be rewritten. Tracheal cells do not migrate over other tissues to ventilate them.

We meant to say that tracheal cells migrate over other tissues at the same time as they branch and interconnect to allow gas exchange in their surroundings after tracheal morphogenesis is completed. Ventilation is used here as a synonym for gas exchange or breathing. We will rephrase this if the reviewer considers it confusing.

Completed.

Page 2 Line 24/25 The sentence "It has been generally assumed that trunks reach the dorsal side of the embryo because of the pulling forces of dorsal branch migration." needs to be backed up by a reference.

As explained above, we will rephrase this sentence.

Completed.

Page 7 Line 32/23 In this sentence, the references are not related to dorsal closure "Similarly, the signals that regulate epidermal dorsal closure do not participate in tracheal development, or vice versa (Letizia et al., 2023; Reichman-Fried et al., 1994)."

Our goal in this sentence was to explain that while JNK is required for proper epidermal dorsal closure, loss of JNK signaling in the trachea does not affect tracheal development (as shown by Letizia et al., 2023). At the same time, Reichman-Fried et al., 1994 described the phenotypes of loss of *breathless (btl)*. We will remove this last reference as the work does not study the epidermis. We will rephrase the sentence as: “Similarly, the signals that regulate epidermal dorsal closure do not participate in tracheal development; namely, JNK signaling (Letizia et al., 2023).”

Full Revision

Completed.

Page 12 Line 1 "Muscles attach to epidermal tendon cells through a dense meshwork of ECM" this sentence must be referenced.

We will add the corresponding references for this statement: (Fogerty et al., 1994; Prokop et al., 1998; Urbano et al., 2009). We will change “dense” for “specialized”.

Completed.

Fig. S1- Single channel images (A'-C' and A'-C') should be presented in grayscale.

Fig. S4- Single channel images (A'-D' and A'-D') should be presented in grayscale.

We will add the grayscale, single-channel images for these figures.

Completed.

Reviewer #1 (Significance (Required)):

The findings shown in this manuscript shed light on the interactions and cooperation between two organs, the tracheal system and the epidermis. These interactions are mediated by cell-ECM contacts which are important for the correct morphogenesis of both systems. The strengths of the work lie on its novelty and live analysis of these interactions. However, its weaknesses are related to some claims not completely backed by the data, some technical issues regarding imaging and some over-interpreted conclusions.

This basic research work will be of interest to a broad cell and developmental biology community as they provide a functional advance on the importance of cell-ECM interactions for the morphogenesis of a tubular organ. It is of specific interest to the specialized field of tubulogenesis and tracheal morphogenesis.

Reviewer #2 (Evidence, reproducibility and clarity (Required)):

Summary: In this paper, the authors explore the relationships between two Drosophila tissues - the epidermis and tracheal dorsal trunk (DT) - that get dorsally displaced during mid-late embryogenesis. The show a nice temporal correlation between the movements of the epithelia during dorsal closure and DT displacement. They also show a correlation between the movement of an endogenously tagged version of collagen and the DT, suggesting that the ECM may contribute to this coordinated movement. Through high magnification TEM, they show that tracheal cells make direct contact with the subset of epithelial cells, known as tendon cells, that also serve as muscle attachment sites. In between these contact sites, tracheae are separated from the epithelia by the muscles. Furthermore, the TEMs and confocal imaging of tracheal cells expressing a membrane marker at these contact sites show that the tracheal cells are extending filopodia toward the tendon cells. The authors then explore how a variety of perturbations to the ECM produced by the tendon and DT cells affect DT and epithelial movement. They find that expressing membrane-associated matrix metalloproteases (MMP1 or MMP2) in tendon cells as well as perturbations in integrin or integrin signaling components leads to delays in dorsal displacement as well as defective lengthening of the tracheal DT tubes. They find that defects in the association between the tracheal and epidermal ECM attachments affect dorsal displacement of the epidermis, disrupting dorsal closure.

Major comments: I like the goals of this paper testing the idea that the ECM plays important roles in the coordination of tissue placement, and I think they have good evidence of that from this study. However, I disagree with the conclusions of the authors that disrupting contact between DT and the tendon cells has no effect on DT dorsal displacement. DT tracheal

positioning is clearly delayed; the fact that it takes a lot longer indicates that the ECM does affect the process. It's just that there are likely backup systems in place - clearly not as good since the tracheal tubes end up being the wrong length.

We agree with this view; in our deGradFP experiments we see a delayed DT displacement. We focused our analyses on the coordination with epidermal remodelling, which remained unaltered, but we in fact see a delayed progression in dorsal displacement of both tissues (Figure 5I-J). We will emphasize this in the corresponding section of the Results.
Completed.

It also seems important that the parts of the DT where the dorsal branches (DB) emanate are moving dorsally ahead of the intervening portions of the trachea. This suggests to me that the DB normally does contribute to DT dorsal displacement and that this activity may be what helps the DT eventually get into its final position. The authors should test whether the portions of the DT that contact the DB are under tension. If the DB migration is providing some dorsal pulling force on the DT, this may also contribute to the observed increases in DT length observed with the perturbations of the ECM between the tendon cells and the trachea - if tube lengthening is a consequence of the pulling forces that would be created by parts of the trachea moving dorsally ahead of the other parts. Here again, it would be good to test if the DT itself is under additional tension when the ECM is disrupted.

We thank the reviewer for the suggested experiments. We agree with the fact that the dorsal branches should pull on the dorsal trunk and that this interaction should generate tension. Unfortunately, we are unable to test this with the experiments proposed by the reviewer, but we propose an alternative strategy to overcome this. We understand that the reviewer suggests we do laser cut experiments in dorsal branches to see if there is a recoil in the opposite direction of dorsal branch migration. We carried out our laser cut experiments using a 2-photon laser through a visit to the EMBL imaging facility, using funds from a collaborative grant. Funding a second visit would require us to apply for extra funding, which would delay the preparation of the experiments. We are aware of UV-laser setups within our university, however, UV-laser cuts would also affect the epidermis above the dorsal branches, which we think might contribute to recoil we would expect to see.

Instead of doing laser cuts, we have designed an experiment based on the suggestion of reviewer #1 of blocking Dpp signaling (with UAS-Dad), which would prevent the formation of dorsal branches. We expect that in this experimental setup, the trunk will bend ventrally in response to the pulling forces of the ventral branches. We will also co-express UAS-Dad (to prevent dorsal branch formation) and UAS-Mmp2 (to 'detach' the dorsal trunk from the epidermis), and we would expect to at least partially rescue the wavy trunk phenotype.
Completed. We would like to add that laser cuts had been previously done in dorsal branches (Caussinus et al., 2008). This was carried out to see how pulling forces from terminal cells affect stalk cell intercalation. This work confirmed that tip cells can pull on stalk cells and these forces likely are transmitted to the trunks (as our new experiments also show). Unfortunately, this work did not measure immediate recoil speed so we cannot confirm the tension generated in the trunk.

Minor comments: The authors need to do a much better job in the intro and in the discussion of citing the work of the people who made many of the original findings that are relevant to this study. Many citations are missing (especially in the introduction) or the authors cite their own review (which most people will not have read) for almost everything (especially in the discussion). This fails to give credit to decades of work by many other groups and makes it necessary for someone who would want to see the original work to first consult the review before they can find the appropriate reference. I know it saves space (and effort) but I think citing the original work is important.

The reviewer is right; we apologize for falling into this practice. We will reference the original works wherever it is needed.
Completed.

Figure 7 is not a model. It is a cartoon depicting what they see with confocal and TEM images.

We will change the figure; we will include our interpretations of the phenotypes we observed under different experimental manipulations.

Completed.

Reviewer #2 (Significance (Required)):

Overall, this study is one of the first to focus on how the ECM affects coordination of tissue placement. The coordination of tracheal movement with that of the epidermis is very nicely documented here and the observation that the trachea make direct contact with the tendon cells/muscle attachment sites is quite convincing. It is less clear from the data how exactly the cells of the trachea and the ECM are affected by the different perturbations of the ECM. It seems like this could be better done with immunostaining of ECM proteins (collagen-GFP?), cell type markers, and super resolution confocal imaging with combinations of these markers. What happens right at the contact site between the tendon cell and the trachea with the perturbation? I think that at the level of analysis presented here, this study would be most appropriate for a specialized audience working in the ECM or fly embryo development field.

Reviewer #3 (Evidence, reproducibility and clarity (Required)):

Summary

The manuscript by Sanchez-Cisneros et al provides a detailed description of the cellular interactions between cells of the Drosophila embryonic trachea and nearby tendon and epidermal cells. The researchers use a combination of genetic experiments, light sheet style live imaging and transmission electron microscopy. The live imaging is particularly clear and detailed, and reveals protruding cells. The results overall suggest that interactions mediated through the ECM contribute to development of trachea and dorsal closure of epidermis. One new aspect is the existence of dorsal trunk filipodia that are under tension and may impact tracheal morphogenesis through required integrin/ECM interactions.

Major comments:

- Are the key conclusions convincing?

Generally, the key conclusions are well supported by the data, and the movies are very impressive. Interactions between the cell types are clearly shown, as is the correlations in their development. However, some of the images are challenging to decipher for a non-expert in Drosophila trachea, especially the EM images, and some of the data is indirect or a bit weak.

We thank the reviewer for their observations. As mentioned above in response to Reviewer #1, we will add an overview image of the embryo we processed for TEM that is presented in Figure 2.

Completed.

The data related to failure of dorsal closure affecting trachea relies on one homozygous allele of one gene (kayak), and so this is somewhat weak evidence. Even though kay is not detected in trachea, there could be secondary effects of the mutation or another lesion on the mutant chromosome. The segments look a bit uneven in the mutant examples.

The reviewer is right; as we proposed before, we will complement the *kayak* experiments with independent approaches that will delay dorsal closure.

Completed.

- Should the authors qualify some of their claims as preliminary or speculative, or remove them altogether?

Some of the experiments have low n values, especially in imaging experiments, so these may be more preliminary, but they are in concordance with other data.

The problem we face in our live-imaging experiments is related to the probability of finding the experimental embryos. In most of our experiments we combine double-tissue labelling plus the expression of genetic tools. This generally corresponds to a very small proportion of the progeny. We will aim to have at least 4 embryos per condition.

Completed.

- Would additional experiments be essential to support the claims of the paper? Request additional experiments only where necessary for the paper as it is, and do not ask authors to open new lines of experimentation.

Higher n-values would substantiate the claims.

To strengthen the argument that dorsal closure affects trachea morphogenesis mechanically, the authors might consider using of a combination of kay mutant alleles or other mutant genes in this pathway to provide stronger evidence. Or they could try a rescue experiment in epidermis and trachea separately for the kay mutants.

We think our experiments delaying dorsal closure using the Gal4/UAS system and a variety of drivers should address the point of the possible indirect effects of *kay* in tracheal development.

Completed.

- Are the suggested experiments realistic in terms of time and resources? It would help if you could add an estimated cost and time investment for substantial experiments.

Imaging data can take awhile to obtain, but the genetic experiments could be done in a couple of months, and the authors should be able to obtain any needed lines within a few weeks.

The reviewer is correct, we will be able to plan our crosses for the proposed experiments within a couple of months.

- Are the data and the methods presented in such a way that they can be reproduced?

Generally, yes. For the deGrad experiments, it is not clear how the fluorescent intensity was normalized - was this against a reference marker?

Briefly, we used signals from within the embryo as internal controls. In the case of *en-gal4*, we normalized the signal to the sections of the embryo where *en* is not expressed and therefore, beta-integrin levels should not be affected. In the case of *btl-gal4*, we normalized against the signal surrounding the trunks which should also not be affected by the deGradFP system. We will elaborate on these analyses in the methods section.

Completed.

- Are the experiments adequately replicated and statistical analysis adequate?

There are several experiments with low n values, so this could fall below statistical significance. For example, data shown in Fig 1G: n=3; Fig 4D n=4, n=3; Fig 6J n=4

As mentioned above, we will increase our sample sizes.

Completed.

Minor comments:

- Specific experimental issues that are easily addressable.

To make the TEM images more easily interpreted, it would be helpful to provide a fluorescent image of all the relevant cell types (especially trachea, epidermis, muscle, and tendon cells, plus segmental boundaries) labelled accordingly, so that reader can correlate them more easily with the TEM images. They might also include a schematic of an embryo to show where the TEM field of view is.

We believe this should be addressed by adding the light microscopy section of the embryo with the TEM image overlaid as illustrated above.

Completed.

It is hard to be confident that the EM images reflect the cells they claim and that the filopodia are in fact that, at least for people not used to looking at these types of images.

As we explained in the response to Reviewer #1, we will elaborate on the descriptions of our TEM data. We think that adding the reference micrograph will aid with the interpretations of the TEM images.
Completed.

- Are prior studies referenced appropriately?
yes
- Are the text and figures clear and accurate?
yes

- Do you have suggestions that would help the authors improve the presentation of their data and conclusions?
The writing could be revised to be a bit clearer. Since the results of the experiments do not support the initial hypothesis, I found it a bit confusing as I read along. It may help to introduce an alterative hypothesis earlier to make the paper more logical and easy to follow.
To be more specific, On page 3, the authors say they "show that dorsal trunk displacement is mechanically coupled to the remodelling of the epidermis" and also in the results comment that "With two opposing forces pulling the trunks other factors likely participate in their dorsal displacement, but so far these have remained unstudied." But that doesn't end up being what they find. The results from figure 5 and related interpretation on page 17 says "cell-ECM interactions are important for proper trunk morphology, but not for its displacement." So this was confusing to read and I would encourage the authors to frame the issues a bit differently in terms of tube morphogenesis.

We see how this might be confusing. We will rewrite the introduction so that the work is easier to follow. To achieve this, we will state from the beginning the mechanisms we anticipate that regulate trunk displacement: 1) adhesion to the epidermis, 2) pulling forces from the dorsal branches and 3) a combination of both.
Completed.

Some minor presentation issues:
What orientation is the cross-sectional view in figure 1C and movie 1?

We will add a dotted box that indicates the region that we turned 90° to show the cross-section.
We drew a doted box in the region of the embryo that corresponds to the cross-section but this difficulted the visibility of important landmarks of the embryo. Instead, we now describe in the figure legend the region that corresponds in the embryo with respect to the dorsal branches: "Cross-section was done between the 5th and 6th branch from anterior to posterior with respect to (A)"

On page 12, the authors say the "Electron micrographs also suggested high filopodial activity" but activity suggests dynamics that are not clear from EM. This could be re-phrased.

As the reviewer indicates, we cannot conclude dynamics from a static image. We will replace "suggested high filopodial activity" with "revealed filopodial abundance".
Completed.

Reviewer #3 (Significance (Required)):

Full Revision

- Describe the nature and significance of the advance (e.g. conceptual, technical, clinical) for the field.
The results of the paper are significant in that they characterize a mechanical interaction between two tissue types in development, which are linked by the extracellular matrix that sits between them.
It is not clear to me that this describes a "novel mechanism for tissue coordination" as stated in the abstract, but it does characterize this type of interaction in a detailed cellular way.

- Place the work in the context of the existing literature (provide references, where appropriate).
For specialists, the work identifies a novel protruding cell type in the fly embryonic trachea, and provides beautiful and detailed imaging data on tracheal development. The "wavy" trachea phenotype is also uncommon and very interesting, so this result could be linked to the few papers that also describe this phenotype and be built up.

- State what audience might be interested in and influenced by the reported findings.
As it stands, this is most interesting for a specialized audience because it requires some understanding of the development of this system in particular. As it characterizes this to a new level of detail, it could be influential to those in the field. Some addition clarification of the results and re-framing could make the manuscript more clear and interesting for non-specialists.

- Define your field of expertise with a few keywords to help the authors contextualize your point of view. Indicate if there are any parts of the paper that you do not have sufficient expertise to evaluate.
I work with Drosophila and have studied embryonic and adult cell types, although not trachea specifically. I am familiar with all the genetic techniques and imaging techniques used here.

3. Description of the revisions that have already been incorporated in the transferred manuscript

Please insert a point-by-point reply describing the revisions that were <u>already carried out and included</u> in the transferred manuscript. If no revisions have been carried out yet, please leave this section empty.

4. Description of analyses that authors prefer not to carry out

Please include a point-by-point response explaining why some of the requested data or additional analyses <u>might not be necessary or cannot be provided within the scope of a revision</u>. This can be due to time or resource limitations or in case of disagreement about the necessity of such additional data given the scope of the study. Please leave empty if not applicable.

We will incorporate most of the suggestions of the reviewers, which we find very constructive for our manuscript. The two exceptions were already mentioned above but are summarized below:

- As mentioned above, we will not be able to carry out additional laser cut experiments, but with the genetic experiments we intend to do, we think we can partly address the same issues that the laser cut experiments would answer.
- Regarding electron microscopy, we think that incorporating the wide-field images and our detailed descriptions of the data will improve the clarity of our interpretations.

Dear Dr. Rios-Barrera,

Thank you for the submission of your revised manuscript to our editorial offices. I have now received the reports from the three referees that I asked to re-evaluate the study, you will find below. As you will see, the referees now support publication of your study in EMBO reports. Referees #2 and #3 have dome final comments and suggestions to improve the manuscript, I ask you to address in a final revised manuscript. Please also provide a detailed final p-b-p-response regarding the remaining referee points and the editorial requests below.

Editorial requests:

- Please have your final manuscript carefully proofread by a native speaker. There are still typos, grammatical error or unusual phrases in the present manuscript text (see also the comment by referee #3).

- Please provide a .docx formatted version of the final manuscript text (including legends for main figures and EV figures), but without the figures included.

- The format of the author list in the manuscript text file needs to be the same as in the submission system (First Name; Last Name). Please change this.

- Please upload individual production quality figure files as .eps, .tif, .jpg (one file per figure), of main figures and EV figures. Please upload these as separate, individual files upon re-submission. These should not be part of the final manuscript text file.

- The Expanded View format, which will be displayed in the main HTML of the paper in a collapsible format, has replaced the Supplementary information. Please upload the 5 Appendix figures as separate EV figures. Please follow the nomenclature Figure EV1, Figure EV2 etc. The figure legend for these should be included in the main manuscript document file in a section called Expanded View Figure Legends after the main Figure Legends section (see below).

- Please add up to five keywords to the manuscript and order the manuscript sections like this, using only these names:
Title page - Abstract - Keywords - Introduction - Results - Discussion - Methods - Data availability section - Acknowledgements - Disclosure and Competing Interests Statement - References - Figure legends - Expanded View Figure legends

- We updated our journal's competing interests policy in January 2022 and request authors to consider both actual and perceived competing interests. Please name this section 'Disclosure and Competing Interests Statement' and put it after the Acknowledgements section.
See: https://link.springer.com/partners/embo-press/editorial-policies#Competing%20interest%20disclosures

- We now use CRediT to specify the contributions of each author in the journal submission system. CRediT replaces the author contribution section. Please use the free text box to provide more detailed descriptions and do NOT provide your final manuscript text file with an author contributions section. See also our guide to authors (section 'Author contributions'): https://link.springer.com/journal/44319/submission-guidelines#cms-Revised-submissions

- Please check again that the number "n" for how many independent experiments were performed, their nature (biological versus technical replicates), the bars and error bars (e.g. SEM, SD) and the test used to calculate p-values is indicated in the respective figure legends (main and EV figures). Please also check that all the p-values are explained in the legend, and that these fit to those shown in the figure. Please provide statistical testing where applicable. Please avoid the phrase 'independent experiment' but clearly state if these were biological or technical replicates. Please also indicate (e.g. with n.s.) if testing was performed, but the differences are not significant. In case n=2, please show the data as separate datapoints without error bars and statistics. See also:

https://link.springer.com/journal/44319/submission-guidelines#cms-Figure-and-data-presentation

If n<5, please show single datapoints for diagrams. Moreover:
- Please define the annotated p values ****/***/**/* as well as provide the exact p-values for the same in the legend of figure 3G, 4D, S3 C, S4 E, F; 5H, J; 6J, 7F as appropriate.
- Please note that information related to n is missing in the legends of figures S1 B, S5 C
- Please note that the error bars are not defined in the legend of figure S1 B.
- Please note that the scale bar needs to be defined for figures 4A', B'; S3 A, 5A', B', D', E'; 6A', B', D', E', F'; S5 A'; 7A', B, C'

- Please add scale bars of similar style and thickness to all microscopic images, using clearly visible black or white bars (depending on the background). Please place these in the lower right corner of the images themselves. Please do not write on or near the bars in the image but define the size in the respective figure legend. Presently, all scale bars have text nearby.

- Please make sure that each final figure panel is called out separately and that the panels are called out sequentially. Presently, it seems there are no separate callouts for panels 1C, 2C, 7ABE and 8ABCDE. Please check. Moreover, please add callouts for the movies (see below).

- The correct nomenclature for movie files is Movie EVx. Place change this in all places and add callouts for each file in the manuscript text. Please also provide legends for each movie. Each legend needs to be provided as separate text file and then uploaded, ZIPped together with its corresponding movie (so that we have 9 ZIP folders uploaded). Do not provide movie legends in the main text file.

- Please make sure that all the funding information is also entered into the online submission system and that it is complete and similar to the one in the acknowledgement section of the manuscript text file. Presently, the grant number #IA202923 is provided in the manuscript text file, while IN203525 is entered in the submission system. Please check.

- Thanks for providing the source data. Please upload the numerical source data directly to the manuscript files, as one folder per main figure, grouping together the separate files (in excel format) for the panels with numerical data, and ZIPed together. Please make sure that the deposited image source data is public latest upon online publication of the paper.

In addition, I would need from you uploaded separately:
- a short, two-sentence summary of the manuscript (not more than 35 words).
- two to four short (!) bullet points highlighting the key findings of your study (two lines each).
- a schematic summary figure as separate file that provides a sketch of the major findings (not a data image) in jpeg or tiff format (with the exact width of 550 pixels and a height of not more than 400 pixels) that can be used as a visual synopsis on our website.

I look forward to seeing the further revised version of your manuscript when it is ready. Please let me know if you have questions regarding the revision.

Best,

Achim Breiling
Senior Editor
EMBO Reports

Referee #1:

The authors have performed all the experiments requested, have clarified my doubts and answered to all my concerns. The manuscript has improved substantially since it was first submitted to Review Commons.

Referee #2:

This paper characterizes interactions between cells of the Drosophila embryonic trachea and nearby tendon and epidermal cells through pulling on a nearby ECM. The advance live imaging combined with electron microscopy is impressive and makes for a very detailed and valuable description, which is complemented by genetic experiments. The revised manuscript has been improved in clarity and benefitted from some additional, supporting genetic experiments and descriptions, which have strengthened the authors' arguments. Some n values remain a bit small, and the p values are presented with an unnecessary number of significant digits, so these could be improved. That said, all in all, the data are convincing and support the interesting idea that the ECM is mechanically coupling the development of several tissues. The manuscript will be of significant interest to those in the field and is worthy of being accepted for publication.

Referee #3:

The authors did an excellent job addressing the concerns raised by all three reviewers and I would be delighted to see the paper published. My minor concerns are just small edits: Page 2, line 12, remove the comma after "to". Page 2, line 24 after "dorsally" use a ";" not a comma. Page 2, line 33, add a comma after "this". Page 4, line 5, add a comma after "development". Page 5, line 29, add the words "with antiserum" after embryos. Page 10, line three: the correct word is interface (not interphase). The authors should carefully check the rest of the manuscript for similar mistakes. I think I got too involved in thinking about the experiments to be as good about editing after a while.

Point-by-point response
Manuscript number: EMBOR-2025-62287V3
Corresponding author(s): Luis Daniel Ríos-Barrera

Dear Dr. Rios-Barrera,

Thank you for the submission of your revised manuscript to our editorial offices. I have now received the reports from the three referees that I asked to re-evaluate the study, you will find below. As you will see, the referees now support publication of your study in EMBO reports. Referees #2 and #3 have dome final comments and suggestions to improve the manuscript, I ask you to address in a final revised manuscript. Please also provide a detailed final p-b-p-response regarding the remaining referee points and the editorial requests below.

Editorial requests:

- Please have your final manuscript carefully proofread by a native speaker. There are still typos, grammatical error or unusual phrases in the present manuscript text (see also the comment by referee #3).

As requested by the editorial team and Referee #3, the manuscript has been thoroughly proofread. We have corrected all spelling and grammatical errors and rephrased several sentences to ensure clarity and flow.

- Please provide a .docx formatted version of the final manuscript text (including legends for main figures and EV figures), but without the figures included.

We have uploaded the final manuscript file and the figures as separate files, as requested.

- The format of the author list in the manuscript text file needs to be the same as in the submission system (First Name; Last Name). Please change this.

The author list has been updated to match the format in the submission system (First Name; Last Name).

- Please upload individual production quality figure files as .eps, .tif, .jpg (one file per figure), of main figures and EV figures. Please upload these as separate, individual files upon re-submission. These should not be part of the final manuscript text file.

We have uploaded all main and EV figures as individual .jpg files. These figures have been removed from the final manuscript text file.

- The Expanded View format, which will be displayed in the main HTML of the paper in a collapsible format, has replaced the Supplementary information. Please upload the 5 Appendix figures as separate EV figures. Please follow the nomenclature Figure EV1, Figure EV2 etc. The figure legend for these should be included in the main manuscript document file in a section called Expanded View Figure Legends after the main Figure Legends section (see below).

We have renamed the referred figures to Figure EV1-EV5, and uploaded them as separate files. We included the EV Figure Legends in the main manuscript after the main figure legends.

- Please add up to five keywords to the manuscript and order the manuscript sections like this, using only these names:
Title page - Abstract - Keywords - Introduction - Results - Discussion - Methods - Data availability section - Acknowledgements - Disclosure and Competing Interests Statement - References - Figure legends - Expanded View Figure legends

We have included five keywords and reordered the manuscript sections as requested.

- We updated our journal's competing interests policy in January 2022 and request authors to consider both actual and perceived competing interests. Please name this section 'Disclosure and Competing Interests Statement' and put it after the Acknowledgements section.
See: https://link.springer.com/partners/embo-press/editorial-policies#Competing%20interest%20disclosures

We have updated the “Disclosure and Competing Interests Statement” section and moved it after the Acknowledgements.

- We now use CRediT to specify the contributions of each author in the journal submission system. CRediT replaces the author contribution section. Please use the free text box to provide more detailed descriptions and do NOT provide your final manuscript text file with an author contributions section. See also our guide to authors (section 'Author contributions'):
https://link.springer.com/journal/44319/submission-guidelines#cms-Revised-submissions
Author contributions were removed from the manuscript and added into the system.

- Please check again that the number "n" for how many independent experiments were performed, their nature (biological versus technical replicates), the bars and error bars (e.g. SEM, SD) and the test used to calculate p-values is indicated in the respective figure legends (main and EV figures). Please also check that all the p-values are explained in the legend, and that these fit to those shown in the figure. Please provide statistical testing where applicable. Please avoid the phrase 'independent experiment' but clearly state if these were biological or technical replicates. Please also indicate (e.g. with n.s.) if testing was performed, but the differences are not significant. In case n=2, please show the data as separate datapoints without error bars and statistics. See also:
https://link.springer.com/journal/44319/submission-guidelines#cms-Figure-and-data-presentation

If n<5, please show single datapoints for diagrams. Moreover:
- Please define the annotated p values ****/***/**/* as well as provide the exact p-values for the same in the legend of figure 3G, 4D, S3 C, S4 E, F; 5H, J; 6J, 7F as appropriate.
- Please note that information related to n is missing in the legends of figures S1 B, S5 C
- Please note that the error bars are not defined in the legend of figure S1 B.
- Please note that the scale bar needs to be defined for figures 4A', B'; S3 A, 5A', B', D', E'; 6A', B', D', E', F'; S5 A'; 7A', B, C'
We have updated all figure legends to state the number of replicates, the definitions for plots and statistical tests. Also, we have replaced asterisks for exact p-values where relevant, and we added scale bar definitions where needed.

- Please add scale bars of similar style and thickness to all microscopic images, using clearly visible black or white bars (depending on the background). Please place these in the lower right corner of the images themselves. Please do not write on or near the bars in the image but define the size in the respective figure legend. Presently, all scale bars have text nearby.
As requested, we updated scale bars to match the journal requirements.

- Please make sure that each final figure panel is called out separately and that the panels are called out sequentially. Presently, it seems there are no separate callouts for panels 1C, 2C, 7ABE and 8ABCDE. Please check. Moreover, please add callouts for the movies (see below).
We have updated the text or the figures to ensure that panels are called out individually and sequentially.

- The correct nomenclature for movie files is Movie EVx. Place change this in all places and add callouts for each file in the manuscript text. Please also provide legends for each movie. Each legend needs to be provided as separate text file and then uploaded, ZIPped together with its corresponding movie (so that we have 9 ZIP folders uploaded). Do not provide movie legends in the main text file.
We have renamed movie files following the EV format. We have uploaded the movies and their legends as individual .zip files as requested.

- Please make sure that all the funding information is also entered into the online submission system and that it is complete and similar to the one in the acknowledgement section of the manuscript text file. Presently, the grant number #IA202923 is provided in the manuscript text file, while IN203525 is entered in the submission system. Please check.
The correct grant number has been updated in the online system to match the one in the manuscript.

- Thanks for providing the source data. Please upload the numerical source data directly to the manuscript files, as one folder per main figure, grouping together the separate files (in excel format) for the panels with numerical data, and ZIPed together. Please make sure that the deposited image source data is public latest upon online publication of the paper.
We have organized and updated the data as requested.

In addition, I would need from you uploaded separately:
- a short, two-sentence summary of the manuscript (not more than 35 words).
- two to four short (!) bullet points highlighting the key findings of your study (two lines each).
- a schematic summary figure as separate file that provides a sketch of the major findings (not a data image) in jpeg or tiff format (with the exact width of 550 pixels and a height of not more than 400 pixels) that can be used as a visual synopsis on our website.

The requested information has been included in the submission.

I look forward to seeing the further revised version of your manuscript when it is ready. Please let me know if you have questions regarding the revision.

Please use this link to submit your revision: https://embor.msubmit.net/cgi-bin/main.plex

Best,

Achim Breiling
Senior Editor
EMBO Reports

Referee #1:

The authors have performed all the experiments requested, have clarified my doubts and answered to all my concerns. The manuscript has improved substantially since it was first submitted to Review Commons.

We thank the reviewer for their feedback, which greatly improved our work.

Referee #2:

This paper characterizes interactions between cells of the Drosophila embryonic trachea and nearby tendon and epidermal cells through pulling on a nearby ECM. The advance live imaging combined with electron microscopy is impressive and makes for a very detailed and valuable description, which is complemented by genetic experiments. The revised manuscript has been improved in clarity and benefitted from some additional, supporting genetic experiments and descriptions, which have strengthened the authors' arguments. Some n values remain a bit small, and the p values are presented with an unnecessary number of significant digits, so these could be improved. That said, all in all, the data are convincing and support the interesting idea that the ECM is mechanically coupling the development of several tissues. The manuscript will be of significant interest to those in the field and is worthy of being accepted for publication.

We are thankful for the positive feedback from the reviewer and for the comments on our manuscript, which have strengthened our work. We have standarised p values, keeping only two significant digits. In addition, to address the technical limitations that resulted in smaller sample sizes for certain experiments, we have ensured that all *n* values are explicitly stated and have used dot plots to clearly represent the distribution of the data.

Referee #3:

The authors did an excellent job addressing the concerns raised by all three reviewers and I would be delighted to see the paper published. My minor concerns are just small edits: Page 2, line 12, remove the comma after "to". Page 2, line 24 after "dorsally" use a ";" not a comma. Page 2, line 33, add a comma after "this". Page 4, line 5, add a comma after "development". Page 5, line 29, add the words "with antiserum" after embryos. Page 10, line three: the correct word is interface (not interphase). The authors should carefully check the rest of the manuscript for similar mistakes. I think I got too involved in thinking about the experiments to be as good about editing after a while.

We are grateful to the referee for their support of our manuscript and for pointing out these edits. As mentioned earlier, we have carefully proofread the manuscript. We modified the section where we explain our immunostainings and the suggestion about the word “antiserum” was no longer relevant. We think the manuscript is easier to follow now after incorporating all the comments from the reviewers.

Luis Daniel Rios-Barrera
Institute for Biomedical Research, Universidad Nacional Autonoma de Mexico
Cell Biology and Physiology Department
Mexico City 04510
Mexico

Dear Dr. Rios-Barrera,

I am very pleased to accept your manuscript for publication in the next available issue of EMBO reports. Thank you for your contribution to our journal.

Your manuscript will be processed for publication by EMBO Press. It will be copy edited and you will receive page proofs prior to publication. Please note that you will be contacted by Springer Nature Author Services to complete licensing and payment information.

You may qualify for financial assistance for your publication charges - either via a Springer Nature fully open access agreement or an EMBO initiative. Check your eligibility: https://link.springer.com/journal/44319/how-to-publish-with-us

Should you be planning a Press Release on your article, please get in contact with embo_production@springernature.com as early as possible in order to coordinate publication and release dates.

If you have any questions, please do not hesitate to contact the Editorial Office. Thank you for your contribution to EMBO Reports.

Yours sincerely,

Achim Breiling
Senior Editor
EMBO Reports

>>> Please note that it is EMBO Reports policy for the transcript of the editorial process (containing referee reports and your response letter) to be published as an online supplement to each paper. If you do NOT want this, you will need to inform the Editorial Office via email immediately. More information is available here: https://link.springer.com/partners/embo-press/editorial-policies#Peer%20review

EMBO Press Author Checklist

Corresponding Author Name: Luis Daniel Ríos Barrera	
Journal Submitted to: EMBO Reports	
Manuscript Number: EMBOR-2025-62287V1-T	

USEFUL LINKS FOR COMPLETING THIS FORM
The EMBO Journal - Author Guidelines
EMBO Reports - Author Guidelines
Molecular Systems Biology - Author Guidelines
EMBO Molecular Medicine - Author Guidelines

Reporting Checklist for Life Science Articles (updated January
This checklist is adapted from Materials Design Analysis Reporting (MDAR) Checklist for Authors. MDAR establishes a minimum set of requirements in transparent reporting in the life sciences (see Statement of Task: 10.31222/osf.io/9sm4x). Please follow the journal's guidelines in preparing your
Please note that a copy of this checklist will be published alongside your article.

Abridged guidelines for figures
1. Data
The data shown in figures should satisfy the following conditions:
- the data were obtained and processed according to the field's best practice and are presented to reflect the results of the experiments in an accurate and unbiased manner.
- ideally, figure panels should include only measurements that are directly comparable to each other and obtained with the same assay.
- plots include clearly labeled error bars for independent experiments and sample sizes. Unless justified, error bars should not be shown for technical
- if n<5, the individual data points from each experiment should be plotted. Any statistical test employed should be justified.
- Source Data should be included to report the data underlying figures according to the guidelines set out in the authorship guidelines on Data

2. Captions
Each figure caption should contain the following information, for each panel where they are relevant:
- a specification of the experimental system investigated (eg cell line, species name).
- the assay(s) and method(s) used to carry out the reported observations and measurements.
- an explicit mention of the biological and chemical entity(ies) that are being measured.
- an explicit mention of the biological and chemical entity(ies) that are altered/varied/perturbed in a controlled manner.
- the exact sample size (n) for each experimental group/condition, given as a number, not a range;
- a description of the sample collection allowing the reader to understand whether the samples represent technical or biological replicates (including how many animals, litters, cultures, etc.).
- a statement of how many times the experiment shown was independently replicated in the laboratory.
- definitions of statistical methods and measures:
  - common tests, such as t-test (please specify whether paired vs. unpaired), simple χ2 tests, Wilcoxon and Mann-Whitney tests, can be unambiguously identified by name only, but more complex techniques should be described in the methods section;
  - are tests one-sided or two-sided?
  - are there adjustments for multiple comparisons?
  - exact statistical test results, e.g., P values = x but not P values < x;
  - definition of 'center values' as median or average;
  - definition of error bars as s.d. or s.e.m.

Please complete ALL of the questions below.
Select "Not Applicable" only when the requested information is not relevant for your study.

Materials

Newly Created Materials	Information included in the manuscript?	In which section is the information available? (Reagents and Tools Table, Materials and Methods, Figures, Data Availability Section)
New materials and reagents need to be available; do any restrictions apply?	Not Applicable	

Antibodies	Information included in the manuscript?	In which section is the information available? (Reagents and Tools Table, Materials and Methods, Figures, Data Availability Section)
For **antibodies** provide the following information:<br>- Commercial antibodies: RRID (if possible) or supplier name, catalogue number and or/clone number<br>- Non-commercial: RRID or citation	Yes	Materials and Methods

DNA and RNA sequences	Information included in the manuscript?	In which section is the information available? (Reagents and Tools Table, Materials and Methods, Figures, Data Availability Section)
**Short novel DNA or RNA including primers, probes**: provide the sequences.	Not Applicable	

Cell materials	Information included in the manuscript?	In which section is the information available? (Reagents and Tools Table, Materials and Methods, Figures, Data Availability Section)
**Cell lines:** Provide species information, strain. Provide accession number in repository **OR** supplier name, catalog number, clone number, and/**OR** RRID.	Not Applicable	
**Primary cultures:** Provide species, strain, sex of origin, genetic modification status.	Not Applicable	
Report if the cell lines were recently **authenticated** (e.g., by STR profiling) and tested for mycoplasma contamination.	Not Applicable	

Experimental animals	Information included in the manuscript?	In which section is the information available? (Reagents and Tools Table, Materials and Methods, Figures, Data Availability Section)
**Laboratory animals or Model organisms:** Provide species, strain, sex, age, genetic modification status. Provide accession number in repository **OR** supplier name, catalog number, clone number, **OR** RRID.	Yes	Materials and Methods
**Animal observed in or captured from the field:** Provide species, sex, and age where possible.	Not Applicable	
Please detail **housing and husbandry conditions**.	Yes	Materials and Methods

Plants and microbes	Information included in the manuscript?	In which section is the information available? (Reagents and Tools Table, Materials and Methods, Figures, Data Availability Section)
**Plants:** provide species and strain, ecotype and cultivar where relevant, unique accession number if available, and source (including location for collected wild specimens).	Not Applicable	
**Microbes:** provide species and strain, unique accession number if available, and source.	Not Applicable	

Human research participants	Information included in the manuscript?	In which section is the information available? (Reagents and Tools Table, Materials and Methods, Figures, Data Availability Section)
If collected and within the bounds of privacy constraints report on age, sex and gender or ethnicity for all study participants.	Not Applicable	

Core facilities	Information included in the manuscript?	In which section is the information available? (Reagents and Tools Table, Materials and Methods, Figures, Data Availability Section)
If your work benefited from core facilities, was their service mentioned in the acknowledgments section?	Yes	Acknowledgements

Design

Study protocol	Information included in the manuscript?	In which section is the information available? (Reagents and Tools Table, Materials and Methods, Figures, Data Availability Section)
If study protocol has been **pre-registered, provide DOI in the manuscript**. For clinical trials, provide the trial registration number **OR** cite DOI.	Not Applicable	
Report the **clinical trial registration number** (at ClinicalTrials.gov or equivalent), where applicable.	Not Applicable	

Laboratory protocol	Information included in the manuscript?	In which section is the information available? (Reagents and Tools Table, Materials and Methods, Figures, Data Availability Section)
Provide DOI OR other citation details if **external detailed step-by-step protocols** are available.	Yes	Materials and Methods

Experimental study design and statistics	Information included in the manuscript?	In which section is the information available? (Reagents and Tools Table, Materials and Methods, Figures, Data Availability Section)
Include a statement about **sample size** estimate even if no statistical methods were used.	Yes	Materials and Methods
Were any steps taken to minimize the effects of subjective bias when allocating animals/samples to treatment (e.g. **randomization procedure**)? If yes, have they been described?	Not Applicable	
Include a statement about **blinding** even if no blinding was done.	Yes	Materials and Methods
Describe **inclusion/exclusion criteria** if samples or animals were excluded from the analysis. Were the criteria pre-established?<br><br>If sample or data points were omitted from analysis, report if this was due to attrition or intentional exclusion and provide justification.	Not Applicable	
For every figure, are **statistical tests** justified as appropriate? Do the data meet the assumptions of the tests (e.g., normal distribution)? Describe any methods used to assess it. Is there an estimate of variation within each group of data? Is the variance similar between the groups that are being statistically compared?	Yes	Materials and Methods and Figure Legends

Sample definition and in-laboratory replication	Information included in the manuscript?	In which section is the information available? (Reagents and Tools Table, Materials and Methods, Figures, Data Availability Section)
In the figure legends: state number of times the experiment was **replicated** in laboratory.	Yes	Matherials and Methods
In the figure legends: define whether data describe **technical or biological replicates**.	Not Applicable	

Ethics

Ethics	Information included in the manuscript?	In which section is the information available? (Reagents and Tools Table, Materials and Methods, Figures, Data Availability Section)
Studies involving **human participants**: State details of **authority granting ethics approval** (IRB or equivalent committee(s), provide reference number for approval.	Not Applicable	
Studies involving **human participants**: Include a statement confirming that **informed consent** was obtained from all subjects and that the experiments conformed to the principles set out in the WMA Declaration of Helsinki and the Department of Health and Human Services Belmont Report.	Not Applicable	
Studies involving **human participants:** For publication of **patient photos**, include a statement confirming that consent to publish was obtained.	Not Applicable	
Studies involving experimental **animals**: State details of **authority granting ethics approval** (IRB or equivalent committee(s), provide reference number for approval. Include a statement of compliance with ethical regulations.	Not Applicable	
Studies involving **specimen and field samples:** State if relevant **permits** obtained, provide details of authority approving study; if none were required, explain why.	Not Applicable	

Dual Use Research of Concern (DURC)	Information included in the manuscript?	In which section is the information available? (Reagents and Tools Table, Materials and Methods, Figures, Data Availability Section)
Could your study fall under dual use research restrictions? Please check biosecurity documents and list of **select agents and toxins** (CDC): https://www.selectagents.gov/sat/list.htm	Not Applicable	
If you used a select agent, is the security level of the lab appropriate and reported in the manuscript?	Not Applicable	
If a study is subject to dual use research of concern regulations, is the name of the **authority granting approval and reference number** for the regulatory approval provided in the manuscript?	Not Applicable	

Reporting

The MDAR framework recommends adoption of discipline-specific guidelines, established and endorsed through community initiatives. Journals have their own policy about requiring specific guidelines and recommendations to complement MDAR.

Adherence to community standards	Information included in the manuscript?	In which section is the information available? (Reagents and Tools Table, Materials and Methods, Figures, Data Availability Section)
State if relevant guidelines or checklists (e.g., **ICMJE, MIBBI, ARRIVE, PRISMA**) have been followed or provided.	Not Applicable	
For **tumor marker prognostic studies,** we recommend that you follow the **REMARK** reporting guidelines (see link list at top right). See author guidelines, under 'Reporting Guidelines'. Please confirm you have followed these guidelines.	Not Applicable	
For **phase II and III randomized controlled trials**, please refer to the **CONSORT** flow diagram (see link list at top right) and submit the CONSORT checklist (see link list at top right) with your submission. See author guidelines, under 'Reporting Guidelines'. Please confirm you have submitted this list.	Not Applicable	

Data Availability

Data availability	Information included in the manuscript?	In which section is the information available? (Reagents and Tools Table, Materials and Methods, Figures, Data Availability Section)
Have **primary datasets** been deposited according to the journal's guidelines (see 'Data Deposition' section) and the respective accession numbers provided in the Data Availability Section?	Yes	Data Availability Section
Were **human clinical and genomic datasets** deposited in a public access-controlled repository in accordance to ethical obligations to the patients and to the applicable consent agreement?	Not Applicable	
Are **computational models** that are central and integral to a study available without restrictions in a machine-readable form? Were the relevant accession numbers or links provided?	Not Applicable	
If publicly available data were reused, provide the respective **data citations in the reference list**.	Not Applicable	
